# Autonomous Synthesis of Nanoparticles with Target Scattering Patterns

**DOI:** 10.1021/acsnano.5c15488

**Published:** 2026-02-18

**Authors:** Andy S. Anker, Jonas H. Jensen, Miguel González-Duque, Rodrigo Moreno, Aleksandra Smolska, Mikkel Juelsholt, Vincent Hardion, Mads R. V. Jørgensen, Andrés Faíña, Jonathan Quinson, Kasper Støy, Tejs Vegge

**Affiliations:** † Department of Energy Conversion and Storage, Technical University of Denmark, Kgs Lyngby 2800, Denmark; ‡ Department of Chemistry, University of Oxford, Oxford OX1 3TA, U.K.; § Department of Computer Science, IT University of Copenhagen, 2300 Copenhagen, Denmark; ∥ Department of Biology, University of Copenhagen, Copenhagen 2200, Denmark; ⊥ Biological and Chemical Engineering Department, Aarhus University, Aarhus 8200, Denmark; # Department of Chemical Engineering, Columbia University, New York, New York 10027, United States; ¶ MAX IV Laboratory, Lund University, Lund 225 94, Sweden; ∇ Department of Chemistry and iNANO, Aarhus University, Aarhus C 8000, Denmark; ○ CICA-Centro Interdisciplinar de Química e Bioloxía, Facultade de Ciencias, Universidade da Coruña, Campus de Elviña, Coruña 15008 A, Spain

**Keywords:** self-driving laboratories, autonomous laboratories, robotic synthesis, nanomaterials, X-ray scattering, machine learning, synchrotrons

## Abstract

Controlled synthesis of materials with specified atomic structures underpins technological advances yet remains reliant on iterative, trial-and-error approaches. Nanoparticles (NPs), whose atomic arrangement dictates their emergent properties,^1–5^ are particularly challenging to synthesize due to numerous tunable parameters. Here, we introduce an autonomous approach that explicitly targets atomic-scale structure through scattering patterns. Our method autonomously designs synthesis protocols by matching real-time experimental total scattering (TS) and pair distribution function (PDF) data to simulated target patterns, without requiring embedded synthesis knowledge. We demonstrate this capability at a synchrotron by targeting two structurally distinct gold NP scattering patterns: 5 nm decahedral and 10 nm face-centered cubic structures. Ultimately, specifying target scattering patterns and autonomously approaching synthesis protocols that reproduce them experimentally may enable on-demand, atomic structure-informed materials design. ScatterLab thus provides a generalizable blueprint for autonomous, atomic structure-targeted synthesis across diverse systems and applications.

## Graphical abstract

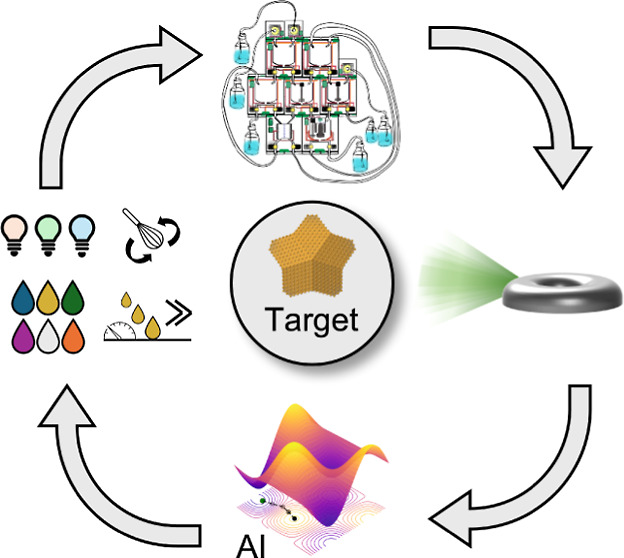

## Introduction

Controlling the atomic structure of materials is essential for tailoring their functional properties, yet synthesis protocols for them often remain the result of laborious, trial-and-error optimization. NPs are a prime example: their optical, catalytic, and electronic properties are governed by size, shape, and atomic arrangement.^1–5^ Gold nanoparticles (AuNPs), in particular, are widely studied for applications in catalysis and medicine owing to their tunable optical response, chemical stability, and biocompatibility.^6,7^ Notably, the size and degree of twinning in AuNPs influence their catalytic performance.^8,9^ Despite a century of reported synthetic strategies, achieving atomic structure control and monodispersity at higher concentrations remain a daunting task,^10,11^ largely because many synthesis parameters (e.g., reagent ratios, additives, temperature, time, and illumination conditions) must be tuned simultaneously.

Self-driving laboratories (SDLs) offer an attractive route to accelerate such optimization by autonomously proposing and executing experiments based on real-time feedback.^12^ AuNPs have become a common model system for SDL development,^13–18^ as their synthesis chemistry is well studied and their morphology can be conveniently monitored by UV–Vis spectroscopy. Tracking plasmonic absorption enables indirect inference of morphology, but this strategy has intrinsic limitations: many materials and NP systems lack strong plasmonic signatures, different morphologies can produce overlapping spectra, and UV–Vis spectroscopy alone cannot resolve atomic structure. Recent SDL advances have incorporated scattering-based measurements—such as X-ray reflectometry^19^ or X-ray diffraction^20,21^ —but these techniques are constrained to flat films or materials with substantial long-range order. Neither UV–Vis, reflectometry, nor diffraction techniques provide a universal characterization technique for autonomous synthesis of nanoscale or structurally complex materials.

By contrast, TS extends conventional diffraction by measuring to large scattering vectors and retaining both Bragg and diffuse contributions; Fourier transformation of this signal yields the PDF. This makes TS/PDF applicable virtually any material, whether crystalline or noncrystalline, across scales from atomic to macroscopic.^22,23^ TS/PDF has been applied to systems ranging from molecules,^24,25^ clusters in solution,^26,27^ nanomaterials,^28,29^ disordered phases,^30,31^ amorphous solids,^32,33^ crystalline solids,^34,35^ porous frameworks,^32,36^ and layered or two-dimensional materials.^37,38^ Since TS and its real-space PDF encode reciprocal- and real-space atomic pair correlations, deviations from the intended structure, such as particle size, atomic structure, disorder, impurities, or structural heterogeneity, manifest directly in the measured data. As in conventional diffraction, scattering features reflect atomic structure; however, whereas periodic atomic order gives rise to sharp Bragg peaks, finite crystallite size and structural disorder broaden the signal into diffuse scattering. In real space, short-range PDF features probe local atomic environments, whereas longer-range correlations encode the overall material structure, with their damping at larger distances reflecting finite particle size. This ability to capture both short- and longer-range order makes TS/PDF an appealing technique for general-purpose structural feedback in SDLs, including for AuNPs where atomic arrangement is crucial for function.^8,9^ The main disadvantage is the need for synchrotron facilities to obtain rapid measurements, which introduces practical and logistical constraints (section A, Supporting Information), although in-house PDF instruments already provide data quality sufficient for SDL workflows but orders of magnitude slower.

Here, we introduce ScatterLab, an SDL designed to autonomously discover synthesis protocols that yield materials whose experimental TS/PDF patterns match a user-specified “target” data set. ScatterLab integrates (i) our newly developed **MOD**ular **EX**perimentation platform (MODEX) designed for rapid instruments interfacing, (ii) synchrotron-based TS/PDF measurements for real-time structural feedback, and (iii) a Bayesian Optimisation (BO) algorithm running on a high-performance computing (HPC) system. A schematic overview is given in Figure 1, with technical details in the Methods section. As a proof-of-concept demonstration, we deployed ScatterLab at the DanMAX beamline (MAX IV synchrotron). Over a four-day campaign (plus one day without X-rays), we installed ScatterLab and targeted TS/PDF patterns simulated from two deliberately contrasting AuNP structures: a ∼5 nm decahedral NP and a spherical 10 nm face-centered cubic (FCC) NP. Under the assumption^39^ that identical scattering profiles are representations of the same atomic structure, this procedure yields nanoscale arrangements closely matching those prescribed by the target data set. In information-theoretic-terms, the TS/PDF can be viewed as a high-information projection of the underlying atomic arrangement, consistent with the recent concept of ‘tomographic’ interpretations of materials representations.^5,40^ Rather than relying on prescribed synthesis recipes, ScatterLab autonomously navigates an 11-dimensional parameter space spanning six reagents, mixing speeds, Au precursor addition rates, and white/UV–B/UV–C illumination and may even exclude reagents that do not contribute to optimization. These two structures were chosen as structurally distinct test cases with separable TS/PDF signatures, enabling a proof-of-concept demonstration of atomic-structure-targeted optimization. Since atomic arrangement influence catalytic properties,^9^ demonstrating autonomous structural control is broadly relevant for the design of functional nanomaterials.

**Figure 1:**
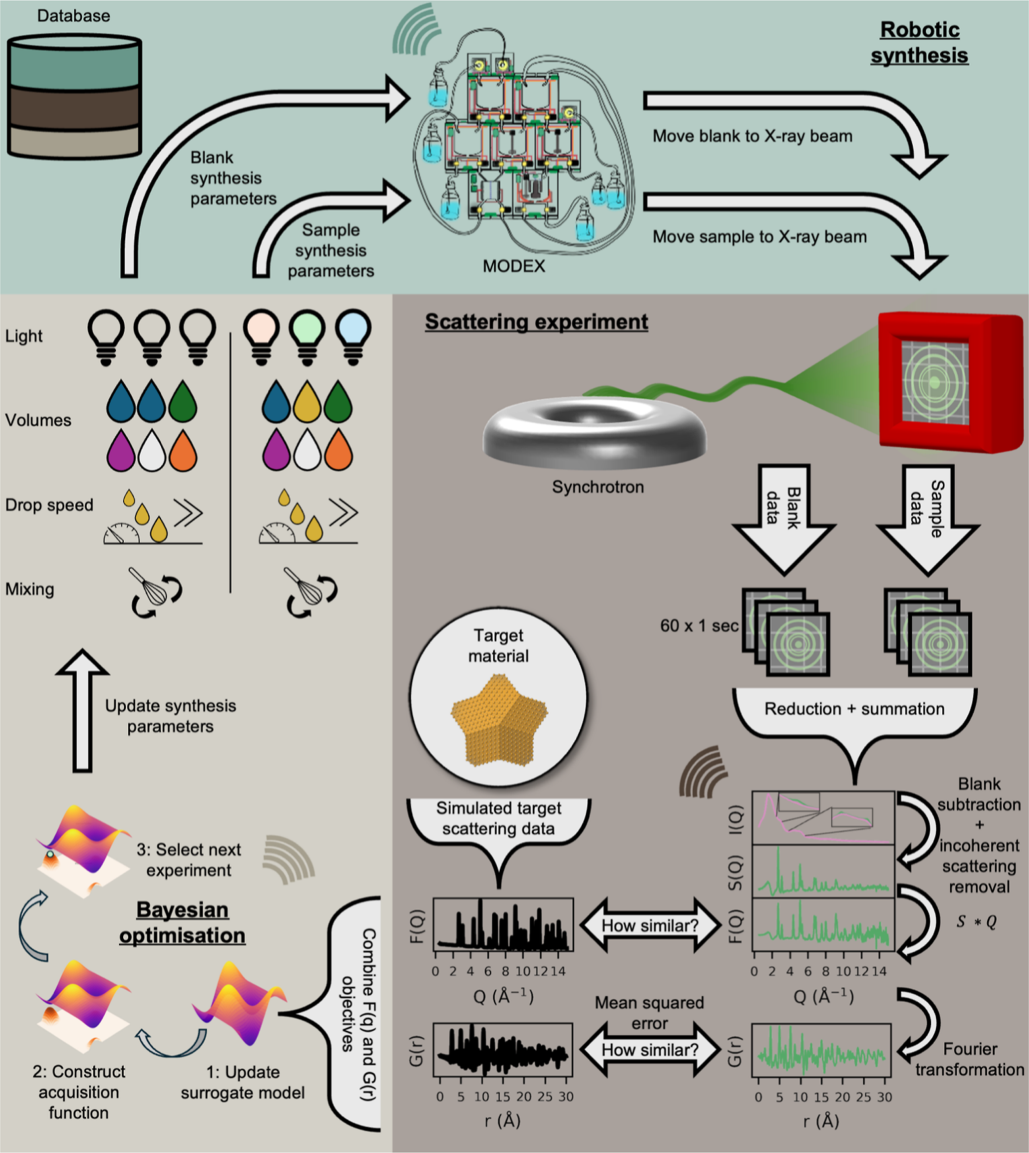
Methodology of ScatterLab: robotic synthesis, scattering experiment, and BO. ScatterLab begins with target scattering patterns in both Q-space and r-space as the target objective. It then selects synthesis parameters—volumes of chemicals, Au precursor addition speeds, light-emitting diode (LED) illumination intensities (white, UV–B, and UV–C, for fixed 5 min), and mixing speed—which MODEX executes. First, a “blank” sample (without the metal precursor, also called background) is transferred to the X-ray beam for a one-minute measurement. Next, the corresponding sample is measured similarly. An automated trigger initiates data reduction, summation, blank subtraction, and postprocessing to obtain blank subtracted scattering intensities, I­(Q), the total scattering structure function, S­(Q), the reduced total scattering function, F­(Q), and the reduced atomic pair distribution function, G­(r). The difference between the processed scattering patterns and the target scattering patterns is then computed as an objective value. BO updates the surrogate model, constructs the acquisition function, and proposes the next experiment, thereby refining the synthesis parameters. All synthesis metadata, scattering patterns, and BO results are recorded in an external MongoDB database on the fly.

We compare ScatterLab with alternative NP synthesis strategies (section B, Supporting Information) and specifically other SDLs^13–18^ that optimize AuNP synthesis; using AuNPs here as a model system (section C, Supporting Information). Synchrotron safety and time constraints necessitated a distinct synthetic strategy compared with prior studies (section D, Supporting Information). Our rapid (5 min), room-temperature UV-induced reactions use safe chemicals, while producing monodisperse AuNPs at higher concentrations (3.5 mM versus typically <1 mM). Importantly, by relying on TS/PDF instead of UV–Vis, ScatterLab directly targets atomic structure and can, in principle, be applied to materials well beyond AuNPs. Ultimately, specifying a simulated target scattering pattern—representing an intended atomic structure—and then obtaining both the synthesized material and its synthesis protocol on demand may revolutionize the design, discovery, and deployment of next-generation materials. Since TS/PDF is inherently general, ScatterLab provides an immediately transferable blueprint for fully autonomous, atomic-structure-targeted synthesis across diverse chemical systems.

## Results

In silico BO benchmarking: optimizing hyperparameters of ScatterLab.

Ideally, the choice of BO algorithm would be guided by large-scale experimental benchmarking campaigns, where different optimizers are compared across many physical optimization runs. This is currently impractical for TS/PDF workflows, where each optimization campaign is resource-intensive. Instead, we performed an in silico benchmarking study comprising 540 BO campaigns. In these simulations, we systematically varied several hyperparameters: the choice of BO algorithm, the scattering domain used for optimization, the normalization strategy for the scattering data, and the objective function. Each configuration was tested against three target data sets using five different random seeds. Descriptions of these procedures results, and differences to the actual experiments are provided in section E in the Supporting Information. From this in silico benchmarking effort, we concluded that employing both Q-space and r-space data, normalizing to the highest peak, and using mean-squared error (MSE) as the objective function in conjunction with the Sparse Axis-Aligned Subspaces Bayesian Optimization (SAASBO^41^) optimizer provided the most robust performance. This strategy formed the basis of our experimental approach at the synchrotron, optimizing the likelihood of rapidly converging to the desired AuNP structure under real experimental conditions.

### Autonomous Synthesis of 5 nm Decahedral AuNPs

Although bulk Au typically adopts an FCC lattice, nanosized Au particles can form geometries such as icosahedral, octahedral, or decahedral morphologies,^9,42,43^ which differ in how atomic layers stack and how surfaces are truncated or twinned. We next applied ScatterLab to experimentally synthesize AuNPs designed to reproduce the scattering patterns of a Marks decahedral structure with size of ∼5 nm (4.9 × 4.9 × 4.3 nm) (Figure 2A). Note that this is a theoretically constructed XYZ file (consisting of atomic elements and Euclidian coordinates) and there is no guarantee that this specific Marks decahedral is synthesisable. Our goal was to determine whether ScatterLab could be guided to synthesize these ∼5 nm decahedral AuNPs or any similar structures by targeting its simulated scattering pattern (Figure 2B). The target scattering patterns were computed using DebyeCalculator^44^ with a *Q*_range_ of 0.5–15 Å^–1^ (*Q*_step_: 0.01 Å^–1^), an *r*_range_ of 0–30 Å (*r*_step_: 0.01 Å), and the isotropic atomic displacement parameters in B (ADPs) set to 0.3 Å^2^.

**Figure 2:**
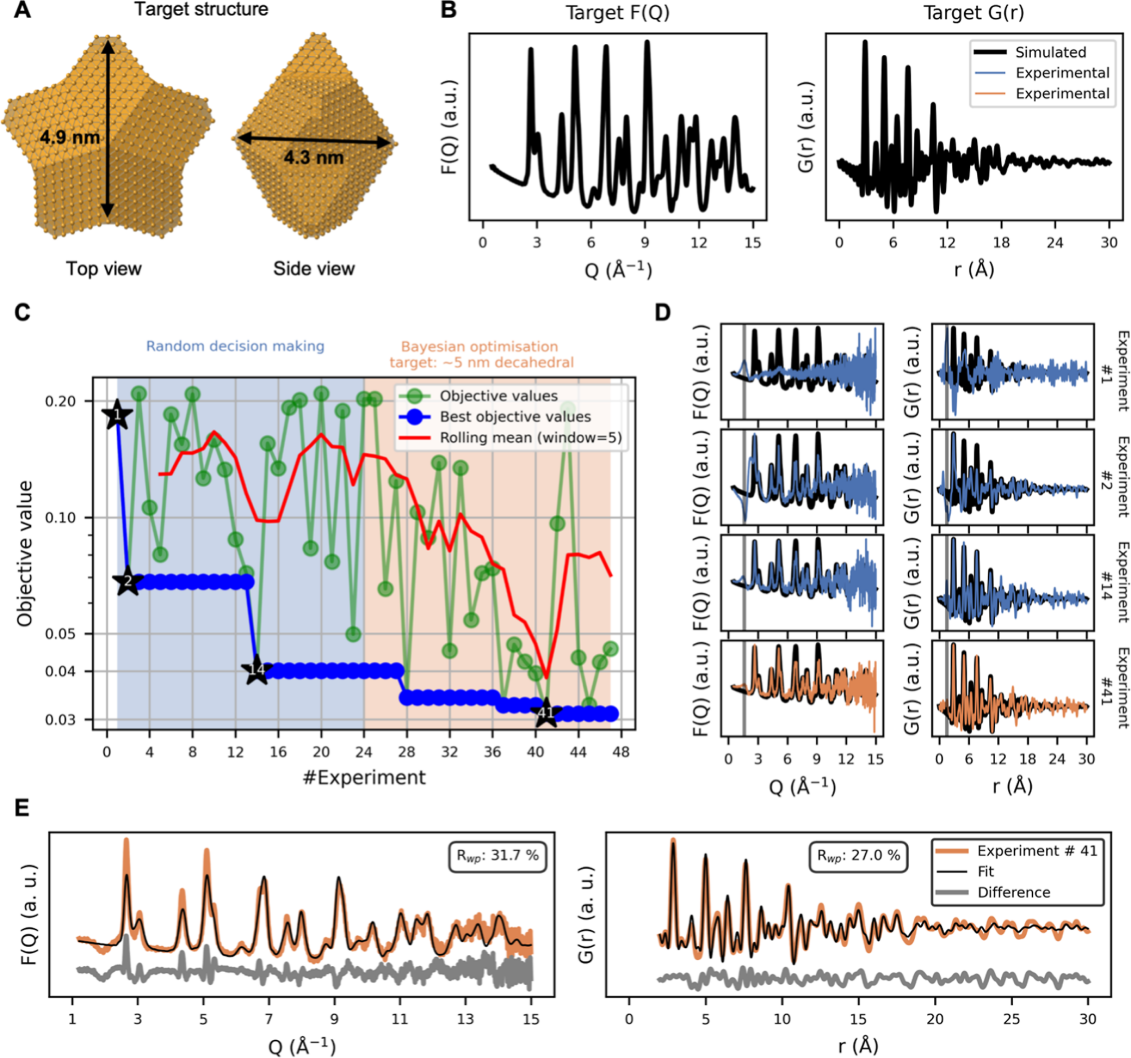
Autonomous synthesis of ∼5 nm decahedral AuNPs. (A) Three-dimensional representation of the ∼5 nm (4.9 × 4.9 × 4.3 nm) target decahedral AuNP, visualized using CrystalMaker.^50^ (B) Corresponding target scattering patterns in reciprocal space, F­(Q), and real space, G­(r), simulated from the decahedral structure using DebyeCalculator.^44^ (C) Objective values for each experiment. The blue shaded region denotes the initial 24 experiments with randomly selected synthesis parameters and the orange shaded region indicates BO experiments that used the ∼5 nm decahedral target pattern. (D) Selected experimental scattering patterns overlaid on the simulated ∼5 nm decahedral target data. The gray vertical line highlights signal contributions from unreacted precursor and solvent. (E) Combined refinement of the ∼5 nm decahedral target structure to the experimental F­(Q) and G­(r) data acquired over 15 min with identical conditions as experiment #41.

To accommodate the tight constraints of a synchrotron beamline, we used our newly developed MODEX platform comprising decimeter-scale modules, each dedicated to a distinct chemical unit operation (e.g., liquid handling, mixing, light illumination). This design allowed us to assemble, calibrate, and test the setup beforehand, then transport and reassemble it on-site at the synchrotron within just a few hours. For the synthesis, ScatterLab navigated an 11-dimensional synthesis space by varying the volumes of H_2_O, glycerol, ethanol, HAuCl_4_, NaOH, and sodium citrate tribasic dihydrate (NaCt); modulating HAuCl_4_ addition speeds; adjusting LED illumination intensities (white, UV-B, and UV-C, applied for a fixed duration of 5 min); and controlling mixing speeds. Each TS measurement was acquired over a fixed duration of 1 min. The optimization campaign began with 24 experiments in which synthesis parameters were selected at random to widely sample the chemical parameter space. Figure 2C presents the objective values—quantifying how closely the experimental scattering patterns match the target data—across all experiments. A lower objective value indicates a closer agreement with the target scattering pattern (see Methods, “objective function”). The green points show the objective values for individual experiments, the blue points track the best (lowest) objective value obtained so far, and the red line represents the rolling mean of the objective values. During the initial random phase (first 24 experiments), the rolling mean remains flat, reflecting that the parameter space is being broadly explored rather than systematically optimized.

Most random experiments, such as experiment #1 (Figure 2D), yield an objective value around 0.2, which corresponds to conditions where no NP forms and the scattering pattern primarily reflects unreacted precursors and solvents. Such patterns feature a broad peak at ∼1.6 Å^–1^ in F­(Q) and a peak at ∼1.5 Å in G­(r). However, some random experiments do produce lower objective values (∼0.1). For instance, experiment #2 yields a partially formed AuNP—albeit not one that matches the target structure—and experiment #14 obtains data that better match the simulated target scattering patterns, suggesting more well-defined AuNPs and fewer byproducts (e.g., no or undetectable unwanted minority products). Notably, the final metal concentration in experiment #14 is only 2.06 mM, considerably below the previously reported detection limit at 10 mM for TS at the DanMAX beamline.^45^ By comparison, low-concentration TS data sets at other facilities typically range 4.5–30 mM,^46–48^ whereas many spectroscopic techniques (e.g., UV–Vis) operate effectively only at lower concentrations and often provide less direct structural detail. Nevertheless, by measuring both sample and blank in the same capillary, we achieve precise blank/background subtraction, thereby enabling structural insights at concentrations down to 1 mM.

After the initial random exploration, we trained a surrogate model on the 24 randomly acquired parameters/objectives pairs and initiated the BO-driven optimization. Immediately upon switching from random selection to BO-based parameter proposals, the rolling mean of the objective values declined, indicating that the SDL is now leveraging the surrogate model to make informed synthesis decisions. By experiment #28, with only four additional experiments, the BO approach has already identified synthesis parameters that outperform all random attempts. Further optimization leads to experimental scattering patterns that more closely resembles the target scattering patterns (experiment #41) indicating that the synthesized AuNPs can be described by the ∼5 nm target decahedral structure. At experiment #47, optimization was stopped because the objective values had not improved over six consecutive experiments, although additional beamtime could in principle reveal further improvement to the synthesis conditions. The improved signal-to-noise ratio at experiment #41 reflects that ScatterLab increases the AuNP concentration, while simultaneously reducing the amount of unreacted precursor, as evidenced by the diminishing broad peaks at ∼1.6 Å^–1^ in F­(Q) and ∼1.5 Å in G­(r). Because the simulated targets represent a single-phase, noise-free structure, any secondary phase would introduce additional features in the scattering profiles, which the optimization naturally suppresses as it steers the synthesis toward a phase-pure, low-noise signal; in future implementations, both noise reduction and yield could also be incorporated explicitly through multiobjective optimization.

We further demonstrate repeatability (consistency within a single setup) by repeating experiments under identical synthesis conditions and obtaining consistent TS/PDF profiles (section F, Supporting Information), while reproducibility (consistency across different setups) remains to be established in the SDL literature. For the repeated experiment #41, we also performed a longer measurement of 15 min to improve the statistical quality of the scattering signal. Figure 2E shows a combined fit of the target structure to the F­(Q) and G­(r) from the extended measurement, using a Debye-based calculation to account for finite-cluster effects (see Methods, “Finite clusters”). In addition, we performed an extensive cluster-mining search^49^—covering 1965 decahedral, icosahedral, octahedral, and FCC motifs—to identify structures that best describe the experimental data (section G, Supporting Information). This analysis consistently favors decahedral motifs, with the target model ranking among the top candidates.

Although a Debye-based comparison evaluates each scattering pattern against a single idealized cluster and the real sample may contain a distribution of sizes or shapes, the high level of agreement between the experimental and target TS/PDF profiles indicates that ScatterLab approached synthesis conditions yielding a decahedral-like atomic arrangement consistent with the prescribed target scattering pattern. Crucially, while we cannot rule out structurally related alternatives, the measured TS/PDF profiles closely match the simulated target TS/PDF patterns, indicating that ScatterLab approached synthesis conditions yielding an atomic arrangement consistent with the intended decahedral structure. Additional morphological insight could be gained by integrating complementary techniques such as small-angle X-ray scattering (SAXS) or transmission electron microscopy (TEM).

### Autonomous Synthesis of Spherical 10 nm FCC AuNPs

Having demonstrated ScatterLab’s capacity to approach synthesis conditions yielding scattering patterns consistent with bespoke ∼5 nm decahedral AuNPs, we next sought to produce larger, spherical 10 nm AuNPs adopting an FCC structure (Figure 3A). Specifically, we tested whether ScatterLab could leverage previously acquired experimental data from the smaller-particle synthesis to guide parameter selection for the new, larger FCC targets. To this end, we simulated target scattering patterns (Figure 3B) representing the desired 10 nm FCC AuNP, generating F­(Q) and G­(r) functions again using a *Q*_range_ of 0.5–15 Å^–1^ (*Q*_step_ = 0.01 Å^–1^) and an *r*_range_ of 0–30 Å (*r*_step_ = 0.01 Å), with isotropic ADPs of 0.3 Å^2^ using the DebyeCalculator software.^44^

**Figure 3:**
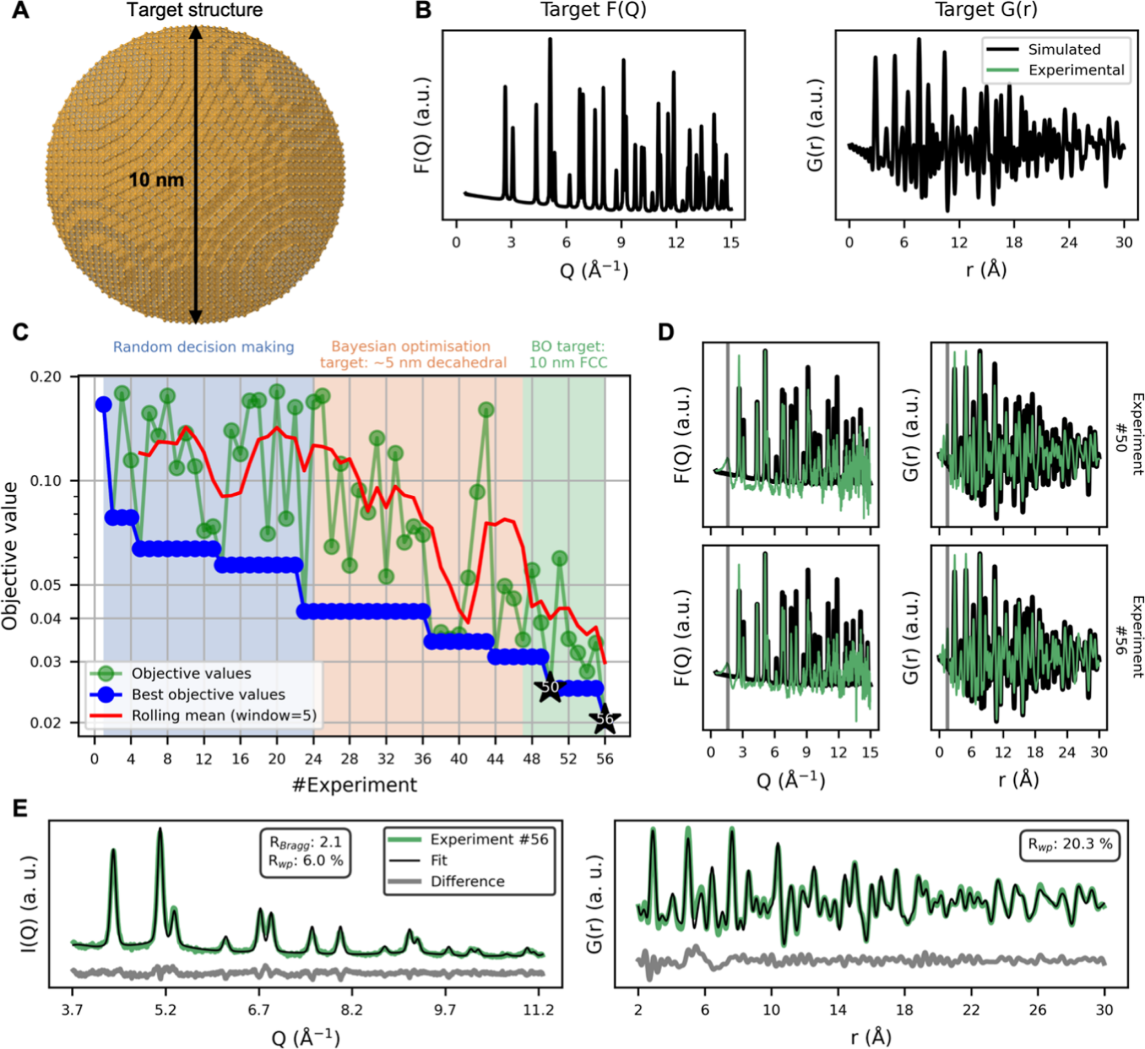
Autonomous synthesis of spherical 10 nm FCC AuNPs. (A) Three-dimensional representation of the spherical 10 nm FCC AuNP target, visualized using CrystalMaker.^50^ (B) Corresponding target scattering patterns in reciprocal space, F­(Q), and real space, G­(r), simulated from the FCC model using DebyeCalculator.^44^ (C) Objective values for each experiment. The blue shaded region denotes the initial 24 experiments with randomly selected synthesis parameters. The orange shaded region indicates BO experiments that continued to use the ∼5 nm decahedral pattern as target, while the teal shaded region marks the switch to the spherical 10 nm FCC target. (D) Selected experimental scattering patterns overlaid on the simulated spherical 10 nm FCC target data. The gray vertical line highlights signal contributions from solvent and unreacted precursor. (E) Combined Rietveld and real-space Rietveld refinement of the FCC target structure to the experimental I­(Q) and G­(r) data from experiment #56.

Instead of initiating the new campaign with random experiments, we transferred prior knowledge gained from the earlier synthesis trials. Specifically, we reused the parameter sets from the first campaign but recalculated their objective values against the spherical 10 nm FCC AuNP target’s scattering patterns, thus starting the second campaign with a pretrained surrogate model. Figure 3C shows the progression of objective values across all experiments. Once the updated BO approach is deployed for the 10 nm target, the rolling mean again decreases—indicating that knowledge gained about producing any NP helps guide the search toward parameters favoring the 10 nm FCC configuration.

After starting the BO on the new target data sets, ScatterLab rapidly converges toward synthesis conditions producing AuNPs closer to the new target structure. Experiments #50 and #56 exemplifies this improvement (Figure 3D). Both yield scattering patterns that more closely match the simulated 10 nm AuNP target data. Again, we note that by running ScatterLab for longer (experiment #56 versus experiment #50), it further minimizes byproducts as indicated by the diminished broad peak near ∼1.6 Å^–1^ in F­(Q) and the decrease of the ∼1.5 Å peak in G­(r). Notably, while the Au concentration remains constant across these runs, the optimized synthesis conditions lead to more complete precursor conversion, ultimately improving data-to-noise ratio. The difference between experiments #50 and #56 demonstrates that ScatterLab learns that larger NPs is produced with lower amount of citrate and in presence of less reducing agent (glycerol) and higher concentrations of NaOH leads to larger NPs.^51,52^

Building on these findings, Figure 3E shows a combined Rietveld refinement of an FCC model against the experimental I­(Q) and G­(r) data for experiment #56 (see Methods, “Attenuated crystal approximation”). The fit exhibits good agreement across both reciprocal and real space, indicating that an FCC phase provides an excellent description of the measured scattering data. The refined diameter is 7.5 nm—smaller than the nominal 10 nm target—but the final data point was collected at 07:22 AM, shortly before our allocated beamtime ended at 08:00 AM, leaving no opportunity for further optimization (section A, Supporting Information). With additional beamtime, we anticipate ScatterLab would continue the observed trend of increasing NaOH and decreasing NaCt to drive the system toward larger, more target-like particles. Despite these constraints, the experiment demonstrates that ScatterLab again identified synthesis conditions that yield scattering patterns closely matching those simulated for an FCC target.

### Behind the Scenes: How ScatterLab Navigates AuNP Synthesis Variables

We next examine how ScatterLab navigates the available synthesis conditions to achieve the target scattering patterns. Figure 4 shows nine of the eleven optimization dimensions plotted against the resulting objective values from all 56 experiments. These plots show individual parameter trends; identifying higher-order interactions would require many more experiments. We therefore treat them as qualitative indicators. White LED intensity and mixing speed are also recorded but omitted here for clarity; their usage patterns are provided in section H of the Supporting Information. In the top three panels of Figure 4, we show the relative volumes of water, glycerol, and ethanol, which sum to 100%. During the initial random exploration phase (blue points), ScatterLab sampled a wide range of the parameter space. Once BO was initiated, it began optimizing the synthesis conditions for two specific targets: ∼5 nm decahedral AuNPs (orange points) and subsequently toward 10 nm spherical FCC AuNPs (green points). Interestingly, the BO algorithm favored excluding ethanol, consistent with reports suggesting faster AuNP formation in glycerol-rich media.^53^ This behavior may also reflect the five-minute reaction window, which limits the time available for ethanol-driven reduction pathways.

**Figure 4:**
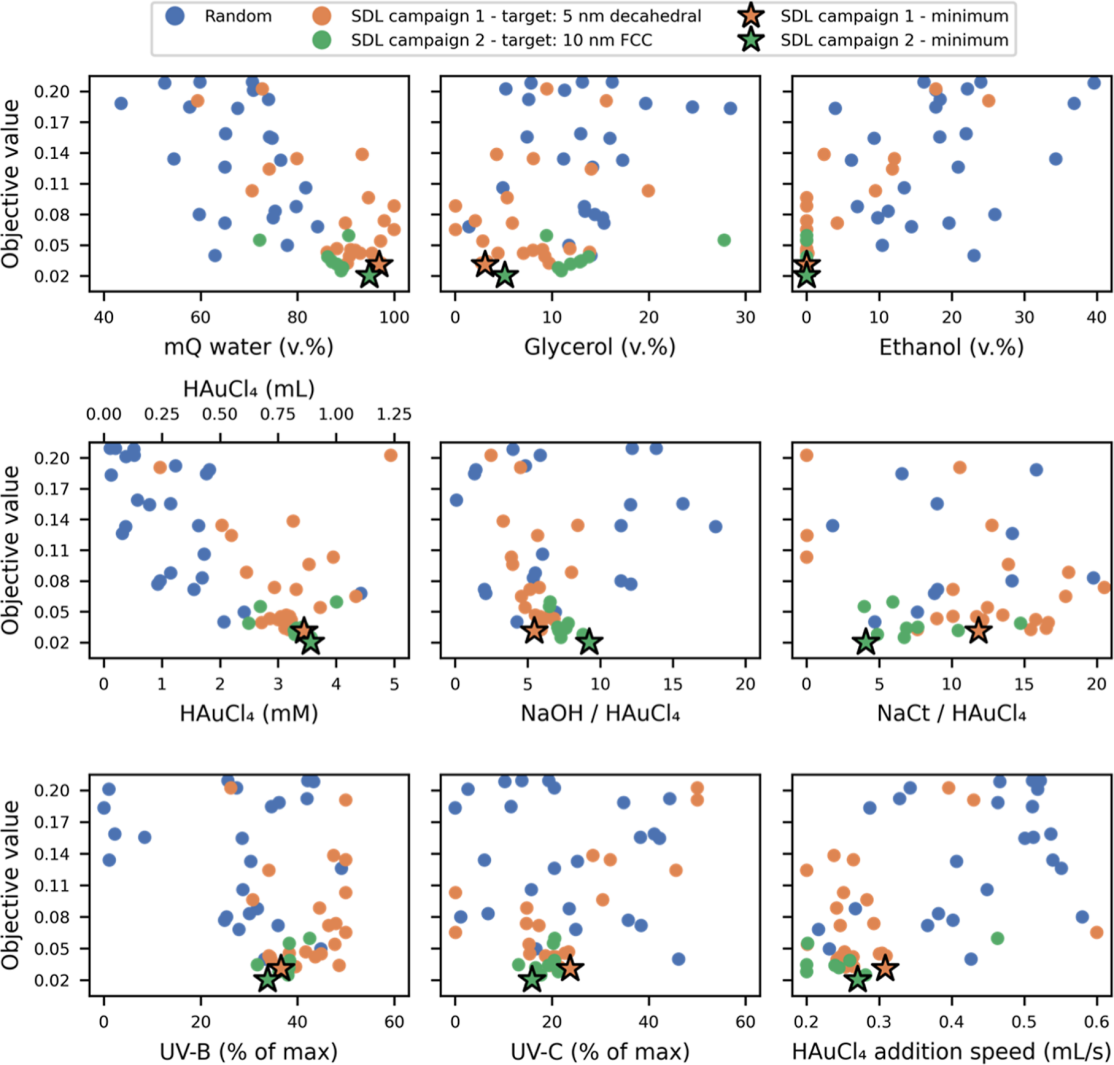
Synthesis parameters versus objective values. Top panels: water (left), glycerol (middle), and ethanol (right) content in v.%. Middle panels: HAuCl_4_ content in mM (bottom *X* axis) and mL (upper *X* axis) (left), NaOH/HAuCl_4_ (middle), and NaCt/HAuCl_4_ (right) ratios plotted between 0 and 20. Lower panels: UV–B (left), and UV–C (middle) lamp power (100% corresponds to full intensity), and HAuCl_4_ precursor addition speed (mL/s) (right). All plotted against the objective values for each experiment. Objective values for the random and decahedral experiments (blue, orange) are evaluated against the decahedral target; FCC experiments (green) are evaluated against the FCC target. Values can therefore be compared across campaigns only with care. Section H, Supporting Information, represents the same plot without capping the *x*-axis at 20 in the NaOH/HAuCl_4_ and NaCt/HAuCl_4_ ratios, alongside additional figures highlighting the added volume of each chemical for each experiment and parameter variations over the course of the experiments. An interactive plot of the Figure is shared as part of the associated code.

The middle panels show the ratios of NaOH/HAuCl_4_ and NaCt/HAuCl_4_, along with the Au precursor concentrations. These variables are well-known to control AuNP nucleation and growth.^52^ Remarkably, ScatterLab can produce AuNPs at concentrations about 3.5 mM—exceeding the typical 1 mM limit.^10^ When the target changes from 5 to 10 nm particles (orange to green points), the system increases NaOH and decreases NaCt, both consistent with literature expectations: slower precursor reduction and reduced steric stabilization favor larger AuNPs.^52^ The lower panels display UV lamp power and the HAuCl_4_ addition speed. The system converges on intermediate UV–B and UV–C intensities, probably balancing the need for rapid reduction with the risk of excessive heating. The white light, by contrast, is nearly deactivated. The best-performing experiments often employed “slow” HAuCl_4_ addition rates of ∼0.3 mL/s, which practically speaking is relatively fast compared to manual addition in conventional laboratory protocols, where rapid precursor injections are typically preferred.^52,54^ These chemical insights highlight the advantage of having an autonomous platform that systematically monitors and explores a broad parameter space, potentially uncovering nontraditional AuNP synthesis protocols.

Ultimately, we attempted to replicate ScatterLab’s optimized recipes (from experiments #41 and #56) using manual, human-operated synthesis methods. As detailed in section I of the Supporting Information, these trials resulted in substantial AuNP agglomeration, which is unsurprising given the high precursor concentration (∼3.5 mM). The discrepancy likely reflects practical differences between robotic and manual human-operated syntheses, including (i) addition rates that cannot be matched accurately by hand, (ii) difference in mixing behavior, and (iii) distinct illumination setups (e.g., lamp distances, container geometries, and photobox designs). Minor variations in reagents may also contribute. As summarized in Table S6, only the reagent–volume parameters are directly transferable, whereas mixing, light illumination, and gold-precursor addition speed are not, as they depend on hardware-specific conditions. Together, these factors highlight the inherent difficulty of translating a robotic flow-based synthesis protocol into a manual procedure. Despite this, the chemist drew inspiration from ScatterLab’s optimized recipes and performed six additional experiments, systematically excluding certain reagents (glycerol, NaCt, and NaOH). Although these altered protocols do not necessarily reproduce the same atomic structures as ScatterLab, two produced stable AuNP suspensions at ∼3.5 mM, confirmed by UV–Vis and TEM measurements 24 h postsynthesis. These results illustrate that while a one-to-one mapping from automated to manual synthesis is unlikely, the parameter regime identified by ScatterLab can nevertheless provide a productive starting point for human-led optimization; reducing the experimental search space and accelerating the development of new AuNP protocols.

## Conclusions

TS/PDF analysis provides general, atomic-level structure characterization, making it an appealing technique for structural feedback in SDLs across materials classes.^24–38^ We have demonstrated a proof-of-concept SDL, ScatterLab, that uses TS/PDF scattering patterns to autonomously guide AuNP synthesis. By matching experimental TS and PDF data to user-defined target patterns, ScatterLab learns synthesis protocols that yield materials whose scattering patterns closely match simulated patterns of an intended atomic arrangement.

The two targeted AuNP structures, ∼5 nm decahedral and 10 nm spherical FCC, were selected not for functional superiority, but to show that atomic-level structural targets defined solely through their scattering patterns can be approached autonomously. Over the course of the four-day beamtime, ScatterLab identified synthesis conditions that steadily progressed toward the simulated target scattering patterns. For the decahedral case, the optimization reached its most favorable region of the search space within 41 experiments and, leveraging information from this first campaign, required only nine additional runs to yield an FCC-like scattering response corresponding to larger AuNPs (∼7.5 nm). Interestingly, it autonomously minimized byproducts and can reach a AuNP concentrations of 3.5 mM—exceeding the typical 1 mM limit^10^—leading to more commercially viable end-products. Under identical conditions, repeated synthesis produced consistent scattering profiles, and the resulting protocols can be used for downstream synthesis at rates of ∼690 mL (∼0.5 g) per day per setup. However, the 5 min illumination cap means the reaction may not reach full completion, and long-term stability cannot be guaranteed. Although ScatterLab does not incorporate a priori chemical knowledge like mechanistic or synthesis heuristics, the optimization necessarily operates within a human-defined experimental domain (Table 1). These practical constraints accelerate optimization but also restrict the accessible chemistry. As SDLs mature, expanding these boundaries will allow exploration of broader reaction spaces, bringing the field closer to genuinely open-ended, discovery-driven autonomy.

**Table 1:** Parameter Bounds for Synthesis Conditions

	lower boundary	higher boundary	equivalent of high boundary in v.% or mM	rational of bounds
H_2_O (mL)	0	4.50	90 v %	90 v.% to ensure minimum two chemicals are used
NaOH (mL)	0	3.75	37.5 mM	37.5 mM is sufficiently to induce synthesis with 5 mM HAuCl_4_ conc. assuming NaOH/Au molar ratio of 4 leads to AuNPs^53^
ethanol (mL)	0	3.75	70 v %	<70 v.% ethanol is optimal for the synthesis^54^
NaCt (mL)	0	3.75	150 mM	A large maximum concentration was used for NaCt
glycerol (mL)	0	4.5	54 v %	max. 54 v.% glycerol to ensure that the solutions are not too viscous
HAuCl_4_ (mL)	0	1.25	5 mM	maintaining HAuCl_4_ below 5 mM helps preventing unwanted deposition of metallic gold on internal components
addition of Au speed (%)	20	60	-	excessively low motor speeds can lead to inaccurate pump speeds, while excessively high speeds risk mechanical crashes
mixing speed (% of max. power)	50	100	-	excessively low mixing speeds can lead to inaccuracy
UV–B (%)	0	50	-	excessive lamp intensity risks overheating the sample and potentially damaging the equipment over prolonged illumination
UV–C (%)	0	50	-	
White (%)	0	50	-	

We compare ScatterLab with the SDL performance-metric framework of Volk & Abolhasani (section J, Supporting Information).^55^ Unlike other high-throughput but specialized robotic setups,^56–60^ our robotic design, MODEX, emphasizes modularity, adaptability, and affordability (∼€2750). This design philosophy “democratises” autonomous experimentation,^61,62^ making it accessible to smaller research groups and compatible with constrained temporal environments such as synchrotron beamlines. Our full deployment was completed within four days of allocated beamtime. A full SDL cycle currently averages 17 min (section K, Supporting Information), with expected reductions of ∼50% through synthesis parallelization and workflow orchestration.

While TS/PDF provides substantially higher structural fidelity than UV–Vis spectroscopy, integrating multimodal data streams (e.g., SAXS, TEM, or optical spectroscopy) would further reduce structurally degenerate cases where multiple arrangements yield similar scattering patterns. Looking ahead, TS/PDF patterns simulated from structures discovered using ML-learned interatomic potentials could serve as user-defined targets, allowing ScatterLab to experimentally realize computationally predicted structures. By providing atomic-level insights across diverse chemistries, ScatterLab accelerates the synthesis of next-generation materials and lays the foundation for synthetically realizing computationally discovered structures, such as advanced catalysts, quantum dots, and other function-defining nanomaterials at timescales unreachable by conventional trial-and-error.

## Methods

### Overview of ScatterLab

ScatterLab (Figure 1) integrates four key components: (1) a secure communication framework between the database infrastructure, operational beamline control, and HPC cluster; (2) a small, modular robotic synthesis system, MODEX, that executes the prescribed synthesis protocol; (3) the DanMAX beamline at the MAX IV synchrotron for TS experiments and PDF measurements; and (4) an HPC cluster for BO proposals.

In an ScatterLab cycle, the BO algorithm, running on the HPC cluster, proposes a synthesis protocol, which MODEX executes to synthesize NPs under defined conditions. The synthesized samples are then transferred to the DanMAX beamline, where TS measurements are collected. These measurements are integrated, processed, transformed to PDF data and both TS and PDF data are compared to target data sets to compute an objective value reflecting how closely the scattering pattern of the synthesized material matches a presimulated scattering pattern of the target material. Based on these results, the HPC cluster refines the next set of synthesis parameters via BO, iteratively improving the outcome in real-time. Throughout this cycle, all experimental conditions and results are recorded in a MongoDB database.

### Orchestration Architecture

With the raise of smarter algorithms, autonomous synchrotron-based experiments become possible with the incorporation of closed-loop workflow and the feedback from online data processing.^19,20^ However, for security and operational stability, the beamline control system must remain isolated from user-developed code that executes on external servers. In our workflow, this separation manifests as two distinct environments: a local beamline control system, based on TANGO controls,^63^ and a remote HPC node equipped with a dedicated graphical processing unit (GPU) (NVIDIA V100) for BO routines. This split design brings multiple benefits. First, the beamline control software remains secure and stable for subsequent users, as new dependencies introduced by a single experiment do not risk disrupting essential beamline operations. Second, the HPC environment can be configured independently to accommodate the specialized software dependencies and large computational loads (e.g., GPU-accelerated data processing).

In our specific implementation, we reserve a GPU-enabled compute node on the HPC cluster for the duration of the beamtime, thereby minimizing the necessary computational time for running the BO routines. However, the HPC still need access to the beamline control system to pass the next measurement instructions. In the usual synchrotron experiment, the user-code is sand-boxed inside the beamline experiment control environment (i.e., Macro). For this unique experiment, a unidirectional message passing protocol from the HPC to the Tango control system has been developed to divide the beamline control from the BO routines in respect of the MAX IV security aspect. The raw scattering data is streamed from the detector to storage and azimuthal integration. Once a measurement is complete, a trigger signal prompt the HPC node to resume the ScatterLab cycle from the postprocessing step. Through these periodic exchanges, the beamline remains logically separate from the HPC environment, yet it can still exploit powerful offsite resources for computationally expensive tasks.

### MODEX: The Small, Modular Robotic Synthesis System

A key challenge was setting up an SDL at the synchrotron as fast as possible, which demanded a transportable, low-footprint design that could be assembled and calibrated rapidly. To meet these requirements, we developed a modular robotic synthesis system (Figure 5), in which each decimeter-scale module performs a single chemical unit operation (e.g., dosing, stirring, UV illumination) and interconnects via standard connectors. This approach allowed us to program and test the entire system beforehand, disassemble it for transport, and reassemble and calibrate it on-site at the synchrotron within just a few hours. Each module comprises a dry side for electronics and a wet side for chemical handling. They operate autonomously with integrated control units and communicate with the HPC cluster and MongoDB database via WiFi. In this study, we used four main modules.

- Syringe module (dimensions: W: 30 × L: 15 × H: 40 cm; mass: 4.7 kg, estimated price: €1753): Equipped with four syringe pumps (precision: 0.52 % and accuracy: 0.48% at full stroke) for dosing NaOH, NaCt, glycerol, and HAuCl_4_ precursor solutions.

**Figure 5:**
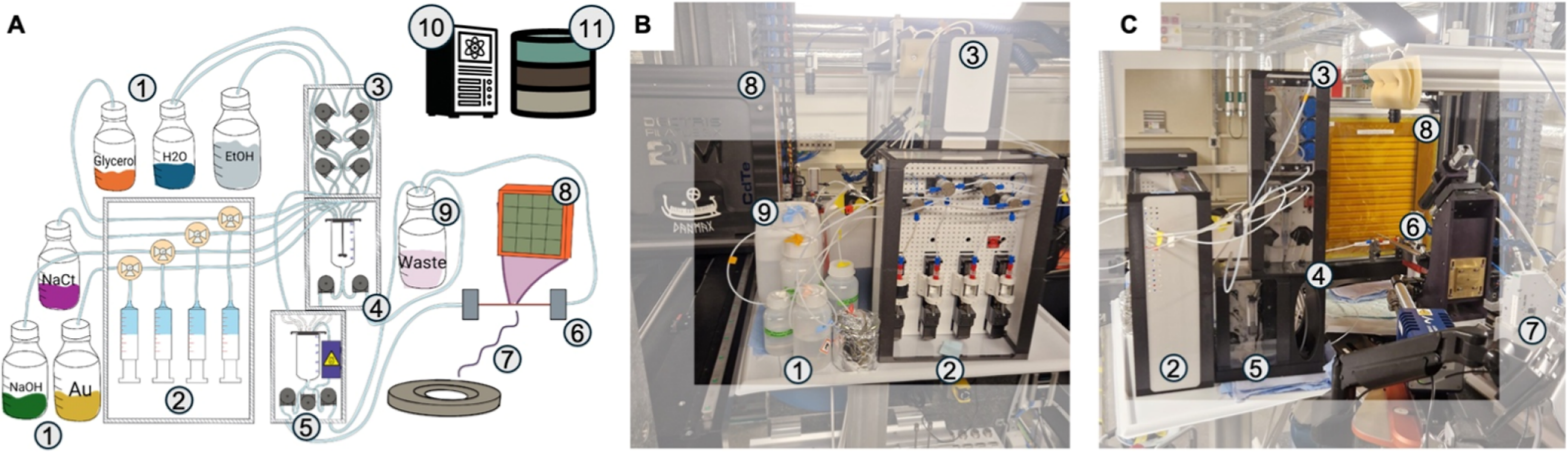
MODEX: MODular EXperimentation platform. (A) Schematic diagram of MODEX deployed at the synchrotron. Chemicals (1) are dispensed either with high accuracy via the syringe module (2) or with lower accuracy (but at reduced cost) via the pumping module (3), and are then delivered to the mixer (4) where they are homogenized. The mixture subsequently undergoes 5 min white/UV LED illumination (5) before being transferred to the capillary (6) for measurement. Here, the sample is irradiated with X-rays from the synchrotron (7), and the resulting scattering is recorded by the detector (8). Finally, the sample is directed to a waste container (9). The MODEX platform is controlled from a HPC computer (10), and all data is logged live into a cloud database (11) (B,C) Photographs of the system as deployed at the synchrotron, illustrating its integrated design and operational configuration.

- Pumping module (dimensions: W: 15 × L: 15 × H: 20 cm; mass: 1.5 kg, estimated price: €176):

Uses peristaltic pumps for less precise liquid dosing (H_2_O, EtOH). Precision remains below 1% relative error for volumes >1 mL, but diminishes at smaller volumes.

- Mixing module (dimensions: W: 15 × L: 15 × H: 20 cm; mass: 1.0 kg, estimated price: €267):

Homogenize chemical mixtures by stirring with a glass spatula (50–100% max. Power; ∼ 215–466 RPM in water).

- White/UV LED illumination module (dimensions: W: 15 × L: 15 × H: 20 cm; mass: 1.4 kg, estimated price: €551):

Incorporates a white LUXEON Rebel ES LED lamp (Lumileds, 5650K), a UV–B lamp (Luminus, 385 nm, SST-10-UV-B130-G385-00), a UV–C lamp (Klaran, 260–270 nm, KL265-50W-SM-WD), and a QF97 flow cell (10 mm path length, 3.5 mL volume, OD 4 mm, ID 2 mm, fused quartz), allowing photoillumination and direct transfer of the suspension to either waste or the beamline’s X-ray path.

Chemicals (in plastic bottles) are connected to the syringe and pumping modules, and the product stream can be diverted either to a waste container or through a 0.85 mm outer diameter fused silica tube (connected via a Sleeve Sealtight 1.05 mm and a Union, PEEK, 1/16″ 0.50 mm with F300) aligned with the X-ray beam. This design surmounts the logistical challenges posed by real-time synchrotron experiments, enabling rapid deployment and modular reconfiguration without compromising chemical handling or data quality.

### Synthesis Strategy

All chemicals were used as received: high purity water (mQ, Milli-Q, resistivity ≥18.2 MΩ·cm); HAuCl_4_·3H_2_O (≥99.9%, MP, Merck, 520918); sodium hydroxide (NaOH, ≥98%, pellets, reagent grade, Sigma-Aldrich); NaCt (≥99%, Sigma-Aldrich, BioUltra); glycerol (≥99.5%, Merck, g7893); ethanol (anhydrous, 99.9%, KiiltoClean).

The synthesis of AuNPs proceeded by mixing up to six stock solutions (all prepared in mQ water) under BO-controlled conditions, followed by a five-minute illumination period using white, UV–B, and/or UV–C LED sources. This illumination duration was selected based on literature precedent^53,64^ and experimental validation (section L, Supporting Information), representing an optimal balance between reaction completeness and efficient use of experimental time. After each sample synthesis (section M, Supporting Information), the entire system was washed with Milli-Q water.

The gold precursor was dissolved to yield a 20 mM solution. Sodium hydroxide was prepared as a 50 mM aqueous solution, while NaCt was prepared as a 200 mM aqueous solution. Glycerol was diluted to 60 v % in water. Ethanol was used as received. Milli-Q water was also used as stock solution and to prepare all solutions.

The search space for synthesis parameters was defined by the lower and upper boundaries listed in Table 1, which include volumes of the stock solutions of the various chemicals, addition speeds for the Au precursor, mixing speeds, and illumination (white, UV–B, and UV–C) LED intensities. These bounds ensured that the optimization process could explore a wide range of conditions while maintaining sensible chemical constraints. In principle, the parameter bounds could encompass conditions used for synthesizing atomically precise gold nanoclusters.^65,66^ In practice, however, these systems are produced as strongly ligand-stabilized, dilute solutions that fall below the practical concentration range required for one-minute TS/PDF measurements in the liquid phase.

### Moving the Synthesized Product to the Beam

Once a sample is synthesized, it is moved from MODEX to the X-ray beam by the pumping module’s peristaltic pump using a controlled flow through the connecting tube. Initially, the tube contains air, and a reference air scattering pattern is collected beforehand. To determine when the liquid sample reaches the measurement capillary, short (one-second) scattering measurements are continuously acquired as the pump slowly moves the fluid. After each measurement, the MSE between the experimental scattering pattern, *I*_exp_(Q), and the known air scattering pattern, *I*_air_(Q) is calculated. If this value remains below a predefined threshold of 10^–3^ (section N, Supporting Information), the measurement is considered to represent air, and the system continues pumping. If the value exceeds 10^–3^ once, it may indicate a liquid droplet or the sample itself. To confirm that the actual sample, rather than just a droplet, has arrived, two consecutive measurements must exceed the threshold. Upon this confirmation, the pump is stopped, and a longer, one-minute measurement is performed to characterize the sample.

### TS Experiments with PDF Analysis

X-ray TS experiments were performed at the DanMAX beamline at the MAX IV Laboratory using a Si (1 1 1) monochromator beam with an energy of 35.00 keV (λ = 0.354 Å). The incident beam was focused to approximately 0.814 mm (H) × 0.722 mm (V) fwhm at the sample position. Scattered intensities were recorded using a DECTRIS PILATUS3 × 2 M CdTe detector placed 149.9 mm downstream of the sample. The sample-to-detector distance was determined based on measurements on crystalline Si (NIST SRM 640f). Each measurement, for both sample and blank, was collected for 1 min in a fused silica capillary with an 0.7 mm inner diameter. Here, “blank” measurements—performed without the metal precursor—were used for blank/background subtraction, while “sample” measurements—collected with the metal precursor—provided the actual NP scattering signal.

After each measurement, the two-dimensional detector images were azimuthally integrated using the MATFRAIA algorithm.^67^ The resulting one-dimensional TS data were subsequently processed with PDFgetX3 ^68^ yielding *I*(Q), *S*(Q), *F*(Q), and *G*(r) data. An example of scattering patterns from the sample, the blank, and the blank subtracted scattering pattern are shown in section O in the Supporting Information.

PDFGetX3 determines the coherent intensity, *I*_*coh*_, using an ad hoc approach that exploits the higher-frequency scattering above $∼\frac{2\pi }{r_{poly}}$ , where *r*_poly_ is the nearest-neighbor distance. It then calculates the S­(Q) as follows^23^

1 $$S\left(Q\right)=\frac{I_{coh}\left(Q\right)+{\left\langle f\left(Q\right)\right\rangle }^{2}-\left\langle {f\left(Q\right)}^{2}\right\rangle }{N{\left\langle f\left(Q\right)\right\rangle }^{2}}$$

where S­(Q) normalizes the coherent elastic scattering intensity, *I*_coh_, using the average scattering power, ⟨*f*(Q)⟩. The term ⟨*f*(Q)⟩^2^-⟨*f*(Q)^2^⟩ accounts for imperfect cancellation between scattering by different elements.^23^ The next mathematical treatment of the data is to calculate the *F*(*Q*), where the high *Q* regime is enhanced^23^

2 F(Q)=Q(S(Q)−1)

Fourier transforming *F*(Q) from *Q*_min_ to *Q*_max_ yields the *G*(r), also referred to as the PDF^23^

3 $$G\left(r\right)=\frac{2}{\pi }\int_{Q_{\min}}^{Q_{\max}}F\left(Q\right)\sin\left(Qr\right)dQ$$

Because the finite *Q*_range_ (*Q*_min_ – *Q*_max_) introduces mathematical artifacts known as “termination ripples”, we collected data over a large *Q*_range_ using a rapid-acquisition PDF^69^ setup, positioning a 2D detector close to the sample and using a relatively high energy to reach high *Q*-values in short acquisition times. For this study, we used *Q*_min_ = 0.5 Å^–1^, *Q*_maxinst_ = 18.5 Å^–1^, and *Q*_max_ = 15 Å^–1^, with *r*_poly_ = 0.9 Å. Note that *Q*_maxinst_ is applied when removing coherent scattering components, while *Q*_max_ is used in the final Fourier transform to produce *G*(r).

### Objective Function

The objective value quantifies the discrepancy between the experimentally measured scattering patterns of the synthesized NPs and their targeted scattering patterns. The objective function is defined as the MSE between the experimental and target F­(*Q*) and G­(*r*). Specifically, the objective value is given by

4 $${L=MSE}_{Fq}+{MSE}_{Gr}=\frac{1}{n}\sum_{i=1}^{n}{\left({F\left(Q\right)}^{\exp}-{F\left(Q\right)}^{target}\right)}^{2}+{\left({G\left(r\right)}^{\exp}-{G\left(r\right)}^{target}\right)}^{2}$$

As L approaches zero, the experimental scattering patterns more closely resemble the target patterns. Minimising L thus guides the optimization process toward the desired scattering pattern and consequently material structure.

### BO Algorithm

BO was employed to efficiently minimize the objective value described above, addressing the complex and costly nature of synthesis experiments. BO is well-established in optimizing expensive black-box functions across diverse scientific domains, including chemistry and materials science.^12,70^ It uses previously obtained data {( $x_{n}$ , $f\left(x_{n}\right))$ }, where $x_{n}$ represents a set of synthesis parameters and $f\left(x_{n}\right)$ the corresponding loss (i.e., discrepancy with the simulated patterns) to build a probabilistic model of the objective function. This model, combined with an acquisition function that prioritises regions of high potential improvement, facilitates a systematic search for optimal conditions while balancing exploration and exploitation.

Random forests and Gaussian processes (GPs) have recently been benchmarked across multiple experimental materials science domains, concluding that GP with anisotropic kernel is more robust across design spaces.^71^ In our study, a GP regression model^72^ was selected to approximate the objective function, capturing both the predictive mean and uncertainty in unexplored regions of parameter space. In GP regression, the observations [ $f\left(x_{1}\right)$ , ..., $f\left(x_{n}\right)$ ] are assumed to follow a joint normally distribution determined by a mean function μ and a kernel function *k*. This assumption allows us to predict values at unseen points $f\left(x_{*}\right)$ by conditioning on previous information, arriving at the following posterior distribution $f\left(x_{*}\right)$ . An acquisition function derived from the GP’s predictive distribution then identifies promising new sets of parameters that either exploit known high-performing areas or explore under-sampled domains. This sequential approach enables a targeted and data-driven strategy for converging on optimal synthesis conditions. To further validate correlations identified by our BO algorithm, cross-validation analyses of the GP surrogate model demonstrate its effectiveness in learning meaningful chemical trends from experimental data (section P, Supporting Information), and potentially, with more data, be used as a surrogate for the experiment itself.

BO is known for scaling poorly with the dimensionality of the input space.^73–75^ In our specific scenario, the size of the parameter space ( R ^11^) invites us to consider high-dimensional alternatives for BO. Two contemporary methods for competitive high-dimensional BO algorithms are Hvarfner’s D-scaled *p*(*l*) (VanillaBO^76^) and SAASBO^41,74^ SAASBO includes a sparsity-promoting prior, but remains robust in moderate-dimensional settings where several parameters may be correlated. Our in silico benchmarking (section E, Supporting Information) indicates that correlations or nonsparse effects did not hinder optimization in practice. Vanilla BO, which does not assume axis-aligned relevance, performed comparably but was slightly less sample-efficient. Based on these benchmark results, we selected SAASBO with an noisy log-Expected Improvement acquisition function^77^ and a Matern 5/2 kernel^78^ using a Bayesian approach to kernel hyperparameters (default settings in Ax, version 0.4.1.). If stronger nonsparsity were expected, SAASBO’s shrinkage hyperparameter could be relaxed accordingly.

For initial exploration, we generated a random set of experiments to ensure broad coverage of the parameter space. Following advice from other practitioners in the field, we started with 2·*d*+2 initial random samples, where *d* is the number of parameters. To achieve uniform coverage, we used SOBOL sampling for all variables. For parameters representing chemical volume proportions (which must sum to unity), we applied a normalization procedure after drawing from a uniform distribution within defined bounds (Methods, “Synthesis Strategy”). Once this initial data set was collected, BO was initiated to iteratively refine synthesis parameters. Over successive iterations, the chosen BO method proposed new parameters predicted to minimize the objective value, steadily steering the synthesis conditions to yield experimental scattering patterns closer to the target scattering patterns. All BO routines were implemented using Ax, BoTorch, and GPyTorch.^79–81^ In practice, BO proposals are generated on a single NVIDIA V100 GPU, with a typical wall time of ∼2 min per iteration. Assuming a cloud price of $3 per GPU hour, the marginal compute cost is $0.10 per experiment, which is negligible compared with reagent and synchrotron beamtime expenses. Input validation was managed with Pydantic,^82^ which allowed us to communicate the proposed parameters with the robot seamlessly.

### Real-Time Data Storage

All experimental and operational data generated by ScatterLab were stored in a MongoDB database, which facilitated centralized data management and real-time accessibility. The database is organized into three collections: (1) a robotic collection documenting detailed logs of every MODEX module action, including the module’s IP address, the timestamps and date of operation, the assigned descriptive label, and the exact sequence of commands issued (such as “pump for 242.2 ms with a speed of 0.6 mL/s”); (2) a scattering collection containing the integrated and processed scattering data (I­(*Q*), S­(*Q*), F­(*Q*), and G­(*r*)), as well as its corresponding scan number and timestamp; and (3) a BO collection that recorded the synthesis parameters, their corresponding objective values, experiment statuses (“failed” or “completed”), and labels (“random” or “BO”). Both completed and failed records are logged for completeness and remained accessible for subsequent review or modification by a human operator, enabling retrospective decisions. However, only completed experiments contributes to training BO algorithm, ensuring that parameter selections for subsequent synthesis runs are based solely on valid and reliable data.

### Monitoring, Intervention, and Path to Full Autonomy

ScatterLab is designed to autonomously conduct NP synthesis, carry out data measurements and processing, execute BO proposals, and store all information in a central database. Under standard laboratory conditions, the workflow performed smoothly. However, conducting experiments at a synchrotron poses additional challenges (section A, Supporting Information)—including limited beamline access, short experimental windows, stringent safety protocols, and the challenge of integrating a robotic system into existing beamline infrastructure. During our four-day beamtime, five experimentalists, three of whom had never worked at a synchrotron before, monitored ScatterLab. Despite these demanding conditions, ScatterLab demonstrated its capacity and robustness to carry out the intended tasks.

Nonetheless, ScatterLab still requires occasional human intervention. Transient issues such as a pump failing to respond (e.g., due to queue errors or momentary WiFi disruptions) may arise. In addition, sample measurement occasionally starts with bubbles in the capillary or when the sample is misaligned with the X-ray beam. As SDL-based experimentation becomes more mainstream, we expect these technical details to be refined through iterative improvements in both hardware and software. To address these challenges, an operator continuously monitors ScatterLab via live data visualizations—plotting scattering data, objective values, and BO predictions for both training and test sets—and via camera feeds of each robotic module (section Q, Supporting Information). Critically, the operator does not need to be highly specialized; they primarily watch for disruptions that might halt operation. In most cases, an error simply pauses ScatterLab, enabling a manual restart from the last successful step. If the disruption renders a particular experiment invalid—such as a measurement taken with a bubble—its status is marked as ‘failed’ within a 10 s window, ensuring that inaccurate data points do not corrupt subsequent optimization. Over the four-day beamtime, more persistent issues also emerged. For example, the mixing module failed after experiment #26 and prolonged use caused gradual deposits on the light cuvette. While these setbacks may introduce drift in the experimental conditions—suboptimal for the BO algorithm—they did not compromise ScatterLab‘s underlying autonomy or methodology. Contrary, they highlight opportunities to reduce error frequency and improve the ratio of completed to ‘failed’ experiments, ultimately accelerating each SDL cycle.

### Scattering Data Modeling

To quantitatively analyze the experimental scattering data, we use two distinct modeling approaches depending on NP size. Attenuated crystal approximation is used for larger, more crystalline particles, while finite-cluster methods—specifically the Debye scattering equation—is applied to smaller nanoclusters.

### Finite clusters

When NPs are small or exhibit significant surface and finite-size effects, the conventional unit-cell-based approaches become unsuitable. Instead, one must compute the TS contribution from every atomic pair. Our goal is to identify which structural motifs—octahedral, icosahedral, decahedral, or FCC—best represent the synthesized AuNPs, as well as to estimate their characteristic sizes.

Inspired by the Cluster-mining approach introduced by Banerjee et al.,^49^ we construct a comprehensive library of model structures using the Atomic Simulation Environment (ASE).^83^ Each structure is generated with a fixed lattice constant of 4.07 Å—matching our experimental data set— and an atomic composition of pure gold. The FCC-type clusters are assumed to be spherical, whereas the cluster-type geometries (octahedral, icosahedral, decahedral) are generated by specifying various truncation criteria and layer counts along high-symmetry directions. For example, regular octahedra is defined by exposing only {111} facets, and icosahedra^84^ were built by stacking successive triangular facets around a closed shell. Decahedra are created by adjusting parameters such as the number of layers parallel to the pentagonal axis and the extent of truncation at the pentagonal edges or apical vertices, thus yielding a family of shapes ranging from regular decahedra to Ino- and Marks-type decahedra.^85,86^

By systematically varying these structural parameters, we obtain a diverse set of candidate geometries that capture differences in atomic stacking, twin boundary formation, and exposed facets. This structural library enabled a thorough comparison between simulated and experimental scattering patterns, allowing us to pinpoint which morphology (and approximate size) best describes the AuNPs produced by ScatterLab.

Because the NPs are finite rather than periodic solids, their scattering patterns are computed using the Debye scattering equation^87,88^

5 $$I\left(Q\right)=\sum_{\nu =1}^{N}\sum_{\mu =1}^{N}f_{\nu }\left(Q\right)f_{\mu }\left(Q\right)\frac{\sin\left(Qr_{\nu \mu }\right)}{Qr_{\nu \mu }}$$

where *Q* is the magnitude of the scattering vector, *N* is the total number of atoms, and *r*_νμ_ is the interatomic distance between atoms ν and μ. *f*(*Q*) represents the atomic form factors. The computational cost of this approach can be substantial, scaling as *O*(*N* ^2^). Since some structures contain as many as 14993 atoms, we use the DebyeCalculator^44^ software (v.1.0.14) with GPU acceleration to handle the 1965 candidate models efficiently.

All fits are done on combined F­(*Q*) and G­(*r*) data covering a *Q*-range of 1.2–15 Å^–1^ and an *r*-range of 2–30 Å. We use a *Q*_damp_ value of 0.0274 Å^–1^ to represent instrumental broadening, determined from a crystalline Si standard (NIST SRM 640f) measured under identical conditions. We fit the scaling of the F­(*Q*) and G­(*r*) data, and ADPs to account for thermal motion. All fitted patterns are compared directly against the experimental data to calculate *R*_wp_ values, thereby indicating which structural motif and size most accurately described the synthesized AuNPs.

### Attenuated crystal Approximation

For larger, more crystalline AuNPs—where finite-size effects are less pronounced—we employ a conventional attenuated crystal approximation, implemented through TOPAS^89^ (v.7) for combined Rietveld and real-space Rietveld refinement. This method is particularly effective for well-ordered, larger NPs, complementing the Debye-based approach used for smaller clusters. We perform refinements on I­(*Q*) over the 3.7–11.2 Å^–1^ range and on G­(*r*) from 2–30 Å. We excluded the first two Bragg peaks in the Rietveld refinement as there was still some background signal left, which made it difficult to fit the peak intensities. The combined Rietveld and real-space Rietveld refinements were performed by refining the structural parameters of the Au FCC phase simultaneously to both data sets. We refined the cubic unit cell parameter, the isotropic ADP in B, and a spherical crystallite diameter. All three parameters were fixed to be the same for both data sets. For the reciprocal-space Rietveld refinement, the peak shape was described using a Thompson-Cox-Hasting pseudo-Voight with the size broadening described using the Voight_Integral_Breadth_GL macro as described by Dinnebier et al.^90^ The instrumental contribution to the peak shape was obtained from a Si standard (NIST SRM 640f) and kept fixed during the refinement. For the PDF refinement, the size dampening was implemented using the spherical_damping macro written by Phil Chater.^91^ The same Si standard was used to obtain the instrumental dampening of the PDF using the dQ_dampening macro in TOPAS. This parameter was also kept fixed during the refinement. In the reciprocal space refinement, zero error and a 10th-degree Chebyshev polynomial background function were refined. For the real-space refinements, the peak shape was described using the PDFgui^92^ peak shape model with *Q*_damp_ = 0.0274 Å^–1^ obtained from the Si reference and δ_2_ fixed as 2 Å^–1^. To avoid issues with scaling the data sets, the highest peak was normalized to 1 for both data sets, and each data set also had an individual scale factor refined.

## Supplementary Material



## Data Availability

Code and data for the experiments described in this manuscript is 941 available at: https://github.com/AndySAnker/scatterlab-2026-tspdf-target-scattering and https://zenodo.org/records/18427148.

## Notes

The experimental design, benchmarking, and validation of ScatterLab. It includes discussions of practical challenges associated with synchrotron-based SDL experiments; comparisons to alternative AuNP synthesis and optimization strategies; in silico benchmarking of BO hyperparameter choices; repeatability tests; details of the scattering data modeling; illustrations of the used synthesis parameters; manual control experiments; SDL performance metrics; time profiling; and auxiliary studies on illumination, washing, background subtraction, and analysis of the BO framework. Supplementary figures, tables, photographs from the beamtime, and extended methodological descriptions are also included (PDF)
