# Supplementary material for: Autonomous Synthesis of Nanoparticles with Target Scattering Patterns
Source: ACS Nano. 2026 Feb 18;20(8):6767–82. doi: 10.1021/acsnano.5c15488 (PMC12961950; doi:10.1021/acsnano.5c15488)
Supplement: Supplementary file 1 [file nn5c15488_si_001.pdf]

Autonomous Synthesis of Nanoparticles with Target Scattering Patterns

Andy S. Anker[*1,2], *Jonas H. Jensen*[3], *Miguel González-Duque*[4], *Rodrigo Moreno*[3], *Aleksandra Smolska*[5], *Mikkel Juelsholt*[6], *Vincent Hardion*[7], *Mads R. V. Jørgensen*[7,8], *Andrés Faíña*[3], *Jonathan Quinson*[5], *Kasper Støy*[3], *Tejs Vegge*[1]

*Correspondence to ansoan@dtu.dk (ASA)

1: Department of Energy Conversion and Storage, Technical University of Denmark, Kgs. Lyngby 2800, Denmark

2: Department of Chemistry, University of Oxford, Oxford OX1 3TA, United Kingdom

3: Department of Computer Science, IT University of Copenhagen, 2300 Copenhagen, Denmark

4: Department of Biology, University of Copenhagen, Copenhagen 2200, Denmark

5: Biological and Chemical Engineering Department, Aarhus University, Aarhus 8200, Denmark

6: Department of Chemical Engineering, Columbia University, New York, NY 10027, USA

7: MAX IV Laboratory, Lund University, Lund 225 94, Sweden

8: Department of Chemistry and iNANO, Aarhus University, Aarhus C 8000, Denmark

Table of Contents

A: Challenges of conducting self-driving laboratory (SDL) experiments at synchrotrons 4

B: Comparative approaches to optimisation of AuNP synthesis protocols 5

Manual syntheses 6

Brute-force high-throughput experimentation 6

Autonomous laboratories 6

C: Comparison of autonomous laboratories reported for the optimisation of AuNP synthesis 7

D: Overview of AuNP synthesis strategies 8

Control over AuNP syntheses 8

Selection of synthesis strategy 9

How does ScatterLab advance AuNP synthesis 10

E: *In silico* benchmarking: Optimising hyperparameters of ScatterLab 10

BO algorithm 12

Scattering function 14

Data normalisation 16

Objective function 19

Differences between benchmarking and synchrotron experiments 21

F: Repeating experiment #41 22

G: Modelling of the scattering data from experiment #41 25

H: Behind the scenes: how ScatterLab navigated AuNP synthesis variables 28

I: Manual, human-operated synthesis **31**

Long-term stability 34

Chemicals 34

Synthesis 34

UV–Vis 35

STEM 36

Transferable parameters between robotic and manual synthesis 36

J: Performance metrics for the SDL **36**

K: Time profiling of the individual operations in ScatterLab **38**

L: Influence of illumination duration with white and UV light **39**

M: Influence of washing step between blank and sample synthesis **41**

N: Determining whether a measurement is obtained on air or not **41**

O: Background subtraction of the scattering data **42**

P: Chemical insights from Gaussian Process (GP) surrogate modelling **43**

GP predictions and cross-validation 43

Feature importance analysis 45

GP as a surrogate for experimental exploration 46

Q: Photographs from the beamtime **49**

References **52**

A: Challenges of conducting self-driving laboratory (SDL) experiments at synchrotrons

1. **Limited beamline access**

   Obtaining synchrotron beamtime generally involves a peer-reviewed proposal process spanning several months. Even if approved, scheduling occurs at fixed intervals, reducing the flexibility required for iterative SDL improvements. Consequently, the SDL must perform reliably on its very first attempt.

2. **Tight experimental windows**

   Most beam allocations last only a few days—one week is considered generous. Installing and calibrating an SDL (robotic modules, data-processing pipelines, etc.) typically requires longer than the allotted time. Thus, a modular design and rapid deployment strategies therefore become essential for any SDL intending to exploit advanced synchrotron methods.

3. **Experimental station integration constraints**

   Incorporating a robotic synthesis platform at a beamline demands navigating limited spatial footprints, strict safety protocols, and compatibility with existing instrumentation. These constraints can reduce the effective number of experiments possible within a short SDL campaign. However, overcoming them with a fully automated sample exchange—interoperable with beamline hardware—can streamline operation and maximise the productivity of scarce beamtime resources.

4. **Advanced data analysis and expertise**

   Synchrotron data typically necessitate extensive post-processing (e.g. azimuthal integration, blank/background subtraction, pair distribution function (PDF) transformations), requiring specialised expertise. Multiple PhD-level operators are often needed to handle both synthesis and scattering analysis. This can become a bottleneck when aiming for real-time experiments with minimal human intervention. Instead, the SDL must automate sample preparation and data processing to decrease the reliance on skilled operators and boost throughput.

5. **Software orchestration challenges**

Integrating an SDL with beamline infrastructure also poses significant software hurdles. Synchrotron control environments typically use specialised software and security protocols, complicating direct communication with external high-performance clusters or robotic controllers. Although continuous data flow is crucial for real-time decision-making, issues like firewalls, non-standard file systems, and diverse software ecosystems can hamper interaction. A carefully designed software orchestration layer is therefore vital for robust, low-latency exchanges of commands and data, enabling uninterrupted synthesis–characterisation–feedback loops during limited beamtime.

6. **Safety considerations**

International research facilities uphold rigorous regulations on handling hazardous chemicals. Substances commonly used in gold nanoparticle (AuNP) syntheses, such as sodium borohydride ($NaBH_4$) or cetrimonium bromide (CTAB), may be restricted, especially if managed autonomously.

SDL frameworks have the power to significantly accelerate chemistry research, however, they must be compact, easily deployable, and capable of fully automated data processing to make the most of limited (and precious) experimental windows. Developing such modular SDL platforms—where synthesis, scattering measurements, and data analysis are seamlessly integrated—paves the way for accelerating materials discovery at large-scale facilities without necessitating an entire team of specialists for each experiment.

B: Comparative approaches to optimisation of AuNP synthesis protocols

Controlling NP size, shape, and structure typically involves several distinct strategies, here categorised into manual syntheses, high-throughput experimentation, and autonomous laboratories.

Manual syntheses

Manual synthesis is the conventional approach, involving human-driven experimentation where each synthesis step is executed manually. Structural parameters are subsequently characterised, typically through microscopy, spectroscopy, or scattering techniques informing adjustments for subsequent experiments. This iterative method heavily relies on empirical insights, serendipity, and educated guesses, often requiring extensive time and effort with relatively low throughput.

Brute-force high-throughput experimentation

High-throughput experimentation automates part or all of the synthesis process, in some cases but rarely the characterisation as well, significantly accelerating data generation. A robotic platform performs parallel or sequential syntheses, and humans characterise the resulting structures to inform subsequent experimental decisions. Although high-throughput experimentation improves throughput, the core strategy remains empirical, relying on faster identification of optimal conditions rather than inherently smarter experimentation (which is thus time and cost optimised).

Autonomous laboratories

Autonomous laboratories represent a recent advancement where both synthesis and characterisation are automated, and an algorithm determines the subsequent experiments to optimise specific size, shape, or structural outcomes. AuNPs have increasingly been studied in autonomous experimentation platforms, as summarised in Table S1+2.

ScatterLab uniquely distinguishes itself among autonomous laboratory approaches by directly optimising towards an atomic-scale structural target defined by scattering patterns, rather than indirect optical properties

such as UV-vis absorption. This direct targeting offers unprecedented control over NP size and atomic arrangement. Moreover, ScatterLab inherently optimises precursor concentration, achieving higher values than other optimisation approaches.

Strategy	Relative typical number of parameters screened in a study	Typical experimental space explored	Relative throughput	Size, shape, structure control by
Manual synthesis	Few (typically 2-3)	One parameter at a time	Low	Serendipity and/or educated guess
Brute-force high-throughput synthesis	Several	One parameter at a time	Medium	Accelerated serendipity and/or educated guess
Other Autonomous synthesis	Many	All parameters simultaneously	Medium / high	Indirect target (e.g., UV-vis) see Table S2
**ScatterLab synthesis**	Many (11)	All parameters simultaneously	Medium / high	Direct target (scattering pattern)

Table S1 | Relative comparison of ScatterLab to alternative NP synthesis strategies.

C: Comparison of autonomous laboratories reported for the optimisation of AuNP synthesis

Study	Synthesis strategy	Optimised concentration Au / mM	Experimental platform	In-line Characterisation
Nat. Commun. (2020)[1]	($NaBH_4$), CTAB, $AgNO_3$, AA, $HAuCl_4$ 30 °C (90 min / sample, batch = 15)	~0.5 mM	Robotic liquid handling	UV–Vis
Adv. Funct. Mat. (2021)[2]	PVP, Glucose, NaOH, $HAuCl_4$ 60 °C (2–10 min, on the fly)	~0.8 mM	Microfluidic	UV–Vis
Sci. Adv. (2022)[3]	($NaBH_4$), CTAB, CTAC, HQ, AA, $AgNO_3$, NaOH, $HAuCl_4$ 30 °C (2–16 hrs. / sample, batch = 24)	~0.5 mM	Robotic liquid handling	UV–Vis
Nat. Synth. (2023)[4]	($NaBH_4$), CTAB, $AgNO_3$, HCl, AA, $HAuCl_4$ 28 °C (12 hrs. / sample, batch = 96)	~0.7 mM	Robotic liquid handling	UV–Vis + colour sensitive camera
Nat. Commun. (2025)[5]	IN-2959, CTAB, $HAuCl_4$, $AgNO_3$ 27 °C (5+ min / sample, on the fly)	<0.15 mM	Microfluidic	UV–Vis-NIR
Npj Comp. Mat. (2025)[6]	($NaBH_4$), CTAB, $AgNO_3$, AA, $HAuCl_4$ 30 °C (90* min / sample, on the fly)	<1 mM*	Robotic liquid handling	UV–Vis

**ScatterLab**	Glycerol, NaCt, NaOH, EtOH, $HAuCl_4$<br>Room temperature, UV-induced<br>(5 min / sample, on the fly)	3.5 mM	Modular liquid robot	TS / PDF

Table S2 | Comparison of autonomous laboratories reported for the optimisation of AuNP synthesis. Each study is characterised by (i) choice of synthesis strategy, (ii) yielded AuNP concentration, (iii) experimental platform, and (iv) in-line characterisation approach. Abbreviations: CTAC = cetrimonium chloride, AA = ascorbic acid, PVP = polyvinylpyrrolidone, HQ = hydroquinone, IN-2959 = photo-reducing agent, NaCt = sodium citrate tribasic dihydrate. In references where "($NaBH_4$)" appears, seeds were pre-made using a separate reduction step. The * value is an estimate in the absence of supporting information in the preprint. Our approach (bottom row) achieves higher AuNP concentrations (3.5 mM) through a fundamentally different, faster, and safer synthesis strategy. It also provides atomic-scale information via total scattering (TS) and PDF analysis, rather than UV–Vis spectroscopy. Crucially, these experiments are enabled by our newly developed modular, compact robotic setup that easily integrates with other instrumentation.

D: Overview of AuNP synthesis strategies

Numerous syntheses of AuNP have been reported, extensively summarised in various reviews.[7, 8]

Control over AuNP syntheses

AuNPs can be obtained by multiple synthesis strategies and using a plethora of chemicals. Biological routes employing extracts from plants, fungi, bacteria, or viruses as reducing agents or stabilisers are common.[9] Conventional chemical syntheses frequently involves reducing agents and stabilisers such as citrate, thiols, $NaBH_4$, alcohols, ascorbic acid, CTAB, and many others, often in various combinations.[10-15] The selection of the nature and the relative ratio of these chemicals remain largely empirical, requiring significant trial-and-error experimentation for precise size, shape, or structure control.[16, 17] Advanced morphologies often require specific shape-directing agents and meticulously designed recipes, further increasing experimental complexity.[15, 18]

Selection of synthesis strategy

The chemicals used in AuNP synthesis directly influence NP size, shape, structure, formation pathways, and kinetics.[8] Consequently, comparing recipes across different studies is challenging. Table S3 provides an overview of some pros and cons of various AuNP synthesis strategies. In our approach, strict safety requirements at the synchrotron facility encouraged the use of safer chemicals. For instance, while $NaBH_4$ enables rapid synthesis (seconds), it is hazardous due to its strong reducing properties and flammability, and it furthermore has a short shelf-life of stock solutions. Similarly, seed-mediated methods, which often employ $NaBH_4$-generated NP seeds,[19] were incompatible with our time-constrained synchrotron beamtime due to the typical slower growth kinetics required to achieve specific structures.[19] Thus, our selected strategy relied on inherently safer and more sustainable reducing agents and stabilisers such as glycerol and citrate.

**Synthesis strategy**	**Typical chemicals***	**Method**	**Relative hazard****	**Typical number of chemicals**	**Typical time of synthesis**	**Control over*****	**Typical conc. of gold precursor**	**Relative time chronology**
Borowskaja Turkevich Frens (Citrate mediated)	Citrate (Water)	High T UV/Light induced	Low - medium	3	Minutes	Size Shape (Structure)	<1 mM	1934[20], 1951[10, 21]
Brust Schiffrin ($NaBH_4$ mediated)	$NaBH_4$ Thiols (Toluene)	Room T induced	High	2-4	Seconds Minutes	Size Shape (Structure)	<1 mM	1994[22, 23]
Seed mediated	Seeds (often $NaBH_4$-based) Growth agents	Range of T induced	Low - medium	4	Hours	Size Shape Structure	<1 mM	From 1950s[19]
Alcohol mediated	Alcohols (glycerol, ethanol) NaOH (Water)	Low/Room T UV/Light induced	Low	4	Minutes	Size (mainly)	<1 mM	2019[19] 2023[13]
**ScatterLab**	Alcohols NaOH Citrate (Water)	UV/Light induced	Low	6	Minutes	Structure Size	3.5 mM	2025

Table S3 | Relative comparative overview of AuNP synthesis strategies. As highlighted in the '*Long-term stability*' section, the long-term stability of the as prepared AuNPs can be limited to weeks but dilution ensures longer term stability.

* List is non exhaustive, excluding gold precursor. T stands for 'temperature'.

** Taking into account the nature of the chemicals, e.g. typically CTAB and/or $NaBH_4$ (harmful) are used, and/or the need for high(er) energy.

*** Shape and structure are linked but often the incentive is on optimising the shape, not directly the structure.

<u>How does ScatterLab advance AuNP synthesis</u>

Although the synthesis strategy themselves, summarised in Table S3, are not novel *per se*, their optimal combination (synthesis protocol) efficiently identified by ScatterLab (from a 11-dimensional synthesis space), would not be readily apparent through manual or high-throughput experimentation. This is particularly significant given ScatterLab's ability to achieve notably high AuNP concentrations (~3.5 mM) compared to typical reported values (<1 mM) while also having control of the atomic structure and size.

E: *In silico* benchmarking: Optimising hyperparameters of ScatterLab

Allocating only four days at the synchrotron (plus one day without the beam) to both set up and execute the experiment leaves no time to systematically benchmark various experimental and computational parameters on-site. These parameters, which can be viewed as hyperparameters, include: (1) the choice of Bayesian optimisation (BO) algorithm, (2) the selection of scattering data types (e.g., Q-space, r-space), (3) the normalisation strategy for the scattering data, and (4) the type of objective function.

To address these constraints, we conducted an *in silico* benchmarking campaign using a simulation framework (ScattBO[24]), which mimics the functionality of the SDL by taking proposed synthesis parameters as input and, through predefined rules, generating a virtual atomic structure and its corresponding simulated scattering pattern. Specifically, the predefined rules within ScattBO are as follows: The overall size of the generated atomic structures are correlated to the intensities of UV-A, UV-B lamps, and LED lamps, with higher illumination leading to larger structures.. Temperature is linearly mapped to the lattice constant (2.5–4.5 Å) as the temperature ranges from 20 to 70°C, thus modelling thermal expension commonly observed in chemical synthesis. The structure type selection depends primarily on the added chemicals and mixing speed: for larger structures (above approximately 2000 atoms), the predominant solvent component guides the structure type towards face-centred cubic (FCC), simple cubic, body-centred cubic, or hexagonal close-packed geometries. Conversely, smaller clusters adopt structure types such as icosahedral, decahedral, body-centred cubic, or octahedral shapes, determined by the mixing speed and specific pumping conditions—mirroring the transition from cluster to bulk behaviour commonly observed in both experimental and computational studies.[25-27] In the real experiment, the synthesis and measurements are instead fulfilled by the robotic synthesis system and the synchrotron beamline, respectively. For the scattering parameters used in this benchmarking, we relied on the default settings provided by ScattBO. To establish a reasonable starting point for the BO routine, we set the number of initialisation points to 34, guided by the $2 \cdot d + 2$ heuristic described in the Methods section (with $d = 16$ parameters). We tested this configuration against three target scattering patterns simulated from icosahedral AuNPs with diameters of 16 Å, 32 Å, and 48 Å.

The benchmarking results, detailed in the following subsections, indicated that combining both Q-space and r-space data, normalising to the highest intensity peak, and employing a mean-squared error (MSE) objective function, together with the *Sparse Axis-Aligned Subspaces Bayesian Optimization* (SAASBO[28]) algorithm, provided the most robust performance. This strategy formed the basis of our experimental approach at the

synchrotron, optimising the likelihood of rapidly converging to the desired AuNP structure under real experimental conditions.

BO algorithm

As discussed in the main manuscript, BO often struggles with increasing dimensionality. In our case, the parameter space spans $\mathbb{R}^{16}$, why we must use advanced high-dimensional BO methods. We considered two algorithms which have shown promise in recent research: Hvarfner's D-scaled *p(l)*(VanillaBO[29]) and SAASBO[28].[30]

As seen in Figure S1A VanillaBO achieved faster per-prediction times (~10 seconds) but it required more experimental evaluations to reach the target structure. Given that each real-world experimental run takes approximately 17 minutes, this increased number of experiments is costly in practice. By contrast, SAASBO—although slower per prediction (~100 seconds)—converged to the target with fewer total experiments. This improved convergence efficiency is crucial in a real experimental setting. We anticipate that this performance advantage of SAASBO would be even more pronounced when dealing with experimental, noisy scattering data in a more complex chemical space, compared to the simulated scattering data in a relatively simple chemical space. Indeed, SAASBO computes an approximate posterior over model hyperparameters (which translates to better uncertainty estimates), and has stood the test of time in other high-dimensional BO benchmarks.[30] Figure S1B–C confirms this by showing that the best structure from using SAASBO describes the target scattering data better compared to using VanillaBO.

Consequently, we selected the SAASBO algorithm for our experiments. Its potential to reduce the overall number of required experimental evaluations outweighed the longer computational time per iteration.

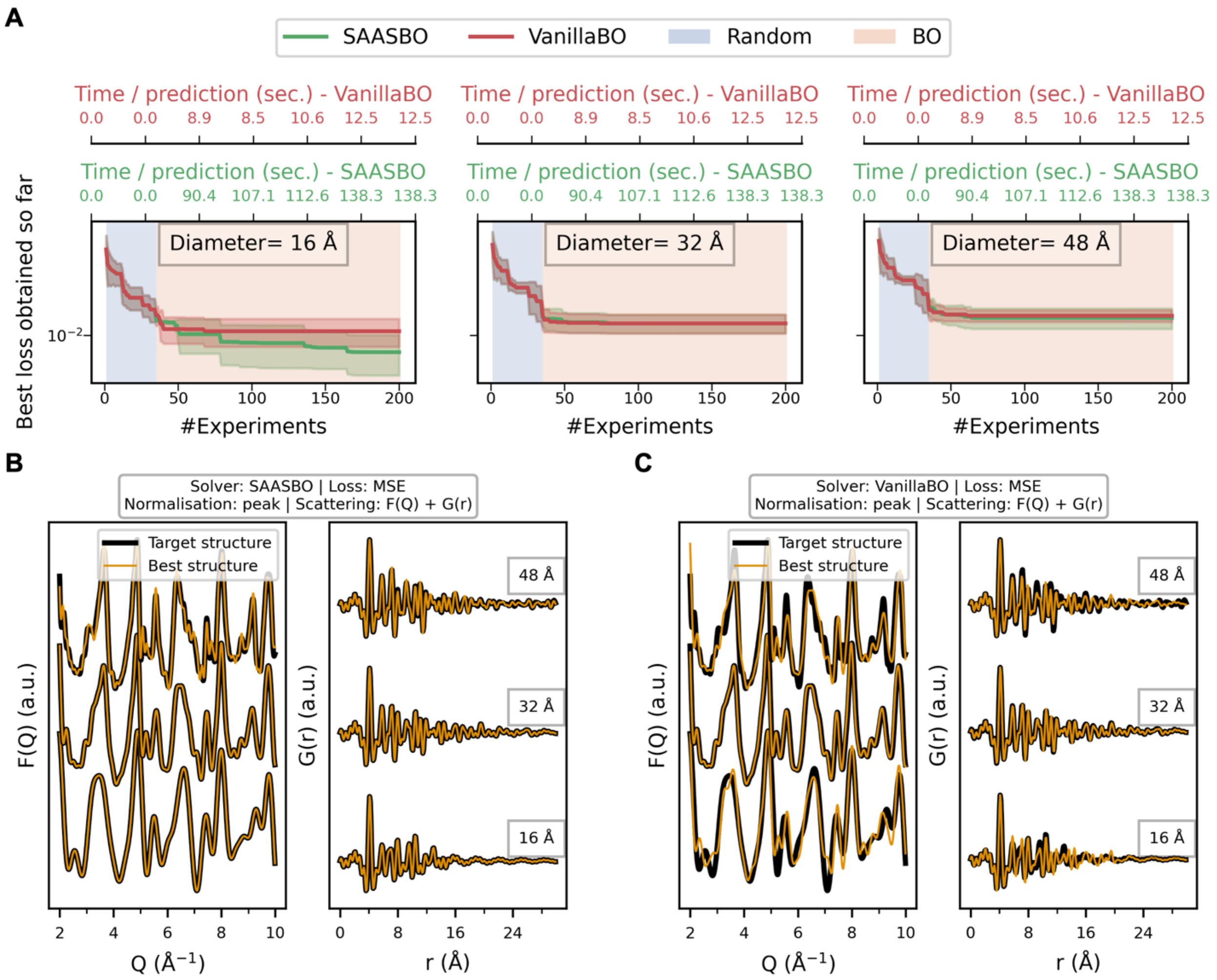

Figure S1 | Benchmarking the choice of BO algorithm. A) Convergence profiles showing the best loss value achieved as a function of the number of experiments for three distinct target scattering patterns and two different BO algorithms (SAASBO and VanillaBO). Each data point represents the mean of five independent runs, with shaded regions indicating one standard deviation. The top horizontal axis displays the computational time (in seconds) required by the BO algorithm to propose the next experimental parameters. The initial, blue-shaded region corresponds to a random exploration phase, while the subsequent, red-shaded region indicates the BO-driven optimisation stage. B–C) Comparison of the target scattering patterns in Q-space (left panels in each sub-figure) and r-space (right panels in each sub-figure) with those obtained from the best synthesis parameters identified by B) SAASBO and C) VanillaBO. Both approaches used identical normalisation (peak), objective

function (MSE), and scattering domains (combined F(Q) and G(r))—the hyperparameters that were selected for the actual synchrotron experiments.

Scattering function

In the context of characterising NPs, the choice of scattering function—whether to rely solely on reciprocal-space data (F(Q)) or real-space data (G(r))—is non-trivial. Conventional diffraction techniques primarily utilise Q-space data, which is well-suited for crystalline materials. The peaks observed in F(Q) provide a direct fingerprint of the material's phases, enabling the identification of by-products (here, referring to unwanted minority products), unit-cell changes, and variations in crystallite size through shifts in peak positions and peak broadening. This makes Q-space data valuable for systems with relatively well-defined long-range order.

However, NPs do not exhibit the pronounced periodicity associated with bulk crystalline lattices. In such cases, TS with PDF analysis in real space can offer critical insights. PDF data reveal local atomic arrangements, providing a clear picture of defects, disorder, NP sizes, and the intrinsic nanoscale structure that cannot be easily discerned from reciprocal-space data alone.

Our *in silico* benchmarking (Figure S2) did not conclusively indicate that any single domain (F(Q) or G(r)) consistently outperforms the other for guiding the optimisation process. Direct comparisons of the loss values between F(Q)- and G(r)-based analyses are not straightforward, as the magnitudes and scales of these metrics differ. Instead, the primary goal is to ascertain whether the chosen data domain informs the BO algorithm, enabling it to learn and improve its predictions over successive iterations.

In light of these observations, we opted to integrate both F(Q) and G(r) data into our experimental protocol. This hybrid approach leverages the strengths of Q-space data—facilitating the identification of by-products and providing a clear crystallographic fingerprint—while simultaneously drawing on the sensitivity of r-space data to local structural features, defects, and nanoscale order.

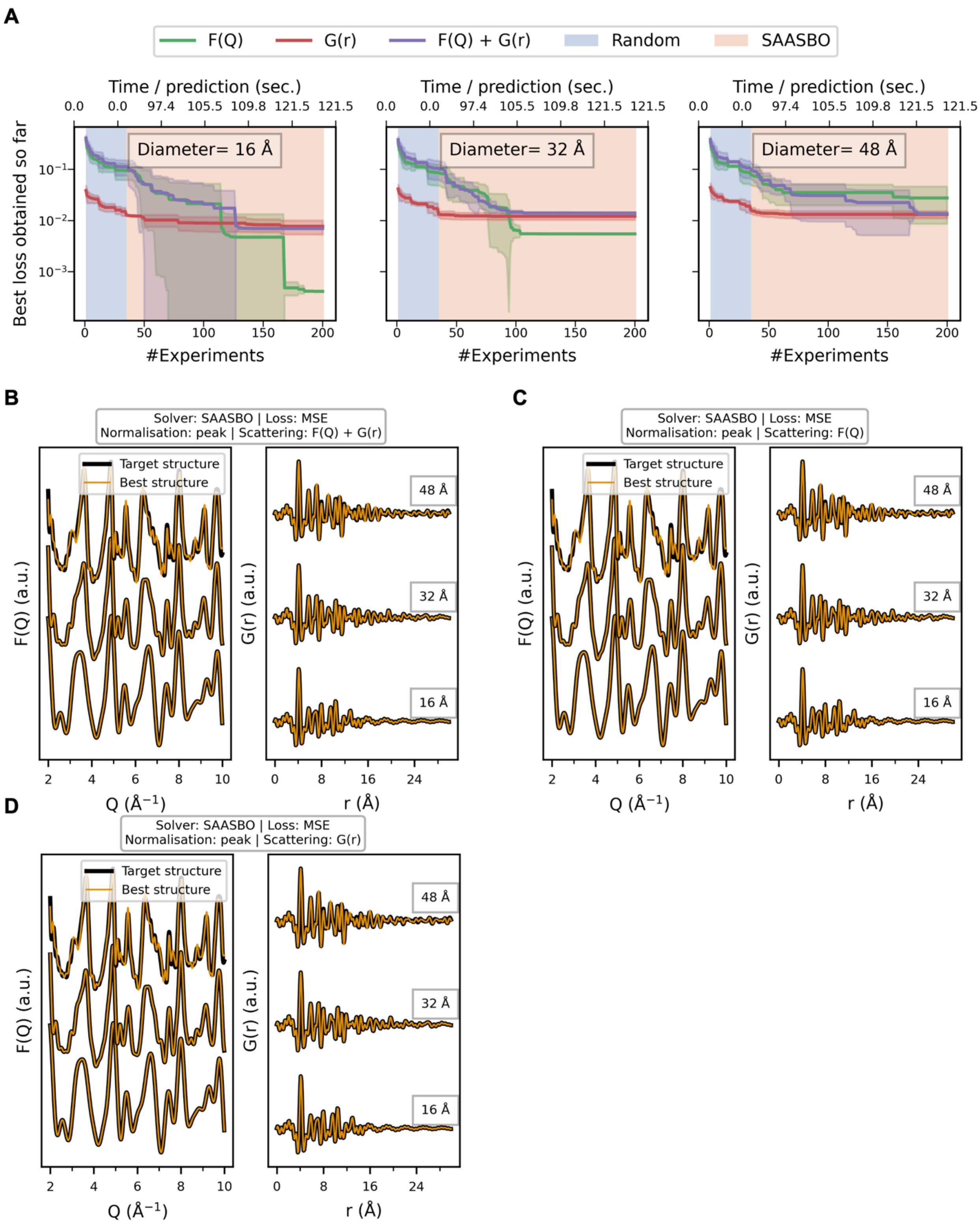

Figure S2 | Benchmarking the choice of scattering function. A) Convergence profiles showing the best loss value achieved as a function of the number of experiments for three distinct target scattering patterns and three

different scattering functions (F(Q), G(r), and a combination of them). Each data point represents the mean of five independent runs, with shaded regions indicating one standard deviation. The top horizontal axis displays the computational time (in seconds) required by the SAASBO algorithm to propose the next experimental parameters. The initial, blue-shaded region corresponds to a random exploration phase, while the subsequent, red-shaded region indicates the BO-driven optimisation stage. B–D) Comparison of the target scattering patterns in Q-space (left panels in each sub-figure) and r-space (right panels in each sub-figure) with those obtained from the best synthesis parameters using B) a combination of F(Q) + G(r), C) only G(r), and D) only F(Q). All approaches used identical normalisation (peak), objective function (MSE), and BO algorithm (SAASBO)—the hyperparameters that were selected for the actual synchrotron experiments.

Data normalisation

To assess the influence of data normalisation on BO performance, we considered three distinct approaches:

No normalisation (none):

Retaining absolute intensity values preserves the full informational content of the scattering patterns. This approach is ideal because it avoids loss of information. However, implementing it on experimental data is complex. Maintaining absolute counts throughout data processing is challenging, especially when subtracting incoherent scattering. Here, we use PDFgetX3[31] for incoherent scattering subtraction which perform *ad hoc* subtractions rather than explicitly modelling incoherent contributions.

Peak normalisation (peak):

Scaling the data so that its highest peak has intensity = 1 is straightforward and computationally efficient. Although it sacrifices some absolute intensity information, key structural features remain evident in both Q-space and r-space data, enabling the BO algorithm to identify meaningful patterns.

Rescaling to the [-1, 1] range (ML standard):

This method, often employed in ML workflows, normalises the data across a uniform scale. While straightforward, it diminishes the role of overall intensity and alters the baseline, which is related to the size of the NPs.

From our *in silico* benchmarking (Figure S3), it appears that both *no normalisation* and *peak normalisation* lead to rapid and reliable convergence of the BO algorithm within 200 experiments. In contrast, normalising to the [-1, 1] range results in slower learning and less accurate final solutions. Although omitting normalisation entirely might offer marginal benefits, the practical difficulties associated with preserving absolute intensities are considerable. By comparison, peak normalisation offers a manageable compromise, preserving essential structural information while ensuring a simpler and more robust data processing pipeline. Consequently, we adopted peak normalisation in our experiments.

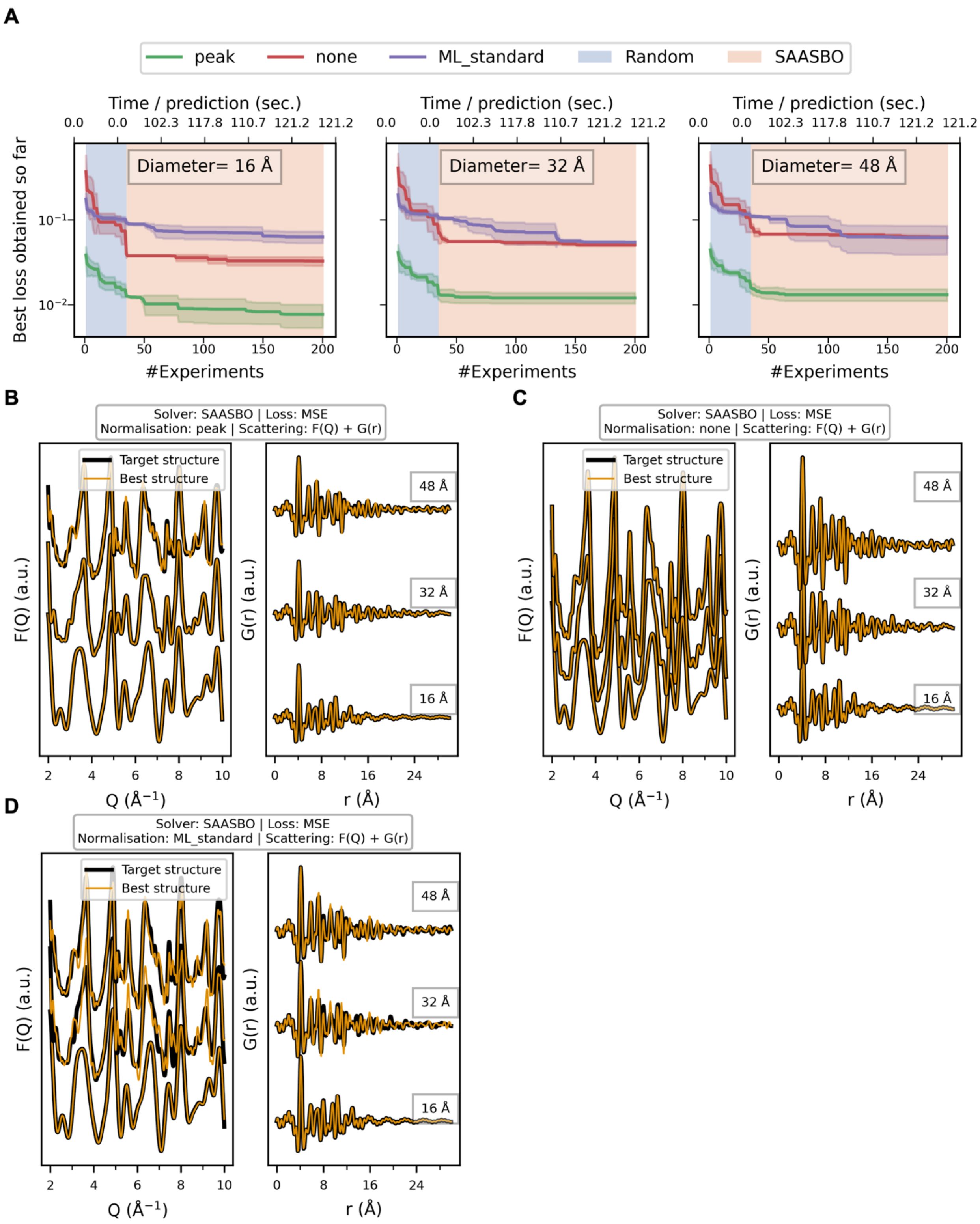

Figure S3 | Benchmarking the data normalisation strategy. A) Convergence profiles showing the best loss value achieved as a function of the number of experiments for three distinct target scattering patterns and three

different normalisation strategies (no normalisation, normalising to highest peak, and normalising the data to be between -1 and 1). Each data point represents the mean of five independent runs, with shaded regions indicating one standard deviation. The top horizontal axis displays the computational time (in seconds) required by the SAASBO algorithm to propose the next experimental parameters. The initial, blue-shaded region corresponds to a random exploration phase, while the subsequent, red-shaded region indicates the BO-driven optimisation stage. B–D) Comparison of the target scattering patterns in Q-space (left panels in each sub-figure) and r-space (right panels in each sub-figure) with those obtained from the best synthesis parameters using B) a normalisation to the highest peak, C) no normalisation, and D) normalisation between -1 and 1. All approaches used identical objective function (MSE), BO algorithm (SAASBO), and scattering domains (combined F(Q) and G(r)—the hyperparameters that were selected for the actual synchrotron experiments.

Objective function

We evaluated two different objective functions for guiding BO process: the MSE and the weighted profile agreement factor (R_{wp}). The MSE, described in the Methods section of the main manuscript, is a commonly employed metric in the ML community. By contrast, R_{wp}, defined as:

$$R_{wp} = \sqrt{\frac{\sum_{i=1}^{n}[I_i^{exp} - I_i^{target}]^2}{\sum_{i=1}^{n} I_i^{exp\,2}}} \cdot 100\ \% \qquad (1)$$

is frequently used in the scattering community. Here, I^{exp} and I^{target} represent the experimental and target intensities at the ith data point, respectively, and the sum extends over all n points in the scattering pattern.

Our *in silico* benchmarking (Figure S4) indicates that the choice of objective function—MSE or R_{wp}—does not significantly affect the rate at which the BO algorithm converges. Given its widespread use and straightforward interpretation within the ML domain, we opted to employ the MSE as our objective function for the actual experiments.

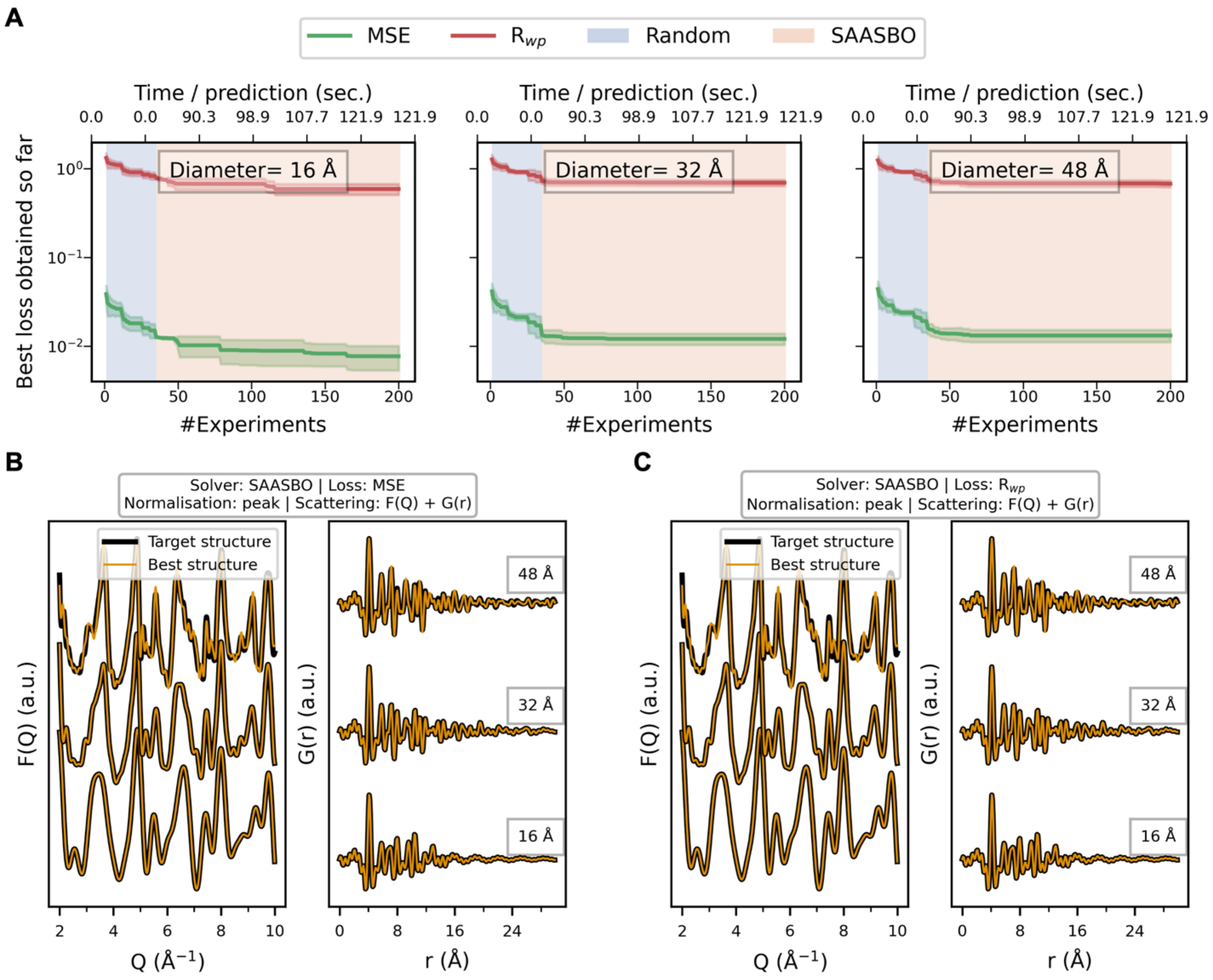

Figure S4 | Benchmarking the choice of objective function. A) Convergence profiles showing the best loss value achieved as a function of the number of experiments for three distinct target scattering patterns and two objective functions (MSE and R_{wp} value). Each data point represents the mean of five independent runs, with shaded regions indicating one standard deviation. The top horizontal axis displays the computational time (in seconds) required by the SAASBO algorithm to propose the next experimental parameters. The initial, blue-shaded region corresponds to a random exploration phase, while the subsequent, red-shaded region indicates the BO-driven optimisation stage. B–C) Comparison of the target scattering patterns in Q-space (left panels in each sub-figure) and r-space (right panels in each sub-figure) with those obtained from the best synthesis parameters using B) a MSE objective value and C) a R_{wp} objective value. All approaches used identical normalisation (peak),

BO algorithm (SAASBO), and scattering domains (combined F(Q) and G(r)—the hyperparameters that were selected for the actual synchrotron experiments.

Differences between benchmarking and synchrotron experiments

While the *in silico* benchmarking informed the selection of hyperparameters for ScatterLab, variations were introduced during the actual experiments conducted at the DanMAX beamline at MAX IV.

Scattering range:

In the benchmarking study, the scattering patterns were simulated using default values of ScattBO (Q_{range} of 2–10 $Å^{-1}$). In reality, the experiments at DanMAX allowed us to extend the Q_{range} to 0.5–15 $Å^{-1}$.

Target structures:

The benchmarking process targeted an icosahedral structure for AuNPs. In contrast, the synchrotron experiments focused on synthesising decahedral and FCC structures.

Experimental parameters:

In the benchmark, 16 parameters were considered, including temperature and various pump speeds. In practice, we omitted the heating element entirely and the addition of the first five chemicals under the assumption that the reaction will not be initialised before the addition of the last chemical (Au precursor). By reducing the parameter space from 16 to 11, we could in line with the $2 \cdot d + 2$ heuristic, conduct fewer initial random experiments before initialising BO.

Transition from predefined chemistry rules to experimental reality:

Although the above-mentioned variations between the *in silico* benchmark and actual experiments highlight minor discrepancies, these differences are relatively insignificant compared to the fundamental distinction between predefined chemistry rules and real-world chemical synthesis. Ideally, benchmarks should be conducted under actual experimental conditions. However, in the absence of this capability, our current benchmark provides the most practical alternative for guiding experimental planning.

F: Repeating experiment #41

Ensuring robustness in NP synthesis protocols is crucial for enabling other research groups to replicate and extend the work. Recent discussions on reproducibility in automated laboratories highlight how programming languages, process abstractions, and hardware variability affect both *repeatability* (consistency within a single setup) and *reproducibility* (consistency across different setups).[32] If a robotic platform proves reliable enough for a standardised script to be shared and executed elsewhere with equivalent outcomes, it could substantially mitigate current reproducibility challenges in materials chemistry.[33-35]

Here, we focus on validating the *repeatability* of our SDL repeating two distinct experiments. Firstly, we repeat the synthesis parameters from experiment #41 after concluding the SDL campaign that targeted the ∼5 nm decahedral AuNP. As shown in Figure S5, the original and repeated datasets—along with the target scattering pattern—are broadly consistent, indicating stable performance under identical conditions. We also did a measurement with extended measurement time (15 minutes) as seen in Figure S6. This longer collection period increased counting statistics and thereby produced higher-quality scattering data.

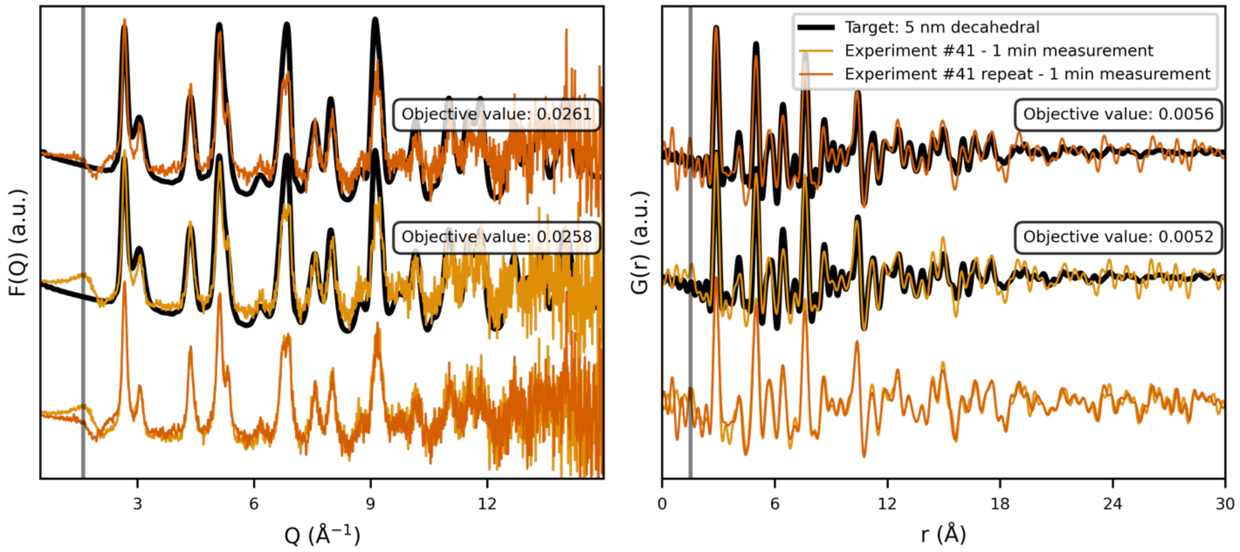

Figure S5 | Repeatability test. Scattering patterns F(Q) (left) and G(r) (right) for experiment #41 during two identical experiments: first experiment (yellow) and a repeated experiment with identical parameters (orange). From top to bottom are reported the comparison of the first experiment and the target, the repeated experiment and the target, and finally the two experiments overlapped.

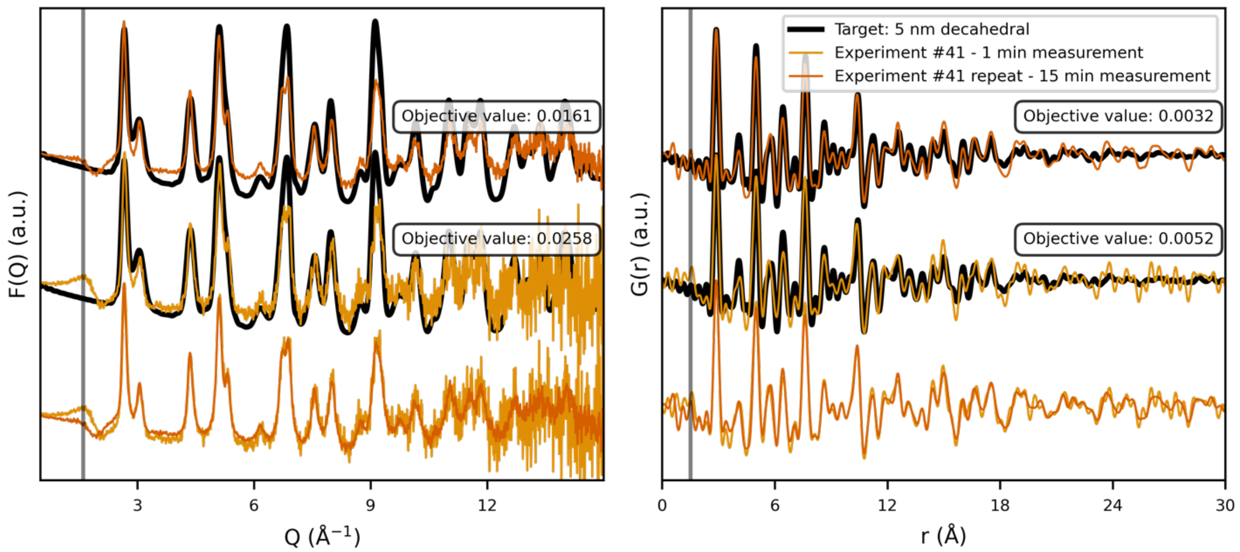

Figure S6 | Repeatability test with extended measurement time. Scattering patterns F(Q) (left) and G(r) (right) for experiment #41 under two conditions: a 1-minute measurement (yellow) and a repeated synthesis measured for 15 minutes under identical parameters (orange). The 15-minute dataset exhibits improved counting statistics. From top to bottom are reported the comparison of the first experiment and the target, the repeated experiment and the target, and finally the two experiments overlapped.

A second example of repeatability is given in Figure S7. Again, the synthesis is performed twice with identical parameters and yields comparable scattering patterns, demonstrating that the reaction is repeatable.

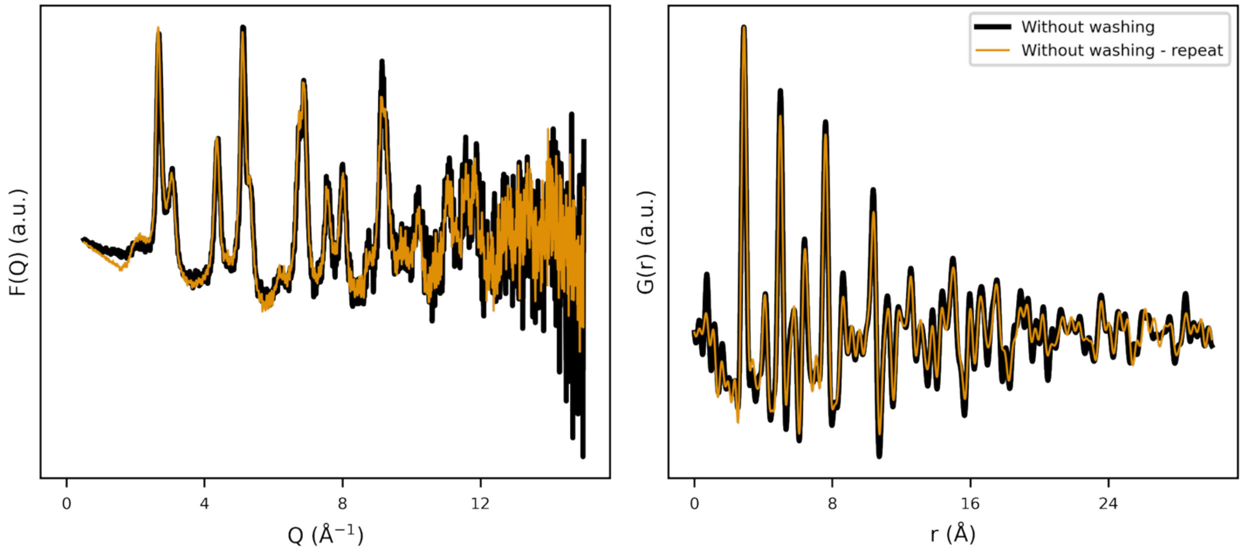

Figure S7 | Repeatability test. Comparison of scattering patterns F(Q) (left) and G(r) (right) for two experiments under identical synthesis conditions.

Looking ahead, we will assess the *reproducibility* of our SDL by systematically altering key components or transferring the protocol to different hardware setups and observing whether consistent results can still be

obtained. Such future studies will clarify how to maintain reliability when operational or procedural details are modified.

G: Modelling of the scattering data from experiment #41

We employed the finite clusters modelling strategy (see Methods, "*Finite Clusters*") to interpret the scattering data for experiment #41, using DebyeCalculator[36] to fit an extensive library of AuNP clusters. Figure S8 (top) plots the R_{wp} values against the number of atoms for four structural motifs—octahedral, icosahedral, decahedral, and FCC. Decahedral clusters (in red diamond) consistently yield the lowest R_{wp} values, indicating they best match the experimental data. Notably, multiple decahedral clusters of approximately 3000 atoms produce similarly good fits to the experimental data, preventing the identification of a single, unique model. If greater structural certainty is desired, complementary techniques (e.g. small-angle X-ray scattering or transmission electron microscopy) could provide further morphological details and help distinguish between closely related decahedral arrangements.

Further comparison reveals that the "best" fitting decahedral structure (3766 atoms) and the "target" decahedral structure (2706 atoms) produce nearly indistinguishable fits (Figure S8B–C). This parallels our observation that closely related decahedral motifs can have comparable scattering profiles. Although the "target" scattering pattern initially guided ScatterLab, we cannot guarantee that it represents a structure which can be synthesised. Nevertheless, the results confirm that a structure with a nearly identical scattering pattern was indeed synthesised.

We repeated the analysis on the scattering patterns obtained with identical conditions as experiment #41 but with 15 minutes acquisition time, providing higher quality data. Figure S9 shows the analogous R_{wp} plot for these longer measurements, again confirming that decahedral motifs dominate. The improved signal-to-noise ratio yields modestly lower R_{wp} values, yet we draw the same qualitative conclusion: multiple decahedral models fit

equally well, and distinguishing between them would likely require complimentary data (e.g. from transmission electron microscopy).

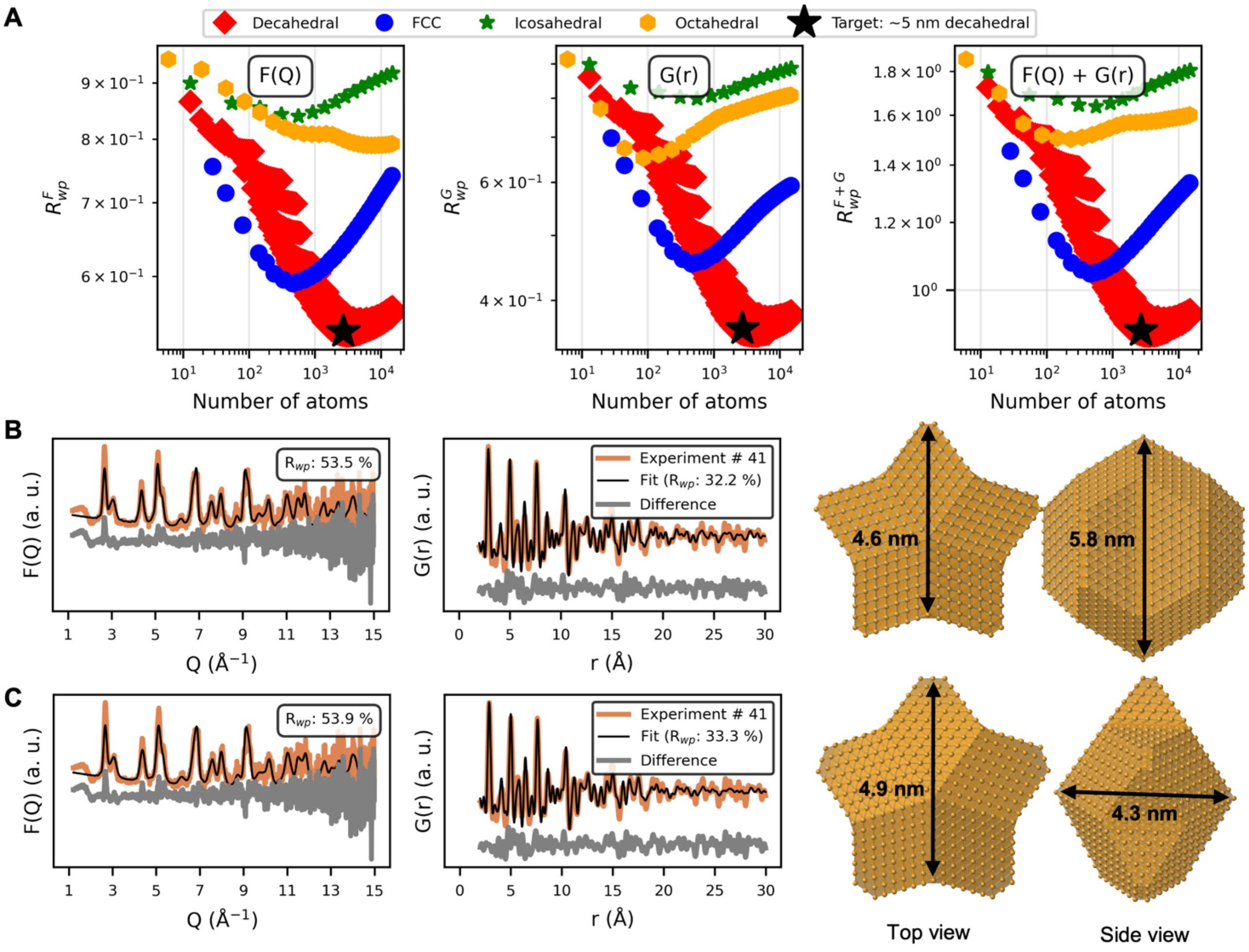

Figure S8 | A cluster-mining approach for modelling the scattering data of experiment #41 during the SDL campaign. A) Comparison of the best-fit results for four structural motifs—octahedral, icosahedral, decahedral, and FCC—across various cluster sizes. The R_{wp} metric is plotted against the total number of atoms in each model. Notably, decahedral structures (red diamonds) provide the lowest R_{wp} values, suggesting that they best describe the experimentally measured scattering data. B) Experimental F(Q) and G(r) data from experiment #41 (orange) overlaid with the fit of the best-fitting decahedral structure (black). The bottom panels display the difference curves (grey). C) Experimental data (orange) compared against the “target” decahedral model (black). The 3D

renderings (right) illustrate top and side views of the decahedral cluster, with approximate dimensions indicated. Both the scaling of the F(Q) and G(r) data and the atomic displacement parameter (ADP) value were fitted.

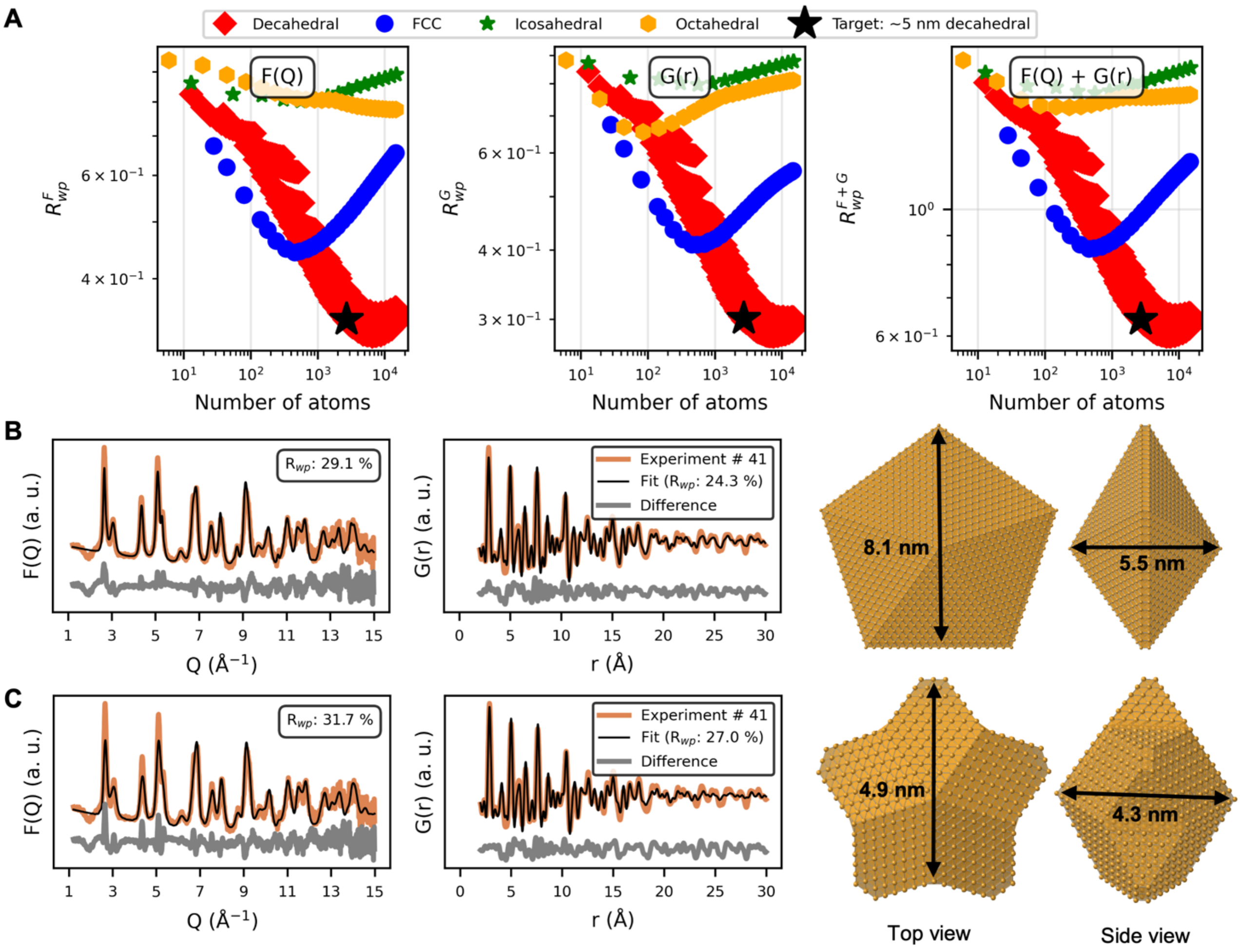

Figure S9 | A cluster-mining approach for modelling the scattering data of the repeated experiment #41 with 15 min measurement time. A) Comparison of the best-fit results for four structural motifs—octahedral, icosahedral, decahedral, and FCC—across various cluster sizes. The R_{wp} metric is plotted against the total number of atoms in each model. Notably, decahedral structures (red diamonds) provide the lowest R_{wp} values, suggesting that they best describe the experimentally measured scattering data. B) Experimental F(Q) and G(r) data from experiment #41 (orange) overlaid with the fit of the best-fitting decahedral structure (black). The

bottom panels display the difference curves (grey). C) Experimental data (orange) compared against the "target" decahedral model (black). The 3D renderings (right) illustrate top and side views of the decahedral cluster, with approximate dimensions indicated. Both the scaling of the F(Q) and G(r) data and the atomic displacement parameter (ADP) value were fitted.

H: Behind the scenes: how ScatterLab navigated AuNP synthesis variables

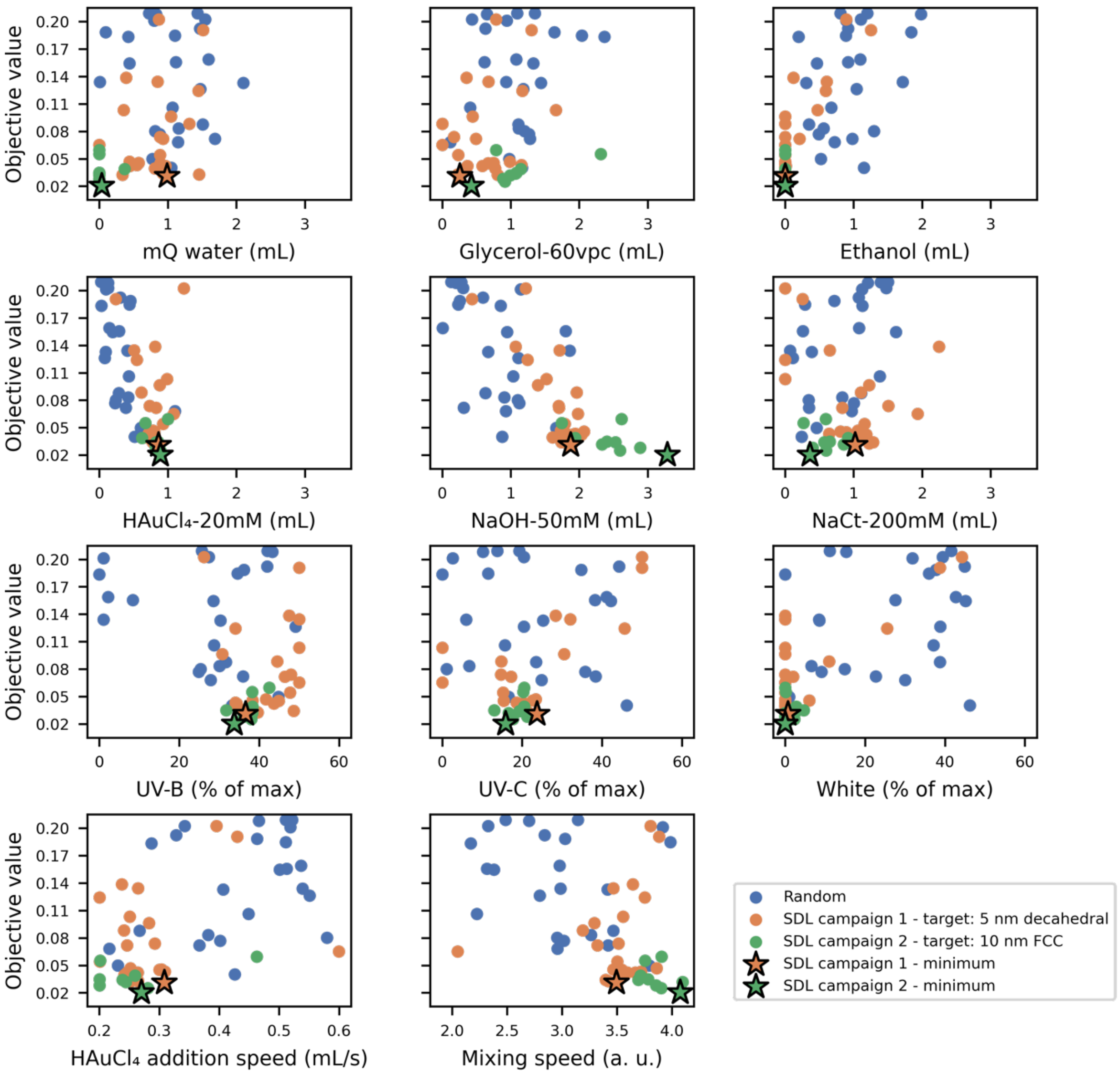

Figure S10 | Synthesis parameters versus objective values. Top panels) Water (left), glycerol (middle), and ethanol (right) content in mL. Middle top panels) $HAuCl_4$ (left), NaOH (middle), and NaCt (right) content in mL. Middle lower panels) UV–B (left), UV–C (middle), and white (right) light-emitting diode (LED) power (100 % corresponds to full intensity). Lower panels) $HAuCl_4$ precursor addition speed (mL/s) (left) and mixing speed (right) in arbitrary units. All plotted against the objective values for each experiment. Objective values for the random and decahedral experiments (blue, orange) are evaluated against the decahedral target; FCC experiments (green) are evaluated against the FCC target. Values can therefore be compared across campaigns only with care. An interactive plot of the Figure is shared as part of the associated code.

Notably, the white LED intensity drops to 0% during the BO phase, and after experiment #26, the mixer failed, rendering mixing speed an unreliable parameter. We could have fixed the mixer by swapping in a spare unit, but doing so would have consumed valuable beamtime (challenges 1–2 in section A). Consequently, we chose to continue without a functioning mixer, prioritising further experiments over performing an on-site hardware fix.

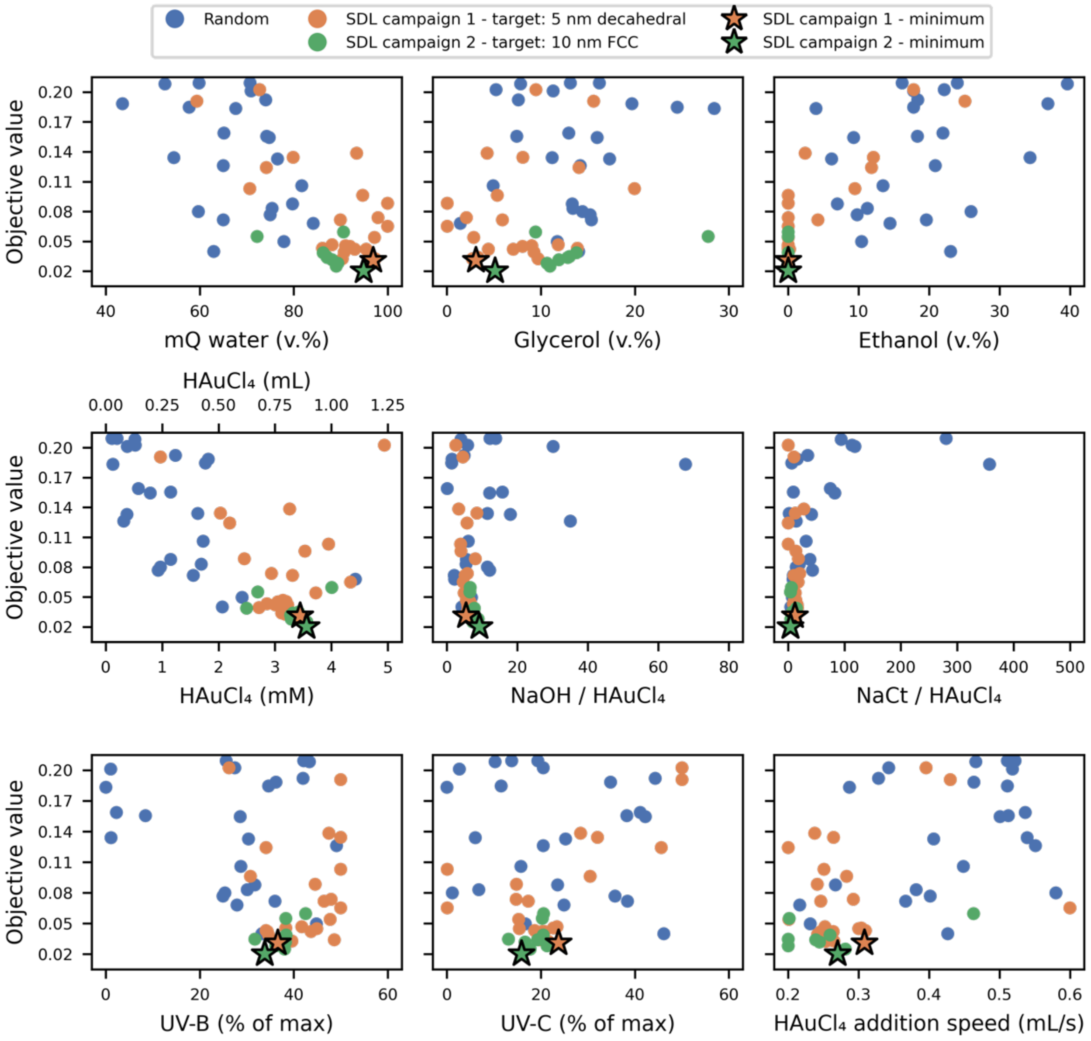

Figure S11 | Synthesis parameters versus objective values. Top panels) Water (left), glycerol (middle), and ethanol (right) content in v.%. Middle panels) $HAuCl_4$ content in mM and mL (left), NaOH / $HAuCl_4$ ratio (middle), and NaCt / $HAuCl_4$ ratio (right). Lower panels) UV–B (left), and UV–C (middle) lamp power (100 % corresponds to full intensity), and $HAuCl_4$ precursor addition speed (mL/s) (right). All plotted against the objective values for each experiment. Objective values for the random and decahedral experiments (blue, orange) are evaluated against the decahedral target; FCC experiments (green) are evaluated against the FCC target. Values can therefore be compared across campaigns only with care. An interactive plot of the Figure is shared as part of the associated code.

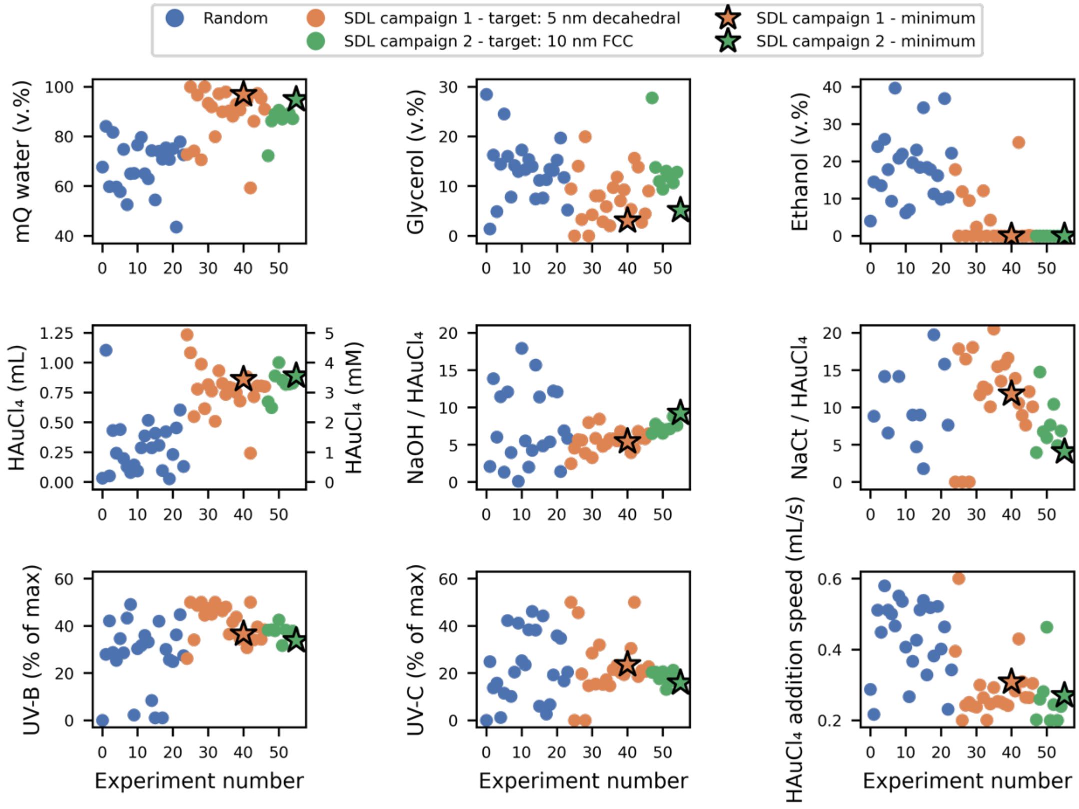

Figure S12 | Synthesis parameters versus experiment number. Top panels) Water (left), glycerol (middle), and ethanol (right) content in v.%. Middle panels) $HAuCl_4$ content in mM and mL (left), NaOH / $HAuCl_4$ (middle), and NaCt / $HAuCl_4$ (right) ratios restricted to be between 0 and 20. Lower panels) UV–B (left), and UV–C (middle) lamp power (100 % corresponds to full intensity), and $HAuCl_4$ precursor addition speed (mL/s) (right). All plotted against the experiment number. An interactive plot of the Figure is shared as part of the associated code.

I: Manual, human-operated synthesis

	H_2O (mL)	Glycerol (mL)	Ethanol (mL)	$HAuCl_4$ (mL)	NaOH (mL)	NaCt (mL)	Note	Result
**1**	0.396	0.102	0	0.344	0.750	0.408	Exp #41	Collapsed
**2**	0.014	1.314	0	0.146	0.170	0.356	Exp #56	Collapsed

**3**	0.498	0	0	0.344	0.750	0.408	Exp #41 excl. glycerol	Collapsed
**4**	0.184	0	0	0.146	0.170	0.356	Exp #56 excl. glycerol	Collapsed
**5**	0.804	0.102	0	0.344	0.750	0	Exp #41 excl. NaCt	Stable*
**6**	0.160	1.314	0	0.146	0.170	0	Exp #56 excl. NaCt	Collapsed
**7**	1.146	0.102	0	0.344	0	0.408	Exp #41 excl. NaOH	Collapsed
**8**	1.328	1.314	0	0.146	0	0.356	Exp #56 excl. NaOH	Stable**

Table S4 | Synthesis parameters and stability outcomes of eight manual experiments. Listed are the volumes of each reagent (in millilitres, total volume 2 mL) and the resulting stability after 24 hours. Rows 1 and 2 reproduce the synthesis protocols from experiments #41 and #56, whereas rows 3–4 omit glycerol, rows 5–6 omit NaCt, and rows 7–8 omit NaOH. Only syntheses 5 and 8 yield AuNP dispersions stable after 24 hours. The final colour (e.g. "red" or "purple") indicates the observed NP appearance. More information about the *chemicals* and *synthesis* measurements are provided in the following subsections. * Stable upon dilution (e.g. from 3.44 mM to 0.5 mM), remaining a stable colloid for over one month. **Not stable as a concentrated colloid but retains a consistent UV–Vis spectrum for at least one month after gentle homogenisation.

	**H_2O (v.%)**	**Glycerol (v.%)**	**Ethanol (v.%)**	**$HAuCl_4$ (mM)**	**NaOH / $HAuCl_4$**	**NaCt / $HAuCl_4$**	**Note**	**Result**
**1**	96.94	3.06	0	3.44	5.5	11.9	Exp #41	Collapsed
**2**	94.9	5.1	0	3.56	9.2	4.1	Exp #56	Collapsed
**3**	100	0	0	3.44	5.5	11.9	Exp #41 excl. glycerol	Collapsed
**4**	100	0	0	3.56	9.2	4.1	Exp #56 excl. glycerol	Collapsed
**5**	96.94	3.06	0	3.44	5.5	0	Exp #41 excl. NaCt	Stable*
**6**	94.9	5.1	0	3.56	9.2	0	Exp #56 excl. NaCt	Collapsed
**7**	96.94	3.06	0	3.44	0	11.9	Exp #41 excl. NaOH	Collapsed
**8**	94.9	5.1	0	3.56	0	4.1	Exp #56 excl. NaOH	Stable**

Table S5 | Synthesis parameters and stability outcomes of eight manual AuNP experiments. Synthesis parameters reported in volume percent (v.%), $HAuCl_4$ molarity, or selected ratios relative to $HAuCl_4$. Rows 1 and 2 mimic experiments #41 and #56, while rows 3–4 exclude glycerol, rows 5–6 exclude NaCt, and rows 7–8

exclude NaOH. Only syntheses 5 and 8 produce stable AuNP suspensions after 24 hours. The final colour (e.g. “red” or “purple”) indicates the observed NP appearance. More information about the *chemicals* and *synthesis* measurements are provided in the following subsections. * Stable upon dilution (e.g. from 3.44 mM to 0.5 mM), remaining a stable colloid for over one month. **Not stable as a concentrated colloid but retains a consistent UV–Vis spectrum for at least one month after gentle homogenisation.

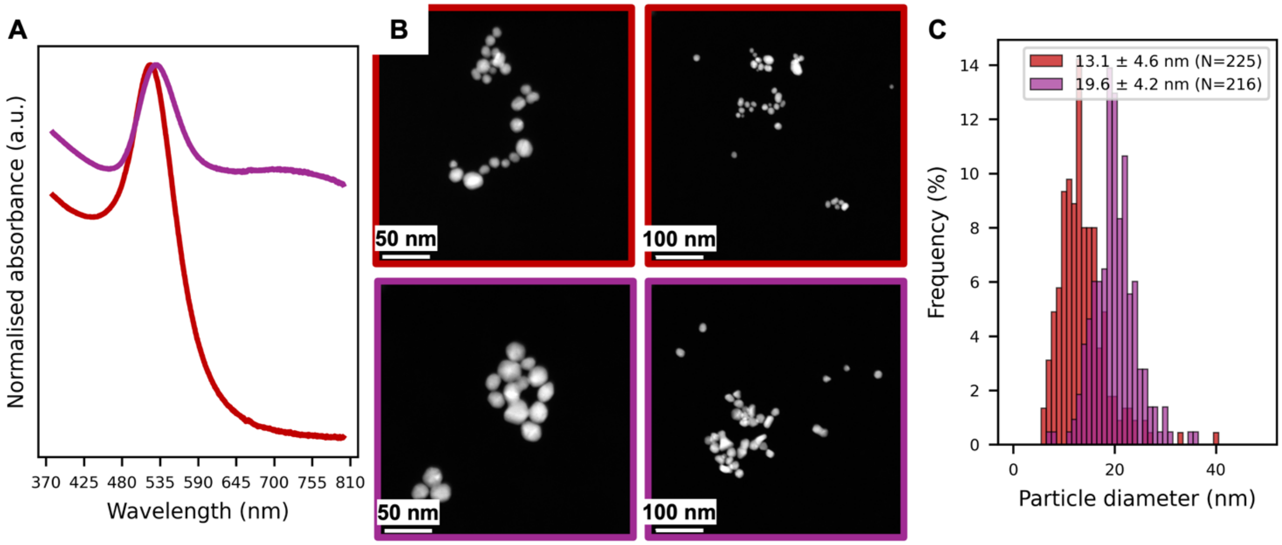

Figure S13 | UV–Vis and Scanning Transmission Electron Microscope (STEM) characterisation of stable AuNPs (rows 5 and 8 in Table S4+5) 24 hours post-synthesis. A) UV–Vis spectra indicate minimal agglomeration and reveal that the AuNPs from row 5 (red) are smaller than those from row 8 (purple)), and the later show relatively more agglomeration in line with the UV-vis spectra with more pronounced features in UV-vis at higher wavelengths. Samples were diluted to 0.5 mM equivalent of $HAuCl_4$ for measurements. B–C) STEM micrographs confirm that both dispersions consist of spherical AuNPs, further supporting the conclusion that the particles obtained from the experimental conditions in row 5 in Table S4 are smaller than the particles obtained from the experimental conditions in row 8 in Table S4 and there is minimal agglomeration. More information about the *UV–Vis* and *STEM* measurements are provided in the following subsections.

Long-term stability

AuNPs synthesised under the conditions in row 8 appeared to sediment. This can be attributed to the largest materials obtained. After gentle homogenisation, their UV–Vis spectrum remained effectively unchanged after one month of storage at room temperature. By contrast, AuNPs from row 5 (3.44 mM $HAuCl_4$) showed signs of instability after one month at room temperature: following homogenisation and/or dilution, their overall absorbance decreased, indicating possible agglomeration. Nevertheless, when diluted to ~0.5 mM the day after synthesis, the suspension remained stable for at least a month. Notably, diluting highly concentrated samples is a common practice to maintain colloidal stability, and AuNPs are typically stored in a refrigerator. Here, we deliberately stored them at room temperature to assess a "worst-case" scenario. Overall, the results show the promising stabilityu of the AuNPs prepared at high concentrations of $HAuCl_4$.

Chemicals

All chemicals were used as received: high purity water (mQ, Milli-Q, resistivity ≥ 18.2 MΩ·cm); $HAuCl_4 \cdot 3H_2O$ (99%, BLD Pharmatech); NaOH (Sigma Aldrich, reagent grade, ≥98%, pellets); trisodium citrate·$2H_2O$ (NaCt, ≥ 99.9% Sigma Aldrich, BioUltra); glycerol (bi-distilled 99.5%, VWR); HCl (puriss. ACS reagent, reag. ISO, reag. Ph. Eur. fuming, ≥37%, Sigma Aldrich); HNO_3 (puriss ≥ 65%, Sigma Aldrich).

Synthesis

The container used as reactors were disposable polystyrene UV–Vis cuvettes (1 cm wide, rectangular shape), de-dusted by flowing a jet of compressed air prior to the reaction. The magnets used for stirring (PTFE cylindrical stirrer bar, 8 x 3 mm) were cleaned with *aqua regia* (4:1; v:v; HCl:HNO_3) and washed with large amount of

Milli-Q water. The diluted *aqua regia* is corrosive contains traces of metal and must be discarded with care taking into account the regulation enforced in the working place (!).

Stock solutions were prepared in mQ water with 60 v.% glycerol, 20 mM $HAuCl_4$, 200 mM NaCt, 50 mM NaOH. The $HAuCl_4$ was added last under stirring. Experiments were performed under the controlled light of a photo-box (Puluz LED portable Photo Studio, PU5060EU, 60 cm x 60 cm x 60 cm, 60 W).[16, 37] The synthesis was left to perform for two hours in the photobox at ambient temperature. The samples for UV–Vis and STEM were prepared the day after. The samples were then sealed with Parafilm® and kept in a drawer at room temperature.

UV–Vis

The UV–Vis measurements were performed using a Thermo Scientific Genesys 10 s UV–Vis spectrophotometer in the range 290–800 nm on the as-prepared colloidal dispersions diluted from the as-prepared NPs to reach a final concentration in gold expected to be around 0.5 mM. Disposable polystyrene cuvettes were used (1 cm wide, rectangular shape). As blank, a mixture of the same chemicals as those mixed for the reaction were used but excluding base and citrate because those chemicals do not absorb in the range considered but using the same amount of alcohol as in the diluted sample (without $HAuCl_4$ added).

The plasmonic properties captured in the UV–Vis spectra of AuNPs depend on size, shape, concentration, but also on the media surrounding the NPs (e.g. interaction with unreacted precursors and/or solvent).[38, 39] While a detailed interpretation can be complex, several metrics have proved to be convenient to estimate the (relative) size and/or shape of AuNPs. For spherical AuNPs, the wavelength at the surface plasmon resonance (spr), λ_{spr}, i.e. the wavelength that correspond to the maximum of absorption in the range 500–700 nm (A_{spr}), decreases as the NP size decreases.[40] Higher λ_{spr} values can also indicate non-spherical NPs or agglomerated NPs. Equally, the ratio of the absorbance at 450 nm (A_{450}) and A_{spr}, i.e. A_{spr}/A_{450} decreases when the NP size decreases (for spherical and relatively small size NP with a well-defined A_{spr}). The ratio of the absorbance recorded at 650 nm,

A_{650}, and at the A_{spr}, A_{650}/A_{spr}, or the ratio of the absorbance recorded at 380 nm, A_{380}, and 800 nm, A_{800}, i.e. A_{380}/A_{800} give information on the stability of the colloids. The colloids tend to be more stable as the A_{650}/A_{spr} ratio decreases[41] or as the A_{380}/A_{800} ratio increases.[42] Finally, the intensity at 400 nm gives an indication on the relative yield.[43]

<u>STEM</u>

A FEI Talos F200X operated at 200 kV and equipped with High-Angle Annular Dark-Field detector was used for STEM imaging. The colloidal dispersion prepared by the above-described syntheses were directly dropped on copper TEM girds and after solvent evaporation, the measurements were performed.

<u>Transferable parameters between robotic and manual synthesis</u>

Parameter category	Transferable manually	Notes
**Reagent volumes (6 dimensions)**	Yes	Directly transferable
**Chemical addition speed (1 dimension)**	No	Robotic flow rates cannot be matched reliably by hand
**Mixing speed (1 dimension)**	No	Depends on mixing environment
**Light illumination (3 dimensions)**	No	Depends on reactor material, lamp distance, and lamp.

Table S6 | Transferability of synthesis parameters between robotic and manual synthesis.

J: Performance metrics for the SDL

In line with the perspective "*Performance metrics to unleash the power of self-driving labs in chemistry and materials science*" by Volk & Abolhasani,[44] we report performance metrics for ScatterLab. In order to democratise SDL research—particularly at facilities like synchrotrons, where beamtime is often limited to a few days or a week—it is vital to keep installation both inexpensive and fast. Therefore, we include an additional category detailing installation requirement. By highlighting these parameters, we hope to clarify who can undertake similar projects and to emphasise the importance of ease of setup for broader adoption.

**Degree of autonomy**	**Field of research**	Nanoparticles
	**Material studied**	Au
	**Max dimensionality**	11
	**Algorithm**	SAASBO[28]
	**Experimental platform**	Modular liquid robot
	**Degree of autonomy**	Closed-loop
**Lifetime[I]**	**Demonstrated unassisted lifetime (samples)**	7
	**Demonstrated assisted lifetime**	56
	**Theoretical unassisted lifetime**	~ 500 (Limited by a 10 L waste container)
	**Theoretical assisted lifetime**	Indefinite
**Throughput[I]**	**Demonstrated throughput (time/sample)**	44 min 7 s (incl. downtime)
	**Theoretical throughput**	17 min 1 s (excl. downtime)[II]
**Precision**	**Precision assessment method**	Continuous sampling
**Quantity**	**Maximum active quantity**	V_{tot} = 5 mL, V_{max}^{Au}=1.25 mL
	**Total materials per experiment**	5 mL (+16 mL H_2O for cleaning)
	**Total hazardous per experiment**	Up to 5 mL
	**Total high value per experiment**	N/A
**Algorithm performance**	**Trials to reach maximum**	41[III]
	**Model validation**	Cross validation
	**Feature analysis**	Simulated benchmark with 540 feature combinations of various data types, data normalisations, objective functions and BO algorithms.
	**Benchmarking**	
**Installation**	**Installation time (hardware+software+chemistry)** (months:days:hours)	Within 1 day
	**Installation time (person-hours)**	Robotics: ~4 hours x 2 people[IV]
		Chemistry: ~3 hours x 2 people[IV]
		Scattering: ~4 hours x 2 people[IV]
	**Team size** Number of individuals involved	2 robot experts, 2 synthesis chemists, 1 beamline scientist, 1 all-round expert (chemistry, scattering, machine learning)
	**Cost of replication** Estimated setup cost	~€2500
	**Space requirement** Required area for setup	~W70 x L45 x H60 cm
	**Pre-requisites** Infrastructure needs	Power and network

Table S7 | Performance metrics for ScatterLab.

[I]Our performance metrics reflect a *one-shot* installation and deployment, where ScatterLab is integrated at a synchrotron for a limited period and run without extensive iterative optimisation beyond the initial setup.

[II]The "theoretical throughput" is based on average problem-free SDL cycles (see "*Time profiling of the individual operations in ScatterLab*"). It may be further optimised through hardware- or software parallelisation.

[III]We demonstrate that this number can be lowered using active learning.

[IV]These are approximate times required to set up each part of ScatterLab. The robotic modules and chemical preparations proceeded in parallel, while the synchrotron beamline calibration could only begin after the rest of ScatterLab was in place.

K: Time profiling of the individual operations in ScatterLab

As shown in Figure S14, ScatterLab completes each closed-loop cycle in approximately 17 minutes (1021 s). Within this timeframe, ~9 minutes are devoted to robotic synthesis (including 5-minute UV/white LED illumination), followed by 1 min each for 'blank' and sample measurements, and ~3.5-minute of washing to prevent cross-contamination. The BO algorithm then requires ~2 minutes to propose the next set of synthesis parameters.

Several strategies can mitigate these time overheads. For example, the BO routine could be distributed across multiple graphical processing units rather than relying on a single device. Likewise, hardware parallelisation—operating multiple independent synthesis modules in tandem—can reduce idle time by enabling simultaneous reactions. Although the washing step is inherently less amenable to direct speed-ups, it can overlap with other tasks like data processing or the BO routine. Moreover, a workflow manager (e.g. PerQueue,[45] FireWorks,[46] or Jobflow[47]) could launch subsequent operations (washing, BO proposals, etc.) the moment any resource becomes free, rather than waiting for the entire current cycle to finish. We estimate that software parallelisation alone could save 4 minutes per cycle—bringing the total duration to ~13 minutes—while doubling the number of

robotic modules for hardware-parallel syntheses might reduce a further 4.5 minutes, yielding a combined 50% speed-up overall.

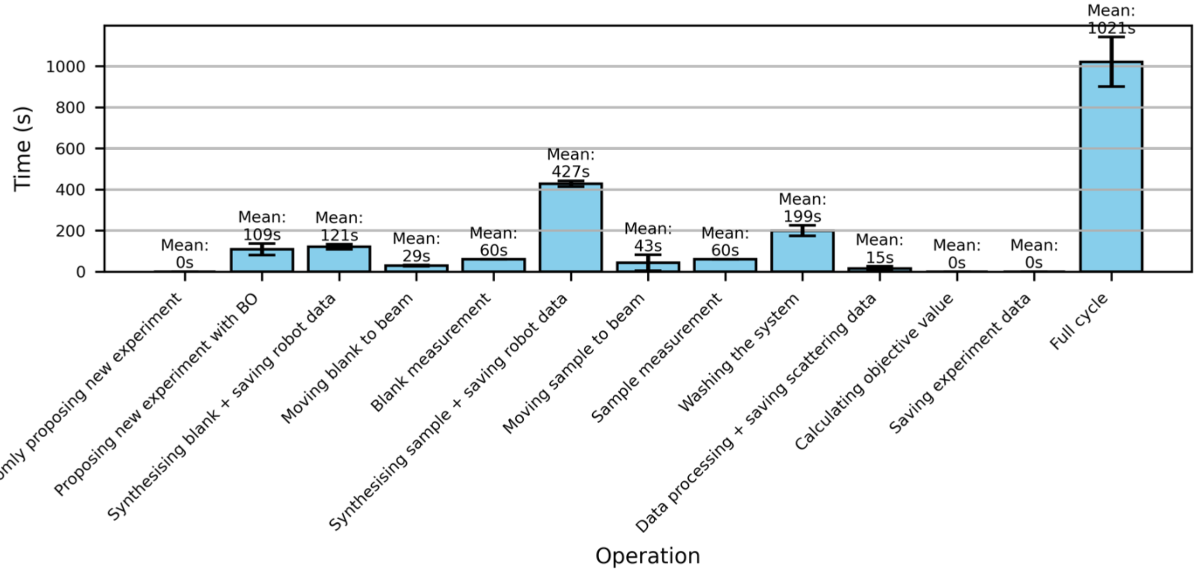

Figure S14 | Timing of individual ScatterLab operations. We categories the ScatterLab campaign into 14 discrete operations, each subjected to time profiling. The figure presents the average duration and standard deviation of these steps. There is also a one-off overhead of 9.08 ± 1.14 s for initialising the robot and synchrotron control software. Additionally, a 10 s window is allocated after each experiment, allowing a human operator to mark the run as ‘failed’ or ‘completed’; in the absence of input, the experiment defaults to ‘completed’.

L: Influence of illumination duration with white and UV light

Figure S15 compares scattering patterns obtained from two synthesis experiments performed under identical conditions, except for the duration of illumination with white and UV light (3 min, yellow curve; 5 min, black curve). The results indicate that illumination does not impact the final AuNP structure beyond 3 min.

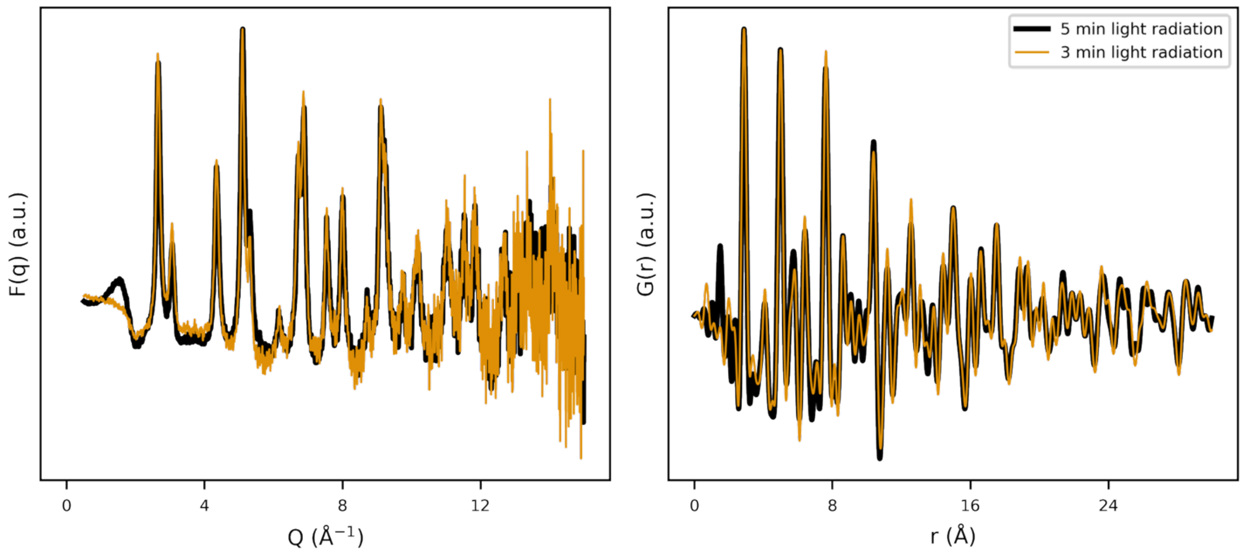

Figure S15 | Effect of illumination duration on AuNP structure. Comparison of scattering patterns F(Q) (left) and G(r) (right) obtained from experiment differing only in white and UV light illumination duration. yellow curve: 3 min (yellow curve) and 5 min (black curve).

M: Influence of washing step between blank and sample synthesis

Figure 16 shows results of performing the synthesis twice with identical parameters but with and without an intermediate washing step between the blank synthesis and the subsequent sample synthesis. This leads to slight differences in the scattering patterns.

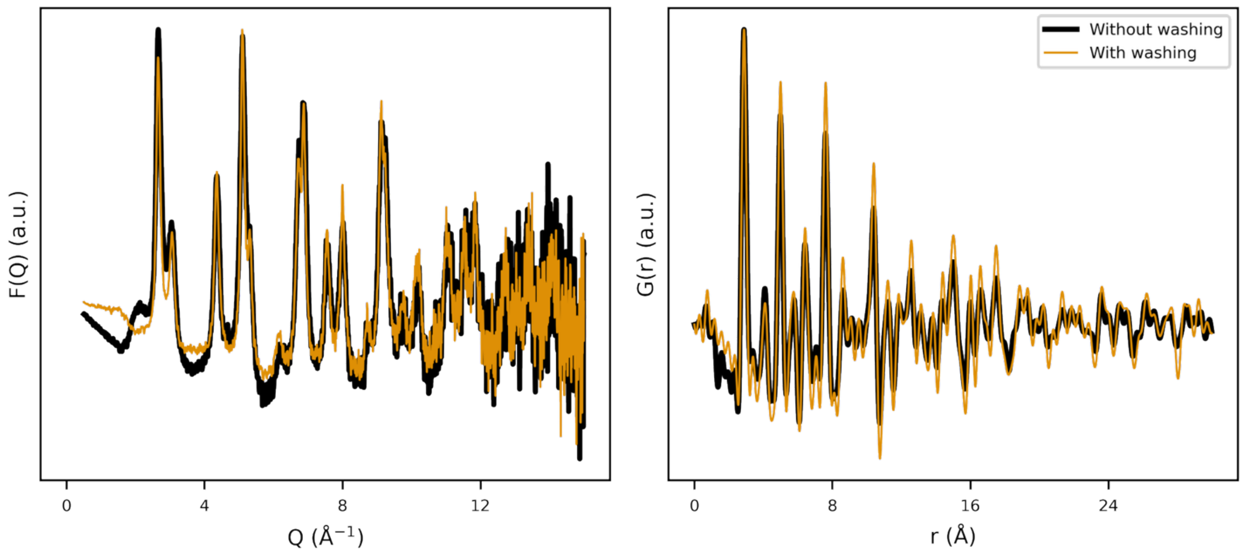

Figure S16 | Influence of washing between background and sample synthesis. Comparison of scattering patterns F(Q) (left) and G(r) (right) for two experiments under identical conditions but incorporating a washing step between the blank synthesis and the sample synthesis.

N: Determining whether a measurement is obtained on air or not

To distinguish air measurements from those involving blank or sample solutions, we compute a similarity metric between the experimental dataset and a reference "air" dataset, based on a MSE criterion. If the MSE lies below a user-defined threshold of 10^{-3}, the measurement is classified as air; otherwise, it is considered non-air (i.e.

blank or sample). Figure S17 illustrates these similarity values across 40 measurements. When two consecutive scans exceed the threshold, the system automatically switches to a longer, one-minute scan for the next measurement (instead of the default one-second scan). These one-minute scans provide definitive data for the SDL campaign, whereas the one-second scans primarily serve to confirm whether a blank or sample has been successfully moved into the beam.

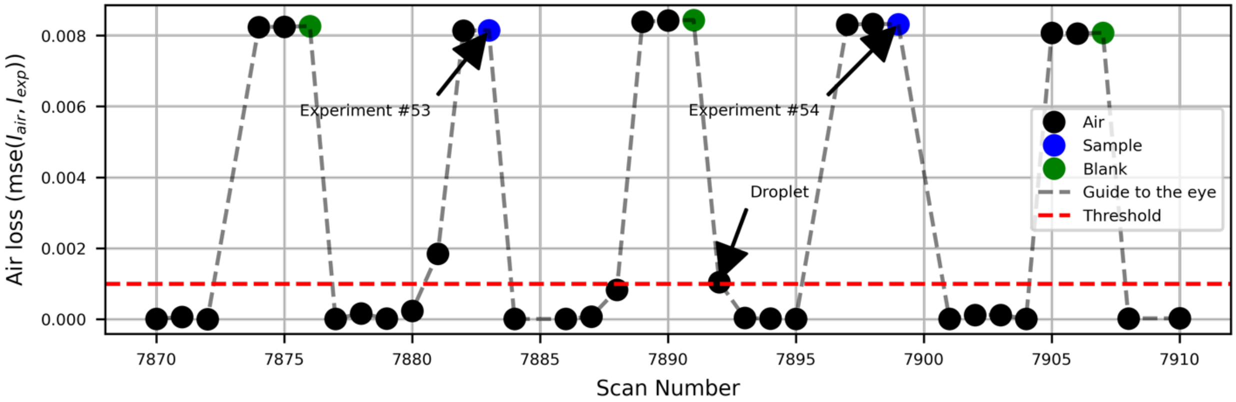

Figure S17 | Air similarity measures over 40 measurements. Air similarity values are shown for measurements at the synchrotron. Points below the user-defined threshold (10^{-3}, indicated in red) are classified as air, whereas two consecutive points above the threshold trigger a longer (1 minute) measurement instead of the standard 1 second acquisition.

O: Background subtraction of the scattering data

During data processing, the blank scattering pattern is subtracted from the sample scattering pattern to eliminate contributions from the solvent, air, and capillary. Figure S18 demonstrates the automated blank subtraction procedure for experiment # 56, showing the raw sample data (green), the blank (dashed red), and the resulting subtracted pattern (orange).

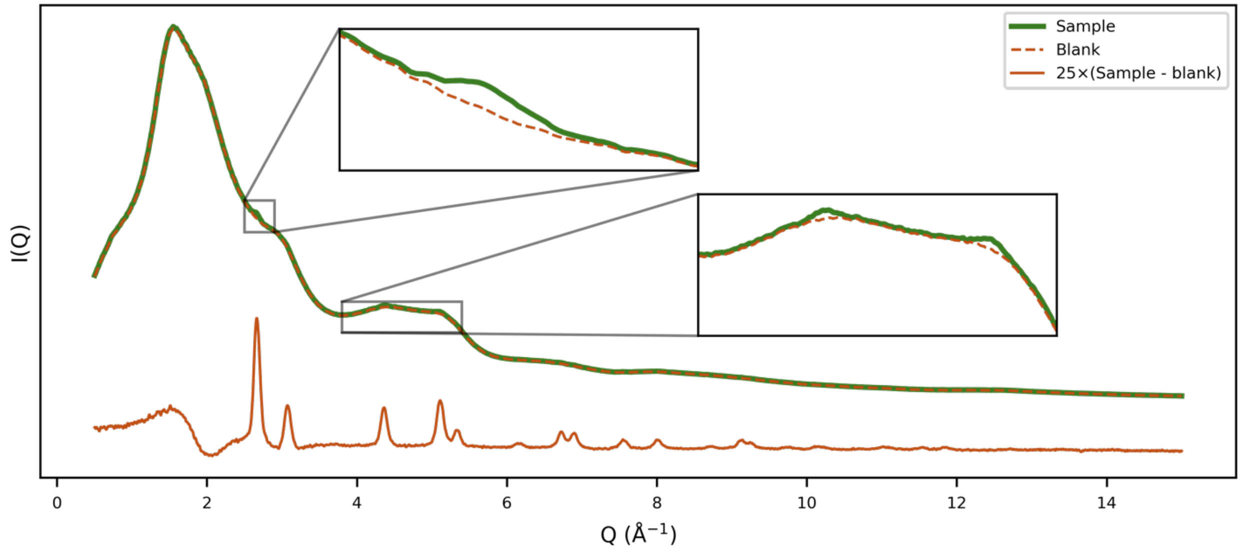

Figure S18 | Automated blank subtraction of experiment # 56.

P: Chemical insights from Gaussian Process (GP) surrogate modelling

GP predictions and cross-validation

To evaluate the effectiveness of our GP surrogate model, we performed cross-validation analyses. Figures S19 and S20 show predicted versus observed objective values obtained from the GP model through 3-fold, 5-fold, and 10-fold cross-validation schemes. In Figure S19, the GP model was trained using data from the initial 47 experiments, demonstrating a moderate correlation (R^2 values between ~0.52 and ~0.56) between predicted and actual objective values. Extending the training set to include all 56 experiments significantly improved the correlation (R^2 values increased to between ~0.64 and ~0.67), as illustrated in Figure S20. This improvement clearly indicates that our GP surrogate model effectively learns meaningful patterns from the experimental data, highlighting the presence of underlying chemical trends.

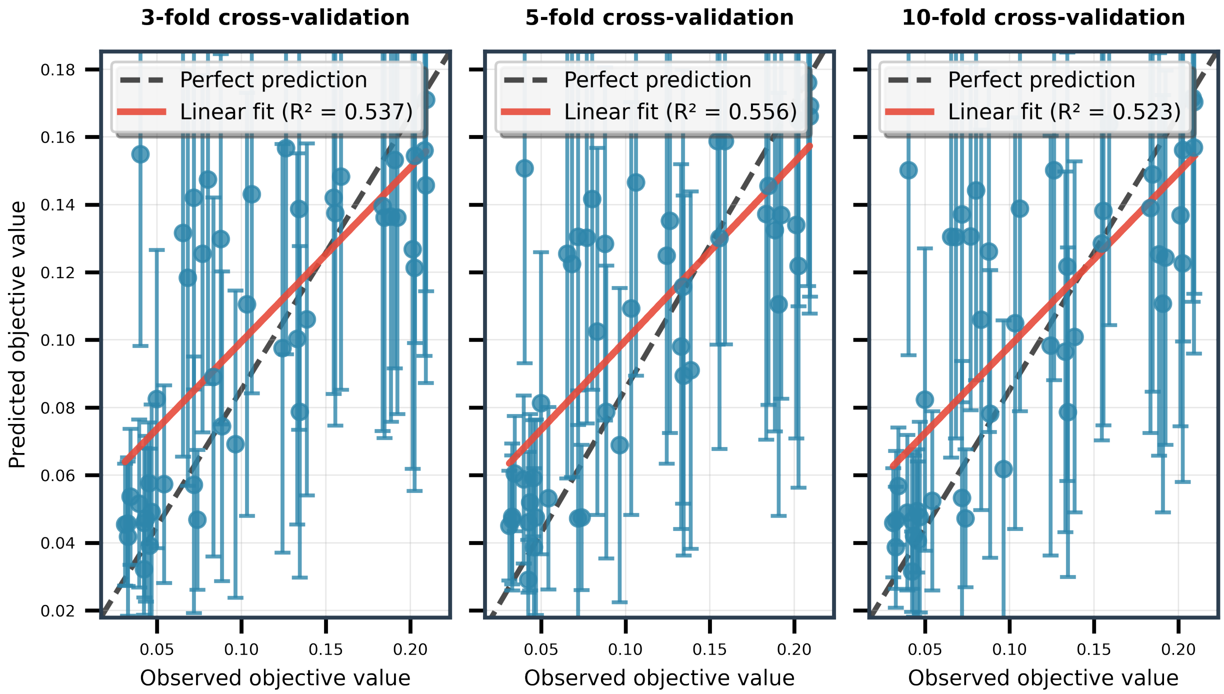

Figure S19 | Predictions of the GP trained on the first 47 experiments. Predicted versus observed objective values obtained from 3-fold, 5-fold, and 10-fold cross-validation.

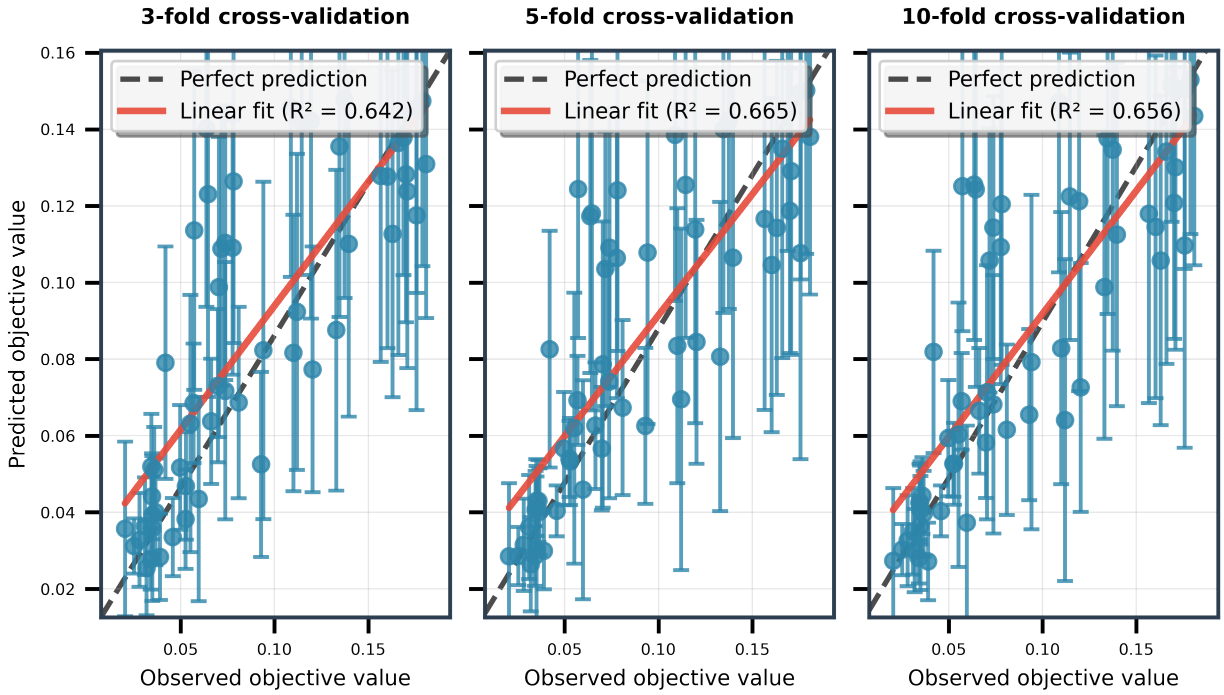

Figure S20 | Predictions of the GP trained on all 56 experiments. Predicted versus observed objective values obtained from 3-fold, 5-fold, and 10-fold cross-validation.

These findings support inherent chemical correlations within our data, allowing extraction of chemical insights from the surrogate model (though with some uncertainty given the limited accuracy of the GP model). We demonstrate this capability in two ways: (1) evaluating feature importance for each synthesis parameter, and (2) using the GP as a surrogate model to simulate experimental outcomes and identify parameters that preferentially yield either the ~5 nm decahedral or the 10 nm FCC AuNP structures.

Feature importance analysis

Figure S21 shows posterior samples of the kernel lengthscales, reflecting parameter importance according to the GP model. Lower lengthscales indicate parameters that the GP models estimate to have significant influence, whereas higher lengthscales imply minimal influence on the predicted objective. We observe that LED illumination, gold precursor addition speed, and mixing speed have relatively low importance. In contrast, chemical composition parameters—especially the amount of gold precursor—emerge as highly influential together with UV-B illumination.

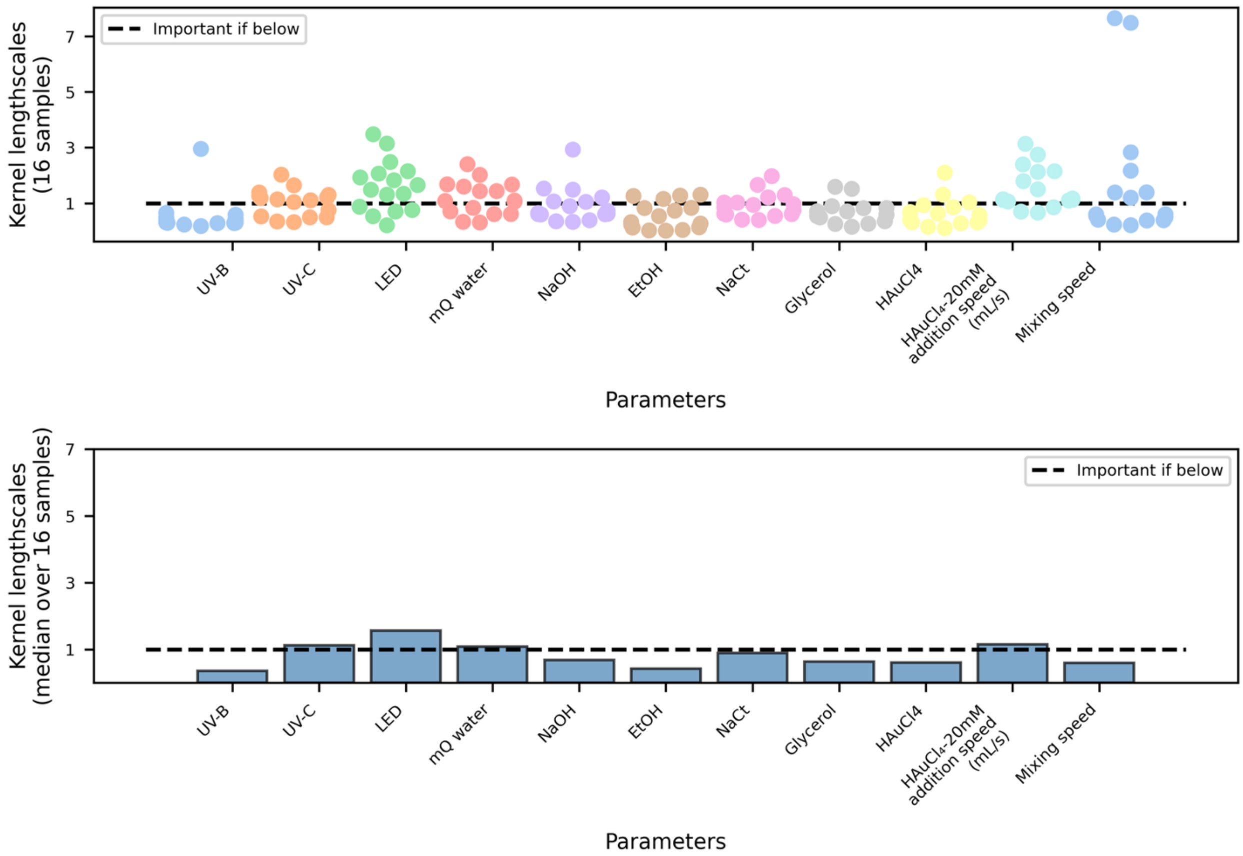

Figure S21 | Feature importance from GP kernel lengthscales. Posterior samples of kernel lengthscales indicating parameter importance. Top: Swarm plot representation, Bottom: Median representation. Lower lengthscales indicate higher importance. Dashed lines represent thresholds for "significant" (black) parameters to guide the eye.

GP as a surrogate for experimental exploration

We further employed the GP surrogate model to simulate hypothetical experiments. Initially, we performed 1000 random experiments (Figure S22), which generally yielded high objective values, suggesting low likelihood for randomly synthesising either of the ~5 nm decahedral or the 10 nm FCC AuNP structures. However, targeted exploration around the two optimised synthesis protocols (Figure S23) clearly revealed underlying chemical trends. Here, we varied each parameter systematically by up to ±25% (When changing chemical compositions,

all chemical compositions were proportionally re-normalised to maintain a total of 100%). Notably, actual experimental objective values (marked as stars) were consistently higher than the GP's predictions. Certain parameters indicated some room for optimisation, for example, increasing the UV–C illumination could further improve synthesis outcomes (lower the objective value).

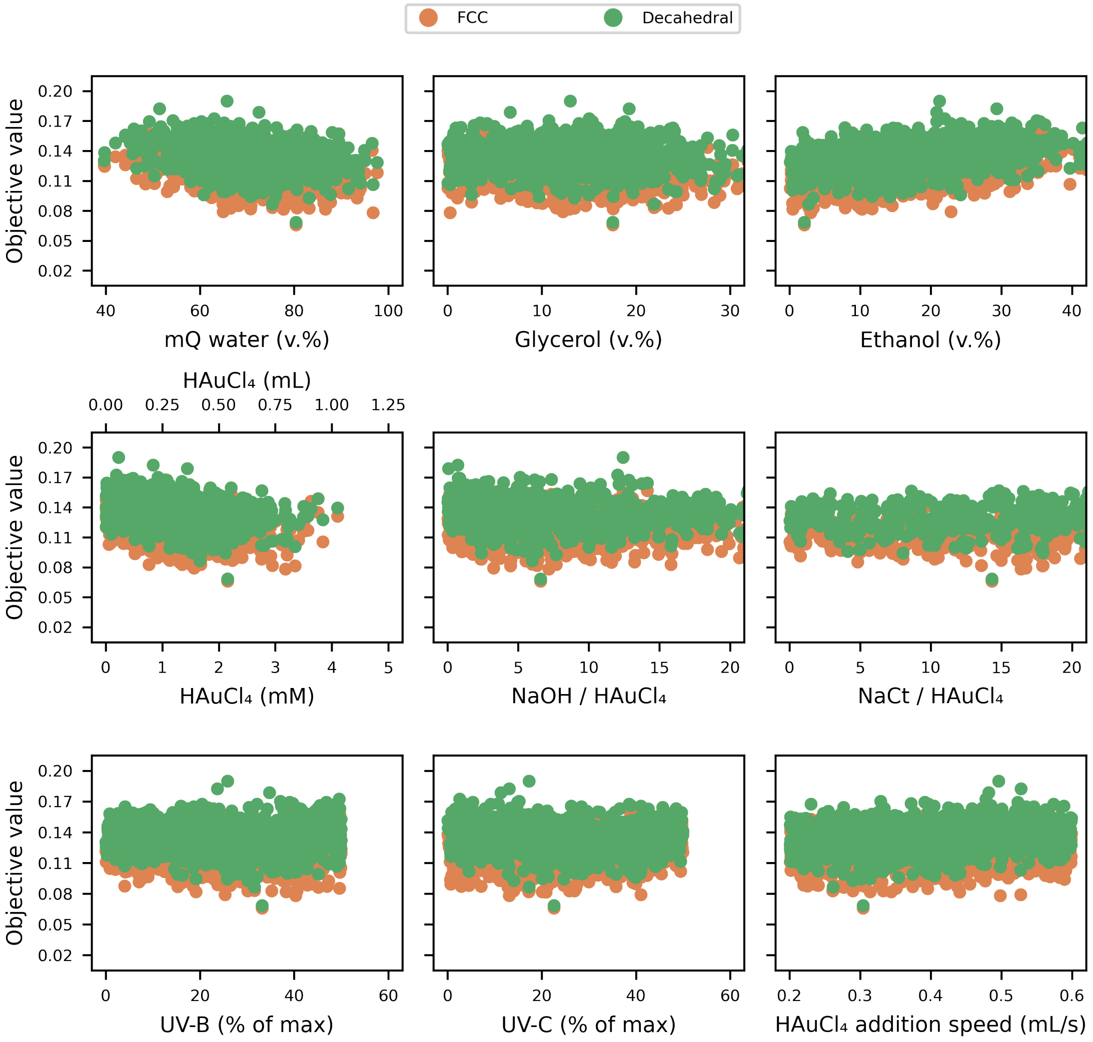

Figure S22 | Objective values from GP simulations using random synthesis parameters. Objective values obtained by randomly sampling the GP surrogate model for 1000 experiments, highlighting generally high values and low probability of optimal synthesis outcomes.

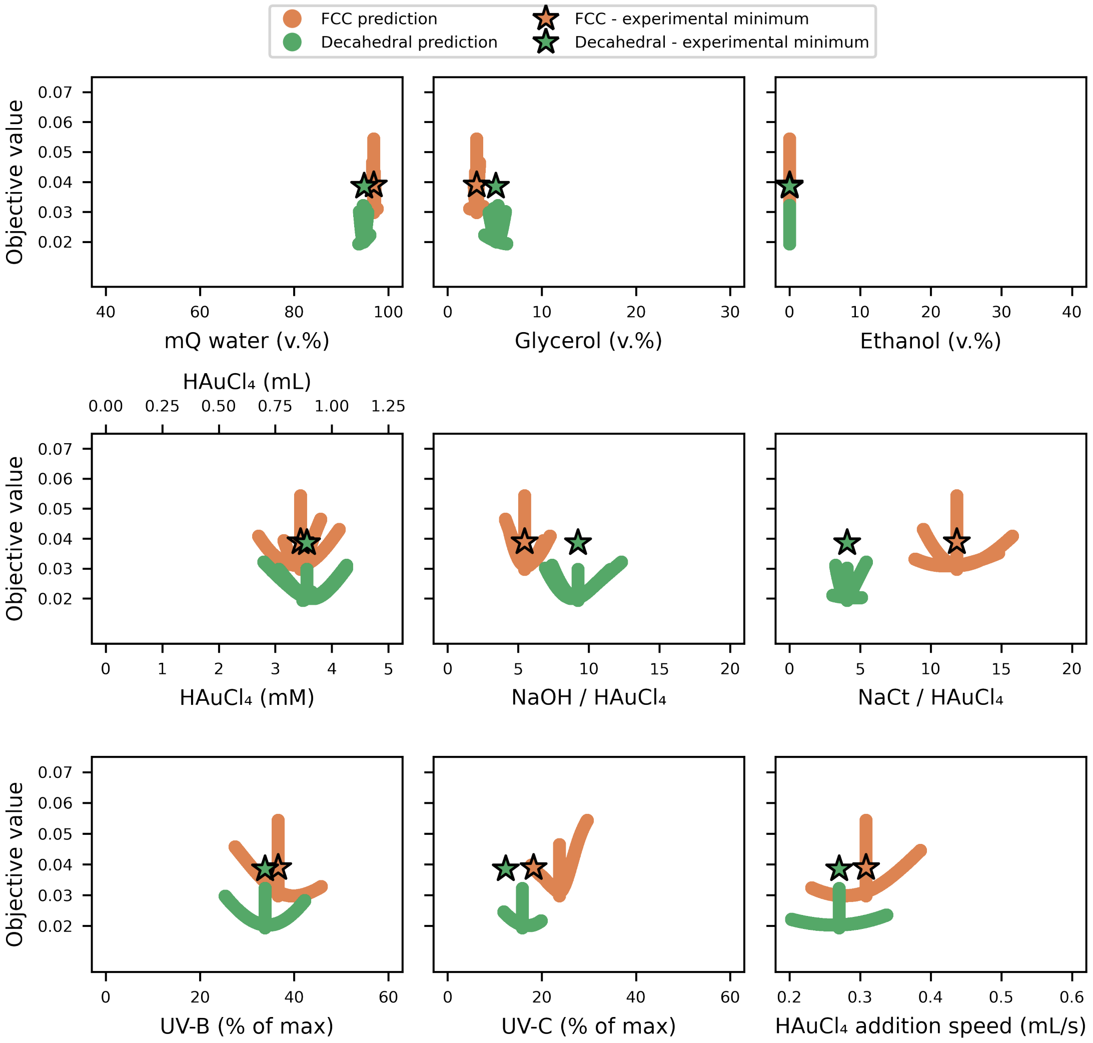

Figure S23 | GP surrogate predictions around optimised synthesis conditions. Objective values predicted by the GP model for systematic variations (±25%) around optimised synthesis conditions for the ~5 nm decahedral and 10 nm FCC AuNP structures. Stars denote actual experimental outcomes.

We acknowledge that cautious interpretation is necessary given our limited dataset (56 data points in an 11-dimensional parameter space). Nevertheless, the analysis confirms that the GP model learns some chemical trends. With larger datasets, we anticipate that the GP surrogate model would become more accurate and could guide chemists towards meaningful chemical insights.

Q: Photographs from the beamtime

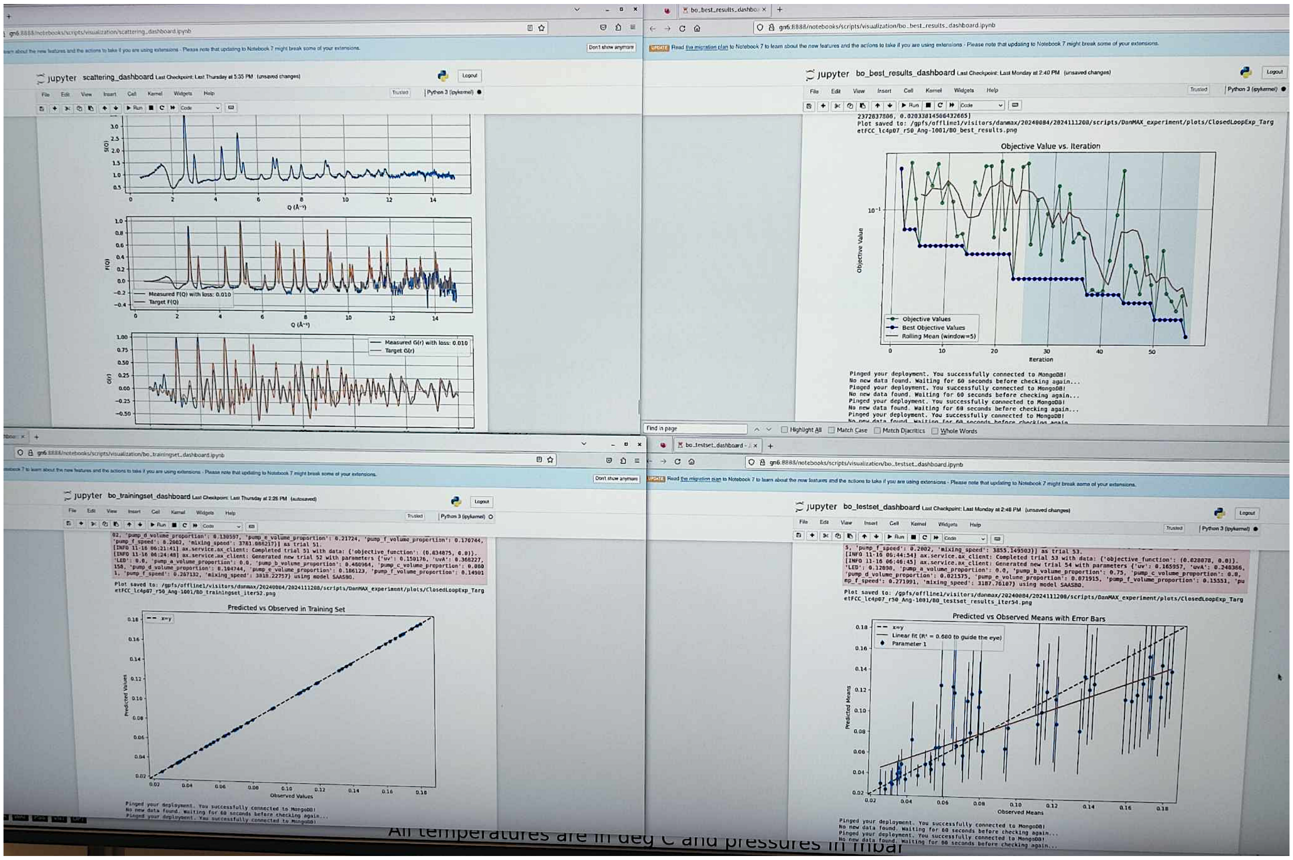

Figure S24 | Live plotting interface. A screenshot of the real-time data visualisation and BO outputs. **(Top left)** Live plots of the most recent scattering structure functions; the total scattering structure function, S(Q), the reduced total scattering function, F(Q), and the reduced atomic pair distribution function, G(r). **(Top right)** A running plot of objective values for each completed experiment. **(Bottom left)** BO surrogate model predictions versus actual objective values for the training set. **(Bottom right)** BO surrogate model predictions versus actual objective values for a held-out test set.

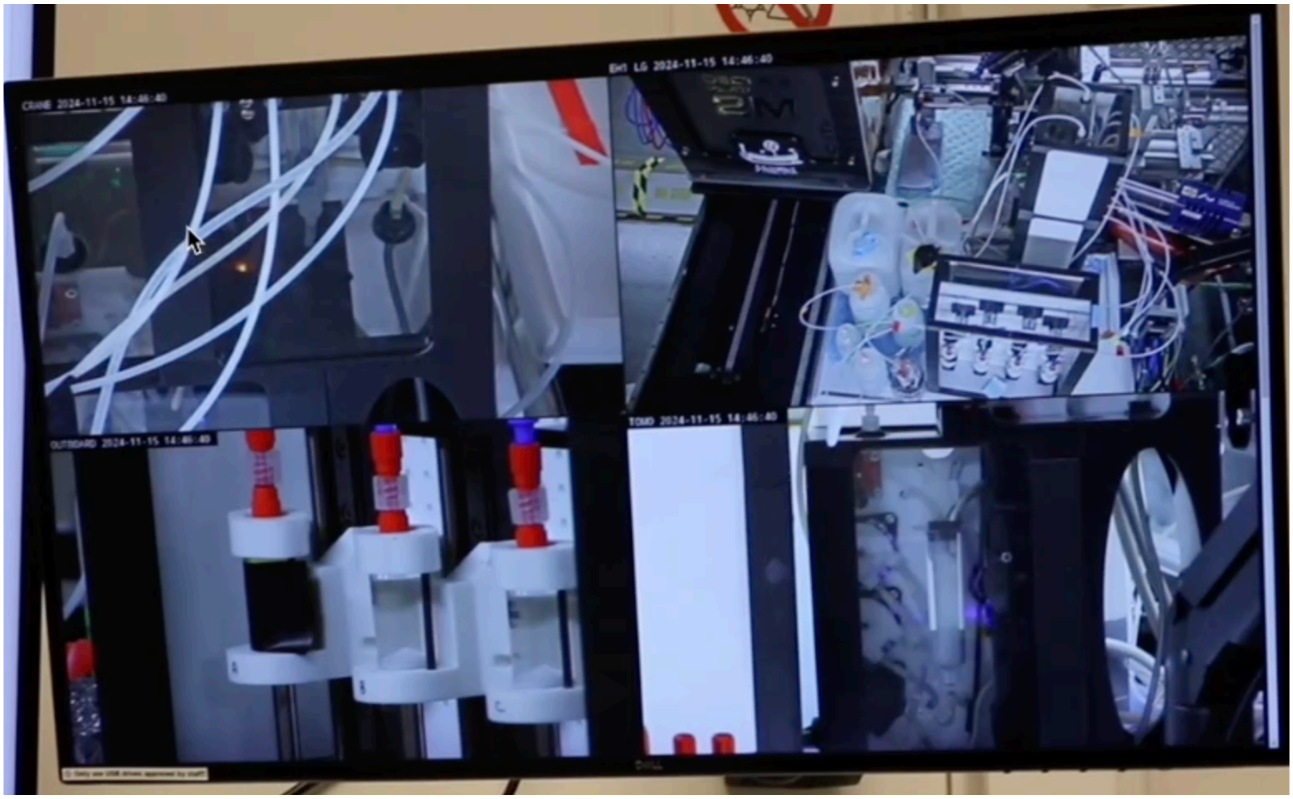

Figure S25 | Camera view of the robotic synthesis setup. Video feed showing different angles of the modular platform at the synchrotron. **(Top left)** The mixing module in operation. **(Top right)** A top-down perspective of the chemicals, the integrated synthesis system, the capillary, and the detector. **(Bottom left)** The syringe module responsible for delivering precise volumes of reagents. **(Bottom right)** The white/UV LED illumination module.

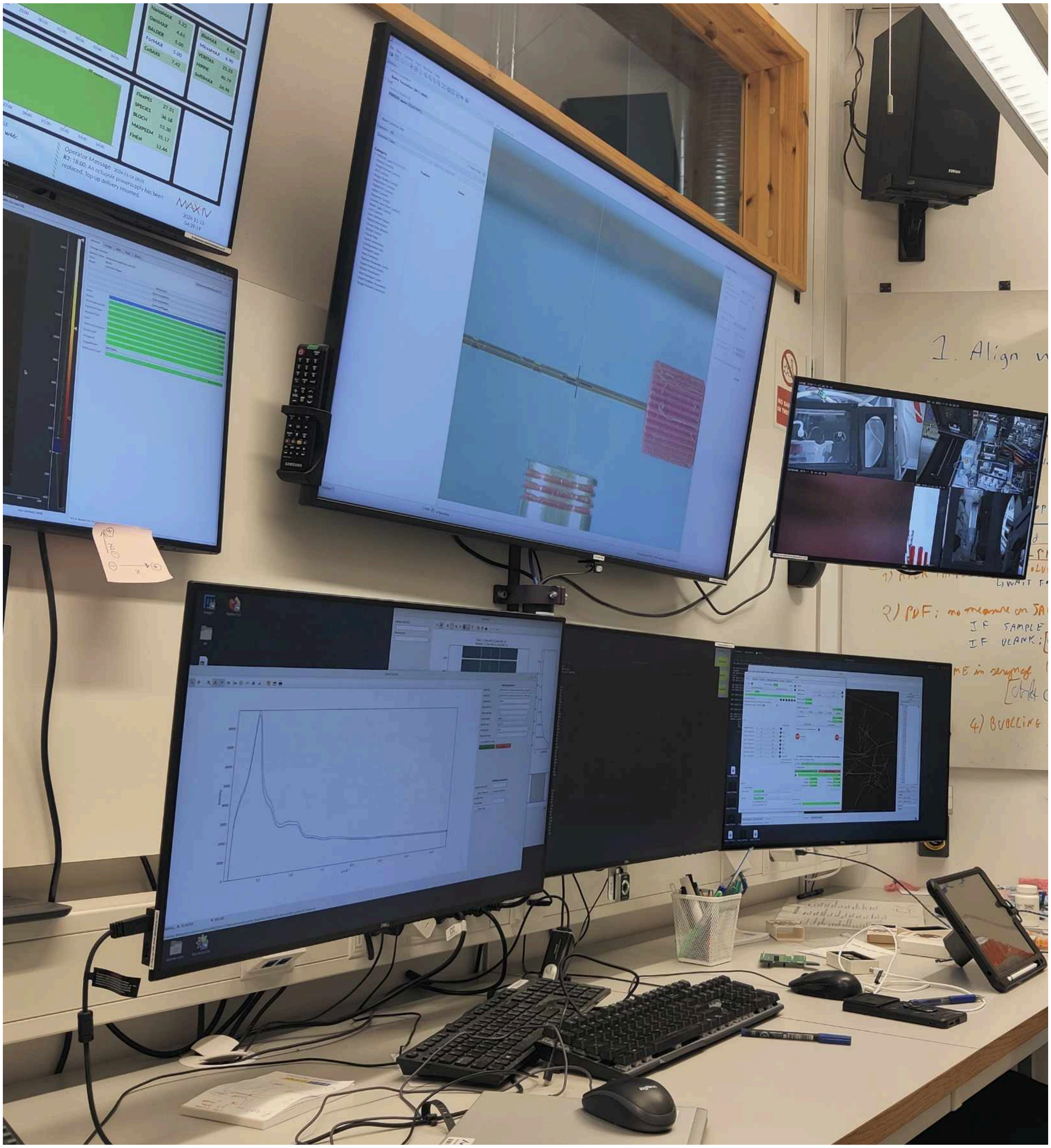

Figure S26 | Control hutch during beamtime operations. A photograph of the control hutch, where live data are monitored and the SDL workflow is supervised.

References

(1) Salley, D.; Keenan, G.; Grizou, J.; Sharma, A.; Martín, S.; Cronin, L. A nanomaterials discovery robot for the Darwinian evolution of shape programmable gold nanoparticles. *Nat. Commun.* **2020**, *11* (1), 2771. DOI: 10.1038/s41467-020-16501-4.

(2) Tao, H.; Wu, T.; Kheiri, S.; Aldeghi, M.; Aspuru-Guzik, A.; Kumacheva, E. Self-Driving Platform for Metal Nanoparticle Synthesis: Combining Microfluidics and Machine Learning. *Adv. Funct. Mater.* **2021**, *31* (51), 2106725. DOI: 10.1002/adfm.202106725.

(3) Jiang, Y.; Salley, D.; Sharma, A.; Keenan, G.; Mullin, M.; Cronin, L. An artificial intelligence enabled chemical synthesis robot for exploration and optimization of nanomaterials. *Sci. Adv.* **2022**, *8* (40), eabo2626. DOI: 10.1126/sciadv.abo2626.

(4) Zhao, H.; Chen, W.; Huang, H.; Sun, Z.; Chen, Z.; Wu, L.; Zhang, B.; Lai, F.; Wang, Z.; Adam, M. L.; et al. A robotic platform for the synthesis of colloidal nanocrystals. *Nat. Synth.* **2023**, *2* (6), 505-514. DOI: 10.1038/s44160-023-00250-5.

(5) Wu, T.; Kheiri, S.; Hickman, R. J.; Tao, H.; Wu, T. C.; Yang, Z.-B.; Ge, X.; Zhang, W.; Abolhasani, M.; Liu, K.; et al. Self-driving lab for the photochemical synthesis of plasmonic nanoparticles with targeted structural and optical properties. *Nat. Commun.* **2025**, *16* (1), 1473. DOI: 10.1038/s41467-025-56788-9.

(6) Vaddi, K.; Chiang, H. T.; Grey, A.; Wylie, Z. R.; Pozzo, L. D. Autonomous phase mapping of gold nanoparticles synthesis with differentiable models of spectral shape. *npj Comput. Mater.* **2025**, *11* (1), 335. DOI: 10.1038/s41524-025-01822-z.

(7) Daniel, M.-C.; Astruc, D. Gold Nanoparticles: Assembly, Supramolecular Chemistry, Quantum-Size-Related Properties, and Applications toward Biology, Catalysis, and Nanotechnology. *Chem. Rev.* **2004**, *104* (1), 293-346. DOI: 10.1021/cr030698+.

(8) Daruich De Souza, C.; Ribeiro Nogueira, B.; Rostelato, M. E. C. M. Review of the methodologies used in the synthesis gold nanoparticles by chemical reduction. *J. Alloys Compd.* **2019**, *798*, 714-740. DOI: 10.1016/j.jallcom.2019.05.153.

(9) Khan, T.; Ullah, N.; Khan, M. A.; Mashwani, Z.-u.-R.; Nadhman, A. Plant-based gold nanoparticles; a comprehensive review of the decade-long research on synthesis, mechanistic aspects and diverse applications. *Adv. Colloid Interface Sci.* **2019**, *272*, 102017. DOI: 10.1016/j.cis.2019.102017.

(10) Wuithschick, M.; Birnbaum, A.; Witte, S.; Sztucki, M.; Vainio, U.; Pinna, N.; Rademann, K.; Emmerling, F.; Kraehnert, R.; Polte, J. Turkevich in New Robes: Key Questions Answered for the Most Common Gold Nanoparticle Synthesis. *ACS Nano* **2015**, *9* (7), 7052-7071. DOI: 10.1021/acsnano.5b01579.

(11) Xue, Y.; Li, X.; Li, H.; Zhang, W. Quantifying thiol–gold interactions towards the efficient strength control. *Nat. Commun.* **2014**, *5* (1), 4348. DOI: 10.1038/ncomms5348.

(12) Deraedt, C.; Salmon, L.; Gatard, S.; Ciganda, R.; Hernandez, R.; Ruiz, J.; Astruc, D. Sodium borohydride stabilizes very active gold nanoparticle catalysts. *Chem. Commun.* **2014**, *50* (91), 14194-14196. DOI: 10.1039/C4CC05946H.

(13) Quinson, J.; Aalling-Frederiksen, O.; Dacayan, W. L.; Bjerregaard, J. D.; Jensen, K. D.; Jørgensen, M. R. V.; Kantor, I.; Sørensen, D. R.; Theil Kuhn, L.; Johnson, M. S.; et al. Surfactant-Free Colloidal Syntheses of Gold-Based Nanomaterials in Alkaline Water and Mono-alcohol Mixtures. *Chem. Mater.* **2023**, *35* (5), 2173-2190. DOI: 10.1021/acs.chemmater.3c00090.

(14) Malassis, L.; Dreyfus, R.; Murphy, R. J.; Hough, L. A.; Donnio, B.; Murray, C. B. One-step green synthesis of gold and silver nanoparticles with ascorbic acid and their versatile surface post-functionalization. *RSC Adv.* **2016**, *6* (39), 33092-33100. DOI: 10.1039/C6RA00194G.

(15) Scarabelli, L.; Sánchez-Iglesias, A.; Pérez-Juste, J.; Liz-Marzán, L. M. A "Tips and Tricks" Practical Guide to the Synthesis of Gold Nanorods. *J. Phys. Chem.* **2015**, *6* (21), 4270-4279. DOI: 10.1021/acs.jpclett.5b02123.

(16) Røjkjær Rasmussen, D.; Lock, N.; Quinson, J. Lights on the Synthesis of Surfactant-Free Colloidal Gold Nanoparticles in Alkaline Mixtures of Alcohols and Water. *ChemSusChem* **2025**, *18* (3), e202400763. DOI: 10.1002/cssc.202400763.
(17) Jensen, T. B.; Saugbjerg, J. R.; Henriksen, M. L.; Quinson, J. Towards the automation of nanoparticle syntheses: The case study of gold nanoparticles obtained at room temperature. *Colloids Surf. A: Physicochem. Eng. Asp.* **2024**, *702*, 135125. DOI: 10.1016/j.colsurfa.2024.135125.
(18) Grzelczak, M.; Pérez-Juste, J.; Mulvaney, P.; Liz-Marzán, L. M. Shape control in gold nanoparticle synthesis. *Chem. Soc. Rev.* **2008**, *37* (9), 1783-1791. DOI: 10.1039/B711490G.
(19) Wall, M. A.; Harmsen, S.; Pal, S.; Zhang, L.; Arianna, G.; Lombardi, J. R.; Drain, C. M.; Kircher, M. F. Surfactant-Free Shape Control of Gold Nanoparticles Enabled by Unified Theoretical Framework of Nanocrystal Synthesis. *Adv. Mater.* **2017**, *29* (21), 1605622. DOI: 10.1002/adma.201605622.
(20) Borowskaja, D. Zur Methodik der Goldsolbereitung. *Ztschr. f. Immunitatsforsch. u. exper. Therap* **1934**, *82*, 178-182.
(21) Turkevich, J.; Stevenson, P. C.; Hillier, J. A study of the nucleation and growth processes in the synthesis of colloidal gold. *Discussions of the Faraday Society* **1951**, *11* (0), 55-75, 10.1039/DF9511100055. DOI: 10.1039/DF9511100055.
(22) Brust, M.; Walker, M.; Bethell, D.; Schiffrin, D. J.; Whyman, R. Synthesis of thiol-derivatised gold nanoparticles in a two-phase Liquid–Liquid system. *J. Chem. Soc., Chem. Commun.* **1994**, (7), 801-802. DOI: 10.1039/C39940000801.
(23) Liz-Marzán, L. M. Gold nanoparticle research before and after the Brust–Schiffrin method. *Chem. Commun.* **2013**, *49* (1), 16-18. DOI: 10.1039/C2CC35720H.
(24) Baird, S.; Ansari, M.; Afzal, Z.; Ai, Q.; Al-Feghali, A.; Alain, M.; Altamirano, M.; Andrews, T.; Anker, A. S.; Ansari, R. Bayesian Optimization Hackathon for Chemistry and Materials. **2025**.
(25) Jensen, K. M. Ø.; Juhas, P.; Tofanelli, M. A.; Heinecke, C. L.; Vaughan, G.; Ackerson, C. J.; Billinge, S. J. L. Polymorphism in magic-sized $Au_{144}(SR)_{60}$ clusters. *Nat. Commun.* **2016**, *7*, 11859. DOI: 10.1038/ncomms11859.
(26) Sedano Varo, E.; Egeberg Tankard, R.; Kryger-Baggesen, J.; Jinschek, J.; Helveg, S.; Chorkendorff, I.; Damsgaard, C. D.; Kibsgaard, J. Gold Nanoparticles for CO_2 Electroreduction: An Optimum Defined by Size and Shape. *J. Am. Chem. Soc.* **2024**, *146* (3), 2015-2023. DOI: 10.1021/jacs.3c10610.
(27) Rahm, J. M.; Erhart, P. Beyond Magic Numbers: Atomic Scale Equilibrium Nanoparticle Shapes for Any Size. *Nano Lett.* **2017**, *17* (9), 5775-5781. DOI: 10.1021/acs.nanolett.7b02761.
(28) Eriksson, D.; Jankowiak, M. High-dimensional Bayesian optimization with sparse axis-aligned subspaces. *Uncertainty in Artificial Intelligence* **2021**, 493-503.
(29) Hvarfner, C.; Hellsten, E. O.; Nardi, L. Vanilla Bayesian Optimization Performs Great in High Dimension. *Proceedings of the 41st International Conference on Machine Learning* **2024**, *235*, 20793-20817.
(30) Santoni, M. L.; Raponi, E.; Leone, R. D.; Doerr, C. Comparison of high-dimensional bayesian optimization algorithms on bbob. *ACM Transactions on Evolutionary Learning* **2024**, *4* (3), 1-33.
(31) Juhas, P.; Davis, T.; Farrow, C. L.; Billinge, S. J. L. PDFgetX3: a rapid and highly automatable program for processing powder diffraction data into total scattering pair distribution functions. *J. Appl. Cryst.* **2013**, *46* (2), 560-566. DOI: 10.1107/S0021889813005190.
(32) Canty, R. B.; Abolhasani, M. Reproducibility in automated chemistry laboratories using computer science abstractions. *Nat. Synth.* **2024**, *3* (11), 1327-1339. DOI: 10.1038/s44160-024-00649-8.
(33) Bergman, R. G.; Danheiser, R. L. Reproducibility in Chemical Research. *Angew. Chem. Int. Ed.* **2016**, *55* (41), 12548-12549. DOI: 10.1002/anie.201606591.

(34) Schäfer, F.; Lückemeier, L.; Glorius, F. Improving reproducibility through condition-based sensitivity assessments: application, advancement and prospect. *Chem. Sci.* **2024**, *15* (36), 14548-14555. DOI: 10.1039/D4SC03017F.
(35) Baker, M. 1,500 scientists lift the lid on reproducibility. *Nature* **2016**, *533* (7604), 452-454. DOI: 10.1038/533452a.
(36) Johansen, F. L.; Anker, A. S.; Friis-Jensen, U.; Dam, E. B.; Selvan, R. A GPU-Accelerated Open-Source Python Package for Calculating Powder Diffraction, Small-Angle-, and Total Scattering with the Debye Scattering Equation. *JOSS* **2024**, *9* (94), 6024. DOI: 10.21105/joss.06024.
(37) Varga, M.; Quinson, J. Fewer, but Better: On the Benefits of Surfactant-Free Colloidal Syntheses of Nanomaterials. *ChemistrySelect* **2025**, *10* (5), e202404819. DOI: 10.1002/slct.202404819.
(38) Quinson, J. Room Temperature Surfactant-Free Syntheses of Gold Nanoparticles in Alkaline Mixtures of Water and Alcohols: A Model System to Introduce Nanotechnology and Green Chemistry to Future Chemists and Engineers. *J. Chem. Educ.* **2023**, *100* (9), 3612-3619. DOI: 10.1021/acs.jchemed.3c00492.
(39) Panariello, L.; Radhakrishnan, A. N. P.; Papakonstantinou, I.; Parkin, I. P.; Gavriilidis, A. Particle Size Evolution during the Synthesis of Gold Nanoparticles Using In Situ Time-Resolved UV–Vis Spectroscopy: An Experimental and Theoretical Study Unravelling the Effect of Adsorbed Gold Precursor Species. *J. Phys. Chem. C* **2020**, *124* (50), 27662-27672. DOI: 10.1021/acs.jpcc.0c07405.
(40) Haiss, W.; Thanh, N. T. K.; Aveyard, J.; Fernig, D. G. Determination of Size and Concentration of Gold Nanoparticles from UV−Vis Spectra. *Anal. Chem.* **2007**, *79* (11), 4215-4221. DOI: 10.1021/ac0702084.
(41) Ye, Y.; Lv, M.; Zhang, X.; Zhang, Y. Colorimetric determination of copper(ii) ions using gold nanoparticles as a probe. *RSC Adv.* **2015**, *5* (124), 102311-102317. DOI: 10.1039/C5RA20381C.
(42) Merk, V.; Rehbock, C.; Becker, F.; Hagemann, U.; Nienhaus, H.; Barcikowski, S. In Situ Non-DLVO Stabilization of Surfactant-Free, Plasmonic Gold Nanoparticles: Effect of Hofmeister's Anions. *Langmuir* **2014**, *30* (15), 4213-4222. DOI: 10.1021/la404556a.
(43) Hendel, T.; Wuithschick, M.; Kettemann, F.; Birnbaum, A.; Rademann, K.; Polte, J. In Situ Determination of Colloidal Gold Concentrations with UV–Vis Spectroscopy: Limitations and Perspectives. *Anal. Chem.* **2014**, *86* (22), 11115-11124. DOI: 10.1021/ac502053s.
(44) Volk, A. A.; Abolhasani, M. Performance metrics to unleash the power of self-driving labs in chemistry and materials science. *Nat. Commun.* **2024**, *15* (1), 1378. DOI: 10.1038/s41467-024-45569-5.
(45) Sjølin, B. H.; Hansen, W. S.; Morin-Martinez, A. A.; Petersen, M. H.; Rieger, L. H.; Vegge, T.; García-Lastra, J. M.; Castelli, I. E. PerQueue: managing complex and dynamic workflows. *Digital Discovery* **2024**, *3* (9), 1832-1841. DOI: 10.1039/D4DD00134F.
(46) Jain, A.; Ong, S. P.; Chen, W.; Medasani, B.; Qu, X.; Kocher, M.; Brafman, M.; Petretto, G.; Rignanese, G.-M.; Hautier, G.; et al. FireWorks: a dynamic workflow system designed for high-throughput applications. *Concurr. Comput. Pract. Exp.* **2015**, *27* (17), 5037-5059. DOI: 10.1002/cpe.3505.
(47) Rosen, A. S.; Gallant, M.; George, J.; Riebesell, J.; Sahasrabuddhe, H.; Shen, J.-X.; Wen, M.; Evans, M. L.; Petretto, G.; Waroquiers, D. Jobflow: Computational workflows made simple. *JOSS* **2024**, *9* (93), 5995. DOI: 10.21105/joss.05995.
